# Gas-chromatographic headspace analysis in human biomonitoring (headspace-gas chromatography): Biomonitoring Methods, Conceptual Topics – Translation of the German version from 2025

**DOI:** 10.34865/bihsgcegt10_3or

**Published:** 2025-09-29

**Authors:** Michael Bader, Bernd Roßbach, Thomas Göen, Elisabeth Eckert, Anja Schäferhenrich, Stefanie Nübler, Wolfgang Gries, Gabriele Leng, Jan Van Pul, Wolfgang Will, Andrea Hartwig, MAK Commission

**Affiliations:** 1 BASF SE. Corporate Health Management Carl-Bosch-Straße 38 67056 Ludwigshafen Germany; 2 Institute of Occupational. Social and Environmental Medicine. University Medical Center of the Johannes Gutenberg-University Mainz Obere Zahlbacher Straße 67 55131 Mainz Germany; 3 Friedrich-Alexander-Universität Erlangen-Nürnberg. Institute and Outpatient Clinic of Occupational, Social, and Environmental Medicine Henkestraße 9–11 91054 Erlangen Germany; 4 Currenta GmbH & Co. OHG. CUR-SIT-SER-GS-BLM – Institute for Biomonitoring Chempark, Building Q18 51368 Leverkusen Germany; 5 BASF Antwerpen N.V. Haven 725, Scheldelaan 600 2040 Antwerpen Belgium; 6 Institute of Applied Biosciences. Department of Food Chemistry and Toxicology. Karlsruhe Institute of Technology (KIT) Adenauerring 20a, Building 50.41 76131 Karlsruhe Germany; 7 Permanent Senate Commission for the Investigation of Health Hazards of Chemical Compounds in the Work Area. Deutsche Forschungsgemeinschaft, Kennedyallee 40, 53175 Bonn, Germany. Further information: Permanent Senate Commission for the Investigation of Health Hazards of Chemical Compounds in the Work Area | DFG

**Keywords:** headspace gas chromatography, HS-GC, biomonitoring, urine, blood, serum, plasma, headspace, headspace technique, half-life

## Abstract

The working group “Analyses in Biological Materials” of the German Senate Commission for the Investigation of Health Hazards of Chemical Compounds in the Work Area (MAK Commission) describes the current status of headspace-gas chromatography with respect to its potential applications in human biomonitoring. Particular focus is given to the review and discussion of newly developed methods for headspace sample collection as well as analyte enrichment. The article gives an overview on internationally published headspace methods for the matrices urine, blood, serum and plasma, existing assessment values for headspace parameters, background exposure levels in the non-occupationally exposed general population as well as half-lives of the most prominent hazardous substances measurable by headspace methods. In addition, critical requirements for and possible pitfalls of the preanalytical phase and of the calibration of headspace analyses are also discussed. The review shows that headspace methods have been continuously improved in recent decades and thus continue to make an important contribution to human biomonitoring of occupational and environmental exposure to volatile hazardous compounds.

## 1 Introduction

Human biomonitoring (HBM, see also List of abbreviations) is generally defined as the investigation of human bio­logical materials for the determination of hazardous substances or their metabolites or of effect parameters in order to detect and assess exposure and potential health hazards. Furthermore, the results of HBM in the workplace can provide important information for the assessment of the efficacy of occupational health and safety measures (AfAMed 2015). In population-based HBM programmes, environmental and lifestyle-related exposure to hazardous substances is investigated, and temporal as well as geographical trends can be identified (e.g. Schwedler et al. 2019). For this purpose, suitable methods of chemical analysis are necessary with which the target substances, which are often present only in very small concentrations, can be extracted from the biological matrix and subsequently determined both specifically and sensitively.

Gas-chromatographic headspace analysis, hereafter simplified as “headspace analysis” (or headspace-gas chromato­graphy, headspace‑GC, headspace technique), represents an especially suitable procedure for the efficient separation of volatile target compounds from the biological matrix as well as for subsequent sensitive determination. Headspace analysis enables the simultaneous measurement of a broad spectrum of parameters within different substance groups, usually without laborious sample preparation or derivatisation (Ikeda 1999).

For headspace analysis, the sample material is heated in a sealed, gas-tight sample vial, usually to a temperature in the range of 40 to 80 °C. During this process, volatile compounds accumulate in the headspace above the liquid sample and are thereby separated from the biological matrix. Once vapour-liquid equilibrium has been reached, an aliquot of the gas phase is extracted and analysed by gas chromatography. In this way, a range of organic solvents such as aliphatic and aromatic hydrocarbons, halogenated hydrocarbons, alcohols, ketones, ethers and esters can usually be determined without interferences. In contrast to the injection of liquid sample extracts, headspace analysis involves a relatively low transfer of matrix components into the chromatographic system and the detector. In principle, the reduced background noise thereby achieved enables low quantitation limits, allowing for the detection of analytes in the background range of the non-occupationally exposed general population. Furthermore, the contamination of the gas-chromatographic system with matrix components is lower, meaning that the service life before cleaning or maintenance is increased.

The headspace-analysis procedure was developed in the USA from the late 1950s to the early 1960s for the analysis of flavouring, odourous, and aromatic substances (Bassette et al. 1962; Buttery and Teranishi 1961; Mackay et al. 1961; Teranishi et al. 1962). A few years later, the procedure was successfully applied for the first time to determine the blood alcohol content (Machata 1964, 1967). Aside from the determination of ethanol, headspace analysis was initially applied to ascertain the solubility of anaesthetics (Butler et al. 1967; Fink and Morikawa 1970; Purchase 1963; Yamamura et al. 1966) as well as for the determination of gases (Curry et al. 1962; Dominguez et al. 1959; Galla and Ottenstein 1962; Hamilton 1962; Ramsey 1959), further alcohols (Machata 1964), and solvents (Goldbaum et al. 1964). Since then, headspace analysis with various modifications has been established in different areas of research and application; it has become a standard procedure in forensic chemistry, clinical chemistry, environmental chemistry, food chemistry, and polymer research (Wang et al. 2008).

Due to the varied areas of application, there is comprehensive literature on the fundamentals, method development, and application of headspace analysis. Thus, the theory and practice of “static” headspace analysis is thoroughly described by Hachenberg and Schmidt (1977), Ioffe and Vitenberg (1984), as well as Kolb and Ettre (2006). Moreover, textbooks on gas chromatography often contain sections on various headspace techniques (Grob and Barry 2004; McNair et al. 2019; Poole 2012). Furthermore, several review articles on headspace analysis have been published (see literature cited by Kolb and Ettre 2006), whereby the works of Seto (1994) and of Mills and Walker (2000) specifically discuss the determination of volatile substances in biological samples.

Since 1977, headspace methods have been developed, verified, and published by the “Analyses in Biological Materials” working group of the Permanent Senate Commission for the Investigation of Health Hazards of Chemical Compounds in the Work Area (MAK Commission) with the explicit purpose of HBM in occupational medicine. These methods cover a wide range of prominent industrial solvents. In addition to a total of 24 parameters summarised as part of a collective method (Machata and Angerer 1983), further headspace methods for specific substance groups have been published, such as for the determination of alcohols and ketones (Angerer et al. 1997), halogenated aliphatics (Angerer et al. 1991), halogenated aromatics (Lewalter et al. 1991), and BTEX aromatics (benzene, toluene, ethylbenzene, and the xylene isomers) (Angerer et al. 1994).

Due to innovations in instrumental analysis, it has become necessary to revise and update the analytical methods published by the Commission. As such, since 2006 – and, beginning in 2017, with renewed emphasis – the “MAK Collection online” has published methods on the determination of volatile hazardous substances in which headspace‑GC combined with mass-spectrometric (MS) detection is used as an especially sensitive and specific procedure to determine target analytes. As part of this process, a method for the determination of methylmercury in blood (Hoppe and Heinrich-Ramm 2006) has been published as well as methods for the determination of tetra­hydro­furan (THF) in urine (Blaszkewicz and Angerer 2013), tri­chloro­acetic acid in urine (Will et al. 2017), methyl *tert*‑butyl ether (MTBE) in blood and urine (Hoppe et al. 2018), aromatic compounds in blood (Göen et al. 2018), aromatic compounds in urine (Van Pul et al. 2018), 1‑bromopropane and 2‑bromopropane in urine (Roßbach et al. 2019), alcohols, ketones, and ethers in urine (Göen et al. 2020), as well as for the determination of chlorinated hydrocarbons in blood (Göen et al. 2021).

## 2 Fundamental principles of the headspace technique

In the following section, the basic principles of the headspace technique are briefly presented. For further details on this topic, please refer to Kremser et al (2016). There, also a systematic comparison of static and dynamic headspace techniques was carried out and the effect of the respective technique on the precision and detection limit for the determination of various analytes was also analysed.

### 2.1 Static headspace technique

In static headspace analysis, the gas phase of a (generally aqueous) sample is investigated once the phase equilibrium has been established. To this end, the sample is transferred into a suitable gas-tight sealed vial and is heated at a predetermined temperature for a defined period of time. The volatile components of the sample are distributed between the liquid and gas phase until an equilibrium between both phases is achieved (Penton 2010). A volume aliquot of the gas phase is then injected into a gas chromatograph. All headspace techniques are based on this fundamental principle.

**Fig.1:**
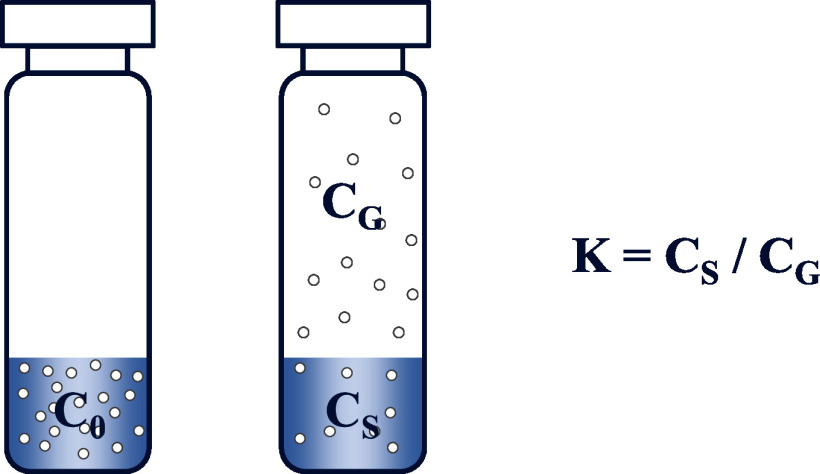
Distribution of a volatile component in a headspace sample vial (c_0_ = original concentration of the analyte in the sample, c_s_ = analyte concentration in the liquid phase after equilibration, c_g_ = analyte concentration in the gas phase after reaching equilibrium state, K = partition coefficient)

Complete equilibrium between both phases is a crucial prerequisite for reliable and reproducible measurements (Sithersingh and Snow 2012). For this reason, the samples are usually subject to thermostatisation for at least 30 min at 40 °C (blood) or 60–80 °C (plasma, urine). After reaching equilibrium, the ratio of the analyte concentration in the sample and the gas phase is constant. This constant is denoted as partition coefficient K (see Figure 1).

The question as to whether headspace analysis can be performed very much depends on the chemical structure of the substance to be analysed, because the partition coefficient K is a substance-specific value. A low partition coefficient means that there is a high analyte concentration in the gas phase compared with the aqueous phase (biological matrix), thereby indicating that the analyte in question is quite suitable for quantification by headspace analysis.

The partition coefficient K depends, among other things, on the solubility of the analyte in the biological matrix. A low level of solubility leads to a higher analyte concentration in the gas phase and thus to a smaller partition coefficient. Various methods, such as salting out or adjustment of the pH value, can be applied to influence solubility (Penton 2010; Sithersingh and Snow 2012).

As the partition coefficient K also decreases with increasing temperature, it is important to ensure a thermostatisation temperature for the headspace analysis that is as high and as constant as possible. For blood samples, however, the thermostatisation temperature has an upper limit in practice, as coagulation sets in at temperatures above 40 °C, making it difficult to reach equilibrium and leading to a higher partition coefficient.

In principle, the concentration of volatile substances in the headspace of a headspace‑vial can be calculated with the formula (equation 1)


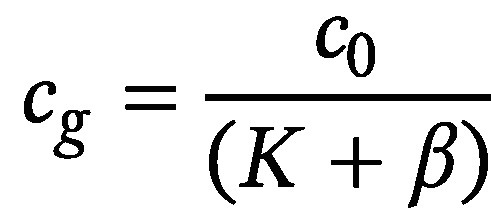
(1)

whereby c_g_ is the concentration of the volatile analyte in the gas phase and c_0_ is the original concentration of the analyte in the sample. The partition coefficient K denotes the equilibrium distribution of the analyte between liquid sample phase and gas phase, and the phase ratio β denotes the volume ratio of gas phase to liquid sample phase.

As the sum of K and β decreases, the concentration of the analyte in the gas phase increases as does the sensitivity of the procedure. An increase in sample volume can contribute to an altered phase ratio β and, in turn, to an increase in sensitivity; in any case, this effect only comes to bear when K is much smaller than β. The partition coefficient K becomes generally smaller with increasing temperatures (thereby corresponding with an increasing concentration in the headspace), whereby this effect is even larger the better the analyte dissolves in the aqueous medium (Kolb and Ettre 2006).

In contrast to the static headspace technique, significantly higher sensitivity can be achieved with dynamic headspace sampling, which is based on multiple extractions of sample aliquots from the gas phase; as a result, even analytes that are only present in very small concentrations can be detected (see Section 2.3).

### 2.2 Static headspace technique with enrichment

Instead of direct injection from the headspace, many static headspace methods use an adsorbent or a cryogenic trap to enrich the analytes from the gas phase prior to transfer into the gas chromatograph. In headspace solid-phase microextraction (HS‑SPME), the adsorbent is inserted directly into the sample vial (Mills and Walker 2000; Pragst 2007). Other enrichment methods include stir-bar sorptive extraction (SBSE) (David and Sandra 2007; Nazyropoulou and Samanidou 2015; Prieto et al. 2010) and single-drop microextraction (SDME) (Jeannot et al. 2010; Palit et al. 2005), both of which are based on a principle similar to SPME. Among those, HS‑SPME is the most widely used technique (Demeestere et al. 2007; Jochmann et al. 2006; Laaks et al. 2012; Nerín et al. 2009).

#### 2.2.1 Solid-phase microextraction (SPME)

**Fig.2:**
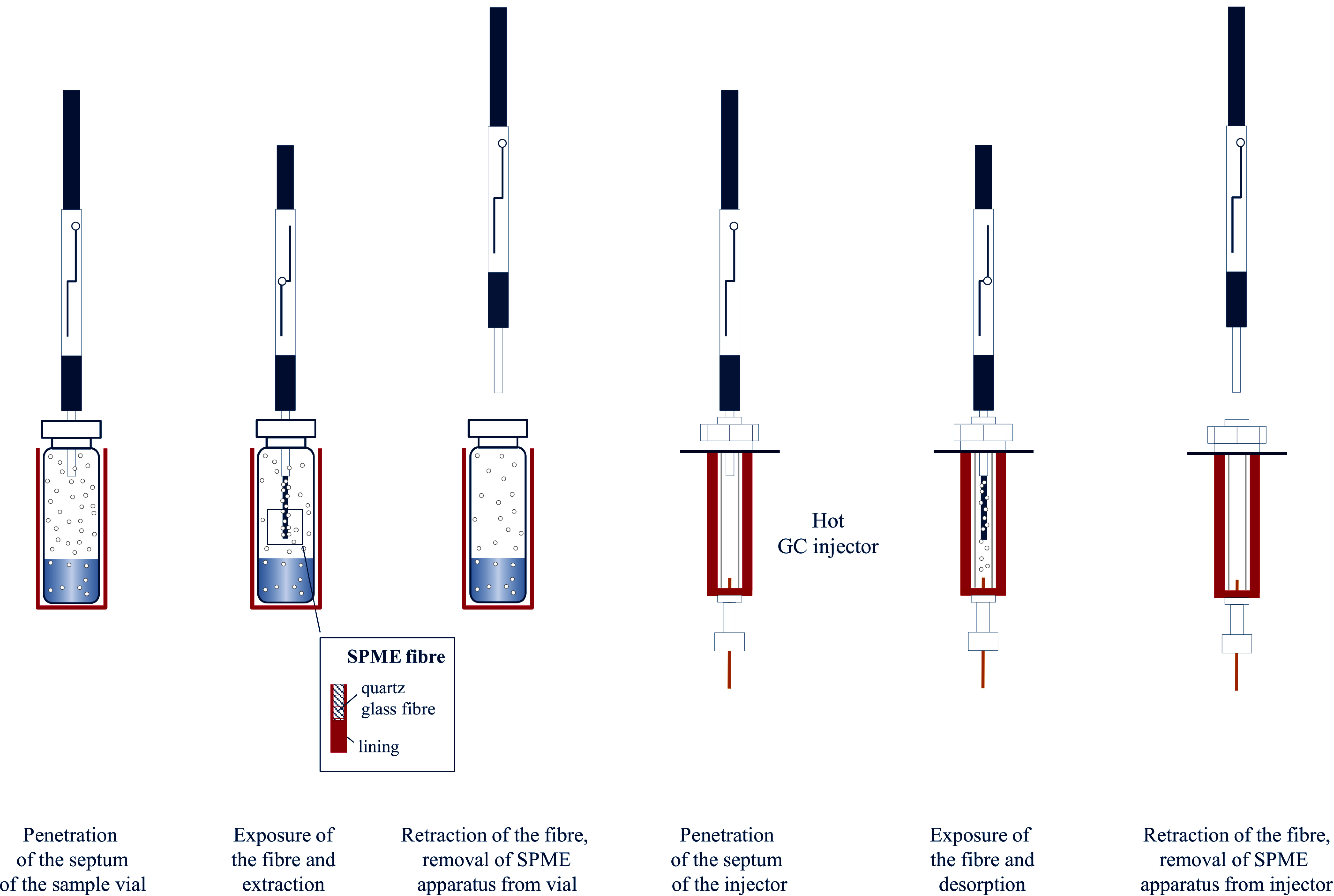
Fundamental procedural steps of the headspace-SPME technique

SPME is a solvent-free extraction technique in which a needle with the dimensions of a typical GC‑injection needle containing a synthetic fibre is inserted into the gas phase of a sample vial via the septum. Afterwards, the SPME fibre is extended into the gas space of the sample vial, rests in this position for a predetermined period of time, and is finally retracted into the needle. The SPME fibre is coated with a stationary phase adapted to the target analytes (e.g. Tenax^®^, silica gel, activated carbon), on which the sorption of the target analytes takes place during this predetermined period (Baltussen et al. 2002; Mills and Walker 2000). In this process, a second equilibrium is achieved in the whole system between the gas phase and the sorbent of the SPME fibre. Compared with the normal, static headspace technique, sensitivity can be considerably improved by targeted influence of the partition coefficients of both equilibria (Sithersingh and Snow 2012). After reaching the sorption equilibrium or after a defined time period has elapsed, the SPME fibre is retracted into the needle and the needle brought into the hot injection port of the gas chromatograph. At this point, the fibre is again extended, and the analytes are released from the sorption phase by thermodesorption and subsequently analysed. Figure 2 shows the fundamental procedural steps of the headspace-SPME technique.

The necessary extraction time is thereby independent of the analyte concentration in the sample (Vas and Vékey 2004). Equilibrium may be expedited by stirring or shaking the sample. Typical SPME fibres can be used for about 100 analyses (Pragst 2007). The special advantages of SPME headspace analysis lie in its relatively simple execution and the comparatively low analytical costs. With SPME analysis, very clean and concentrated sample extracts can be obtained; these extracts are very well-suited for a highly sensitive and selective analysis, such as by mass spectrometry (Nerín et al. 2009; Vas and Vékey 2004).

Since sorption is a competitive and matrix-dependent process, the use of internal standards (ISTDs) may be necessary for quantitative measurements by headspace-SPME. For this purpose, ISTDs are recommended which are as structurally and chemically similar to the target analytes as possible (Pragst 2007). Even with the use of isotope-labelled ISTDs, however, non-linear calibration curves may result (Pragst 2007) (see Section 4.4).

Just like with a static headspace technique, it is essential to maintain consistent analytical conditions (including sample composition, temperature, sample amounts, and headspace volumes) during sample equilibration to guarantee reliable SPME-analysis. Analyte enrichment can be improved by additional cooling of the SPME fibre (Ghiasvand et al. 2016; Pragst 2007).

The main drawbacks of the SPME technique include the mechanical sensitivity of the fibres as well as the limited choice of stationary phases. Moreover, the limited enrichment capacity due to the comparatively small volume of the sorption phase as well as the fibres’ relatively short lifetimes are disadvantageous (Jochmann et al. 2008; Laaks et al. 2010, 2012; Nerín et al. 2009). Newly developed SPME-fibre systems have been optimized accordingly. While conventional SPME fibres have a sorption phase volume of only about 0.6 μl, SPME fibres with larger surfaces provide up to 15 μl of volume available for enrichment. At the same time, certain design alterations (stainless-steel cores for extraction phases, sharpened front end for improved septum piercing) have contributed to the increased mechanical stability of the extraction unit (Kremser et al. 2016).

#### 2.2.2 Stir-bar sorptive extraction (SBSE) / headspace sorptive extraction (HSSE)

In 1999, the stir-bar sorptive extraction (SBSE) technique was introduced to avoid the disadvantages of previously ­developed enrichment techniques. These problems included the low enrichment capacity of the SPME procedure caused by small volumes of sorptive material, among other issues (Baltussen et al. 1999). The SBSE technique was originally developed to concentrate volatile and semi-volatile compounds from aqueous samples. Shortly thereafter, however, headspace applications of this technique began to be published under the name headspace sorptive extraction (HSSE) (Bicchi et al. 2000; Tienpont et al. 2000). In SBSE and HSSE, the analytes are enriched in a comparatively thick sorbent coating which is applied to a glass-sheathed magnetic stirring bar. Depending on the length of the stirring bar, sorbent volumes lie in the range of 25–250 μl. As such, these volumes are two to three orders of magnitude ­higher than the volumes used for SPME analysis. In HSSE, a static headspace enrichment is carried out by introducing the stirring bar into the headspace of a thermostatised sample for a predetermined period of time. Subsequently, the stirring bar is transferred into a thermodesorption system in a glass tube. The thermal release of the analytes from the sorbent material is followed by analysis, e.g. using GC‑MS. Due to the higher sorbent volume, extended desorption times of up to 15 min may arise compared with SPME. Even under these conditions, a quantitative and focused transfer of sample components into the chromatographic system is guaranteed using a cryo-focussing step prior to chromatographic separation (Prieto et al. 2010). The advantages of the SBSE or HSSE technique include automation capability and flexibility with the possibility of enrichment from both the liquid and the gas phases. The high sorbent volume enables a sensitive and simultaneously robust analysis with reproducible results, especially when used in the gas phase and thus bypassing a possible sorption of low volatile sample components (Cordero et al. 2009). For a long time, the selection of available sorption phases was limited to the nonpolar polydimethylsiloxane (PDMS). For this reason, SBSE or HSSE procedures were primarily used for volatile or highly volatile compounds, which also had to be sufficiently thermally stable. In the interim, a PDMS/ethylene glycol copolymer has become commercially available as an enrichment phase in addition to pure PDMS (GERSTEL GmbH & Co. KG 2025). Moreover, numerous other approaches on the development of alternative enrichment phases for SBSE/HSSE have been described in the scientific literature (Nazyropoulou and Samanidou 2015). The historically limited choice of sorption phases, alongside comparatively high costs for the required equipment, have contributed to a rather low prevalence of this procedure compared to SPME, for example (Paiva et al. 2021).

#### 2.2.3 Single-drop microextraction (SDME)

Since the mid-1990s, single-drop microextraction (SDME) has represented a relatively simple and easy-to-implement micromethod for the extraction of target analytes from a matrix or from the headspace above a sample. As part of this method, a droplet of an extraction solvent (hanging on the needle) is formed in the sample vial, usually using a chromatography syringe. The droplet is introduced into the solution to be analysed for a predetermined period of time or, for headspace applications, dwells for this period in the headspace of the sample. Following sorption of the analytes into the solvent, the droplet, which comprises only a few microlitres, is sucked back into the needle of the syringe and subsequently transferred into the GC, where the sample components are separated and subsequently quantified (Afshar Mogaddam et al. 2019; Jeannot et al. 2010).

In headspace‑SDME (Przyjazny and Kokosa 2002; Tankeviciute et al. 2001; Theis et al. 2001), solvents with high boiling points, such as 1‑octanol or long-chain *n‑*alkanes (e.g. *n‑*hexadecane) are generally used for extraction. In principle, however, a comparatively wide variety of sorptive solvents with different polarities can be employed (e.g. *N‑*methylpyrrolidone, ­ethylene glycols, or diethyl phthalate) (Jeannot et al. 2010; Wood et al. 2004). The stability of the drop, which depends strongly on the solvent used, is often a limitation. Here, high volatility, low viscosity, and low surface tension turn out to be unfavourable (Kissoudi and Samanidou 2018). In addition to classic organic solvents, ionic liquids, water, or aqueous solutions can be applied as extraction phases, especially for polar analytes (Afshar Mogaddam et al. 2019; Jeannot et al. 2010; Kissoudi and Samanidou 2018). The procedure of an HS‑SDME analysis is similar to that of an HS‑SPME analysis without the necessity of special additional equipment. Such analyses can therefore be carried out both manually and very well automatised (Wood et al. 2004). Separation and quantitation of the analytes are normally performed by gas chromatography or, more rarely, by liquid chromatography (Jeannot et al. 2010).

### 2.3 Dynamic headspace techniques

#### 2.3.1 Purge-and-trap

**Fig.3:**
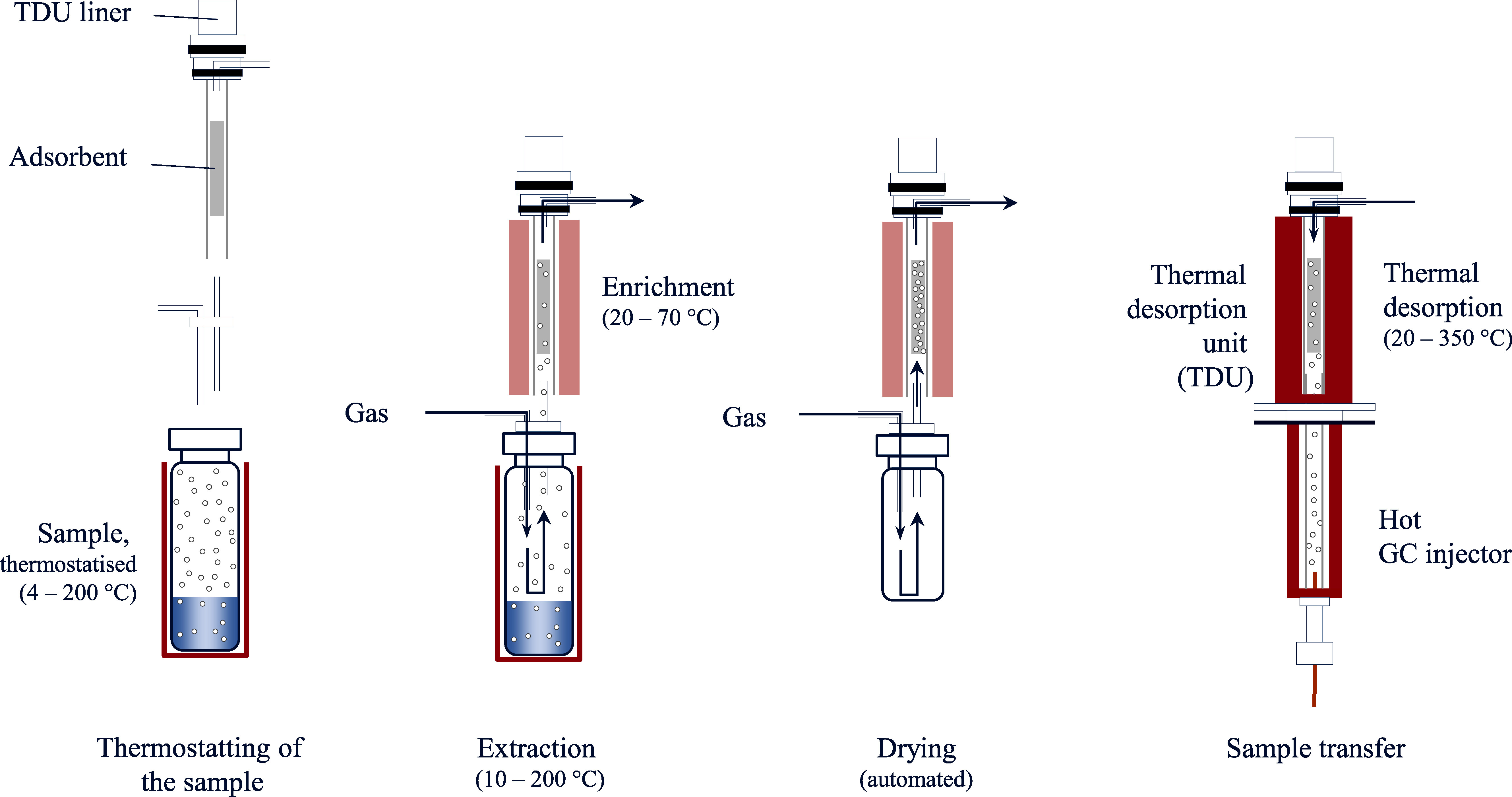
Fundamental procedural steps of the purge-and-trap technique

The purge-and-trap technique is one of the dynamic headspace methods. In this procedure, an inert gas is conducted through an aqueous sample, transporting the volatile analytes into the gas phase. In contrast to static headspace methods, no equilibrium is reached here since the stream of gas continuously purges analytes from the aqueous sample. The volatile analytes are nearly completely transferred into the gas phase by the release of the gas stream from the sample vial and by the continuous flow of inert gas through the sample (Sithersingh and Snow 2012). For analyte enrichment, the gas stream is conducted into a cryogenic trap in which the target analytes are condensed by low temperature and/or locally enriched by sorption (adsorption on a surface, absorption in a liquid phase). After completing the extraction step, desorption of the analytes is carried out analogously to the SPME technique by thermo­desorption in the GC injector (Figure 3).

By the continuous extraction of the volatile analytes from the matrix, this technique enables considerably lower detection limits compared to static headspace analysis. If a sorbent trap is used, the wide variety of sorption materials presents yet another advantage. In multi-analyte methods, for example, multi-layer sorbents may be applied (e.g. made of Tenax^®^, silica gel, activated carbon) which are able to bind a broad spectrum of analytes (Sithersingh and Snow 2012).

The risk of contamination is one disadvantage of this technique. As the inert gas bubbles through the aqueous ­sample, the gas stream contains small amounts of water as it leaves the system, which may interfere with the subsequent ­analysis. This problem is partially addressed with downstream drying steps (Figure 3). As the analytes must additionally cover a rather long distance to the injector, the risks of contamination, of adsorption or of condensation on cooler surfaces as well as of peak broadening in the subsequent chromatography are generally increased. Due to ­potential formation of foam by the inert-gas stream, this technique is only partially applicable for biological materials, particularly blood. Alternatively, the gas stream can be conducted along the surface of the sample (Demeestere et al. 2007), which reduces the enrichment rate but also leads to analyte extracts that are low in water vapour. Compared to other methods, the time required for the purge-and-trap technique is relatively high (Demeestere et al. 2007).

#### 2.3.2 In-tube extraction (ITEX)

The in-tube extraction (ITEX) technique is a relatively new, solvent-free enrichment method. In this process, enrichment takes place directly in the headspace syringe, whereby the solid adsorption material (usually Tenax TA) is embedded in the upper part of the needle. The analyte trap can be flash heated, which guarantees an optimal thermo­desorption of the analytes into the GC injector.

**Fig.4:**
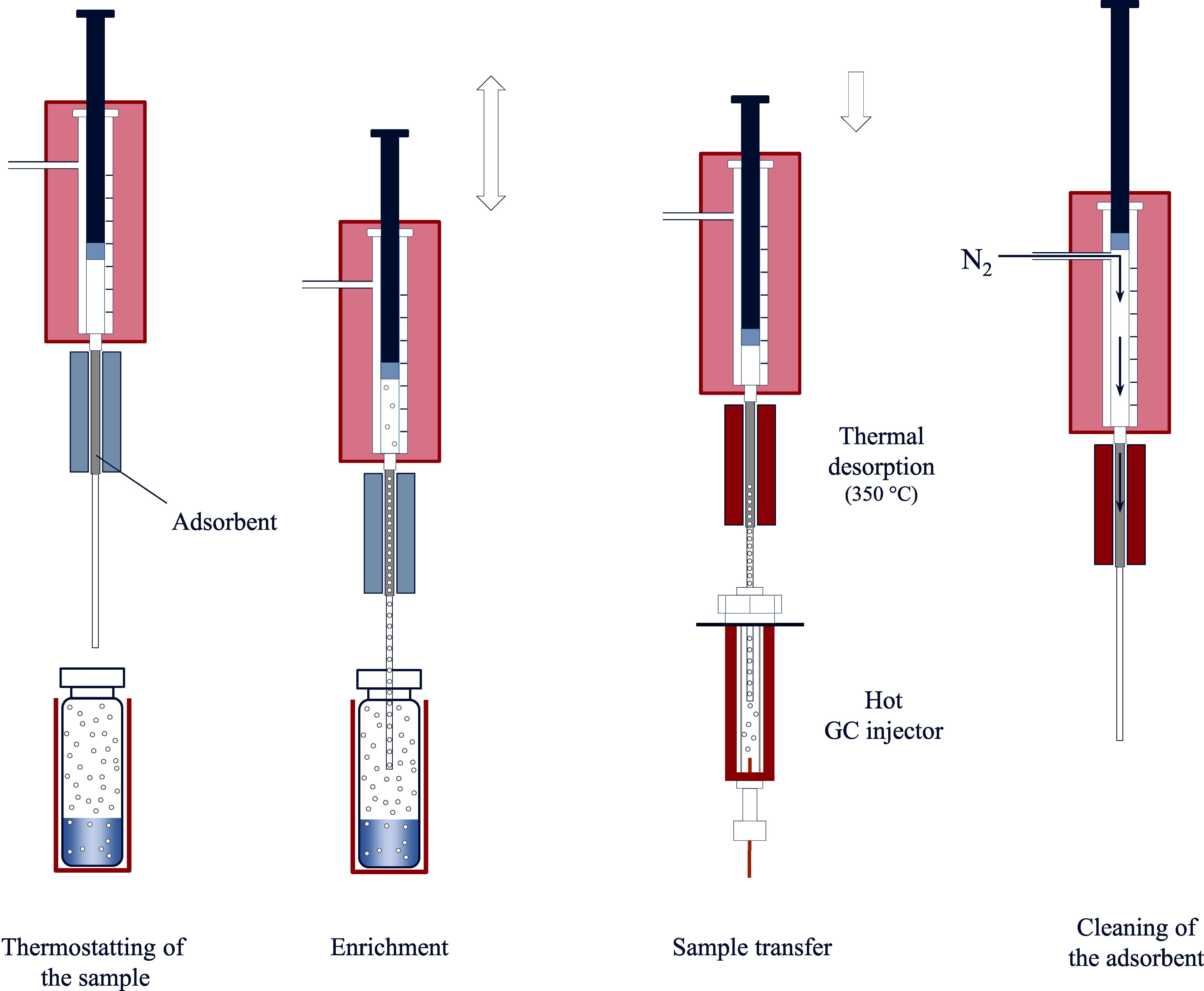
Fundamental procedural steps of the ITEX technique

Just like with other headspace techniques, the sample to be analysed is first thermostatised under defined conditions and stirred or shaken as needed. The needle then pierces the septum of the sample vial and the gas phase is drawn into the needle multiple times, whereby the analyte is conducted over the adsorption material and retained there. The needle is then introduced into the GC injector and the analyte is directly analysed following thermal desorption. After desorption, the adsorption material is cleaned by flushing the hot needle with an inert gas. Figure 4 shows the basic procedural steps of the ITEX technique.

The advantage of the ITEX technique is that sample preparation and enrichment take place in one step, meaning that this process can be completely automated. Moreover, this procedure has a considerably decreased risk of contamination (Laaks et al. 2010).

The main advantages of the ITEX technique, compared with SPME, are its considerably higher adsorption capacity, increased mechanical stability, and faster analyte enrichment by active drawing of the gas phase (Jochmann et al. 2008; Laaks et al. 2010; Nerín et al. 2009). Furthermore, the ITEX syringe exhibits a longer lifetime and can be used for up to 1000 extractions. The trap heater allows for heating the needle for thermodesorption independently of the GC injector temperature (Jochmann et al. 2008; Rasanen et al. 2010). As such, considerably lower detection limits can be achieved with this technique and a multitude of analytes can also be detected below the concentration range relevant for occupational medicine (Laaks et al. 2015; Rasanen et al. 2010). A particular advantage over both the SPME (see above) and the SPDE (see below) technique is the versatility of the ITEX technique: the trap contains packed sorbent material, which can be selected from a larger number of materials (Laaks et al. 2012).

In addition to the contributing factors observed in static headspace analysis, enrichment with this technique is significantly influenced by both the selection of the adsorbent as well as by the number of strokes (and, in turn, the number of extraction cycles) (Laaks et al. 2010, 2015). Analogously to the SPME technique, analyte enrichment can be improved by cooling the needle (Laaks et al. 2015).

#### 2.3.3 Solid-phase dynamic extraction (SPDE)

The principle of the solid-phase dynamic extraction (SPDE) technique is predominantly analogous to ITEX enrichment and was developed as an improvement to the SPME technique (Lipinski 2000, 2001).

**Fig.5:**
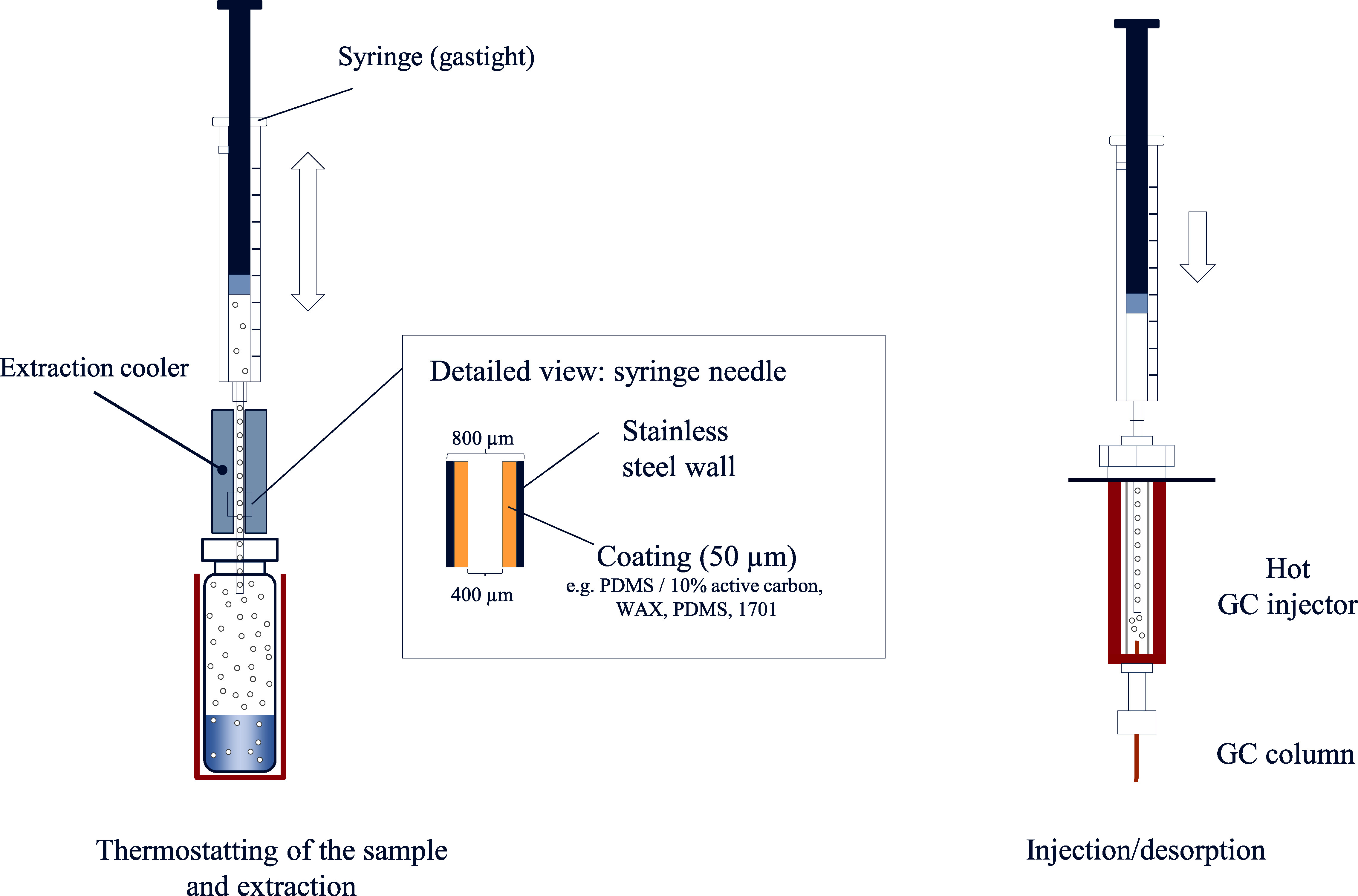
Operating principle of the SPDE technique

In contrast to the ITEX method, however, the sorption material is not embedded in the syringe-needle but is rather coated onto the inner wall of the needle. As in the previous approach, the needle is inserted through the septum into the sample vial and extraction is carried out dynamically by drawing up the syringe multiple times. The analyte is again released into the GC injector by thermodesorption and subsequently analysed (Nerín et al. 2009) (Figure 5). In the SPDE technique, analyte enrichment can be improved by cooling the needle as well (Jochmann et al. 2006).

The advantages of this technique correspond with those of the ITEX technique (see Section 2.3.2) and lie primarily in the improved detection sensitivity, whereby this enrichment technique is also suitable for the detection of trace amounts of polar volatile substances (Jochmann et al. 2006). The possibility to adjust the extraction efficiency by modify­ing the number of strokes (Nerín et al. 2009) as well as the procedure’s general suitability for automatisation (Laaks et al. 2012) are similarly advantageous. The relatively small selection of stationary phases for analyte enrichment is a drawback of this technique (Laaks et al. 2012).

## 3 Headspace analysis in human biomonitoring

### 3.1 Biological materials

An important prerequisite for human biomonitoring is the appropriate collection and workup of a suitable biological material in which – in the case of exposure monitoring – the concentration of the hazardous substance or the respective metabolite reflects the total exposure of an organism. At present, blood, plasma, serum, erythrocytes, and urine are generally preferred for the quantitative determination of exposure to hazardous substances in the context of occupational medicine; in most cases, there is a strong correlation between workplace exposure and the respective biomarker concentrations when using these matrices. Another advantage of blood and urine as sample materials is that there are standardised sampling procedures and that they are easily accessible under routine conditions for occupational or environmental medical issues: collecting these sample materials is tolerable for the persons concerned, and the material is available in sufficient amounts (Alves et al. 2014; Angerer et al. 2007).

Correspondingly, the headspace methods for human biomonitoring by the Commission published to date have been developed and validated for the matrices blood and urine (see Section 5.1). Whether a parameter is determined in blood or urine depends on absorption and excretion kinetics as well as on the metabolism of the hazardous substance in question. Furthermore, potential contamination must be considered; this problem arises predominantly when quantifying unmetabolised hazardous substances (see Section 4.1). Moreover, in the field of occupational medicine, the assessment values for human biomonitoring (e.g. biological tolerance values (BAT), biological guidance values (BLW), biological limit values (BLV), and biological exposure indices (BEIs)) are almost exclusively derived for blood and urine (ACGIH 2025; DFG 2025; RAC 2025).

In the scientific literature, further biological matrices have been described which may be used to quantify volatile substances by headspace‑GC. However, interest is then mostly not focussed on the field of occupational medicine, but rather on questions in the fields of environmental medicine, forensic medicine, or toxicokinetics. Alongside blood and urine, these fields of work investigate breast milk, faeces, saliva, cerebrospinal fluid, homogenised tissues, and other biological matrices (Mills and Walker 2000; Seto 1994).

### 3.2 Analytes and substance groups

In human biomonitoring, headspace analysis was originally applied to determine volatile organic compounds (VOCs) in relatively high concentrations. According to the European Council Directive 1999/13/EC, VOCs can be defined, with regard to their physicochemical properties, as substances which possess a vapour pressure of at least 10 Pa at 20 °C (European Council 1999). A wide spectrum of substances falls under this definition, which was also adopted from the International Union of Pure and Applied Chemistry (IUPAC) (Duffus et al. 2007), including aliphatic and aromatic hydrocarbons as well as oxygenic, nitrogenous, sulphurous, and halogenated compounds (Hunter and Oyama 2000).

In addition to these substances, which are volatile due to their intrinsic physicochemical properties, headspace ­ana­lysis is generally also possible for compounds which can be transformed into volatile substances by derivatisation, chemical or thermal conversion, or another form of sample preparation. Such examples include the derivatisation of trifluoroacetic acid (Dallmeier and Müller 1982), the protein-adduct cleavage of aldehydes in serum (Silva et al. 2018), the thermal conversion of *N‑*hydroxymethyl-*N‑*methylformamide (HNMF) to *N‑*methylformamide (Fernandes Knupp et al. 2005), as well as the thermal decomposition of tri­chloro­acetic acid into chloroform (Angerer and Eben 1980) or of formic acid into carbon monoxide (Angerer and Schaller 1980).

The highly sensitive analytical technology available today enables the detection of substances down to ultra-trace levels (Imbriani and Ghittori 2005), whereby less-volatile substances can be detected as well as substances which are only present in low concentrations (Fantuzzi et al. 2001; Imbriani and Ghittori 2005; Takeuchi et al. 2002). There is no strict definition for the range of ultra-trace analysis, it is mostly used in the literature for mass fractions of less than 10^–6^ to 10^–8^ g/g (1 ppm to 10 ppb) (Brown and Milton 2005). Accordingly, in recent years, the Commission has published headspace methods on the determination of unmetabolised aromatic compounds (Van Pul et al. 2018) and halogenated hydrocarbons (Roßbach et al. 2019) in urine, both of which are excreted in the urine only in small amounts.

For the effective measurement and monitoring of workplace exposure via human biomonitoring, methods for individual volatile compounds or multi-analyte methods in which the analytes possess structural similarities were ­developed. For example, the Commission developed and published methods for the combined analysis of BTEX aromatic compounds in blood (Angerer et al. 1994; Knecht and Angerer 1983) or, more generally, for the measurement of aromatic compounds in blood or urine (Göen et al. 2018; Van Pul et al. 2018). Other methods encompass the determination of alcohols, ketones, and ethers in urine (Angerer et al. 1997; Göen et al. 2020) or of halogenated hydrocarbons in blood (Angerer et al. 1991; Göen et al. 2021) or in urine (Roßbach et al. 2019).

The fact that analytical procedures were initially developed for the determination of non‑polar hydrocarbons in blood or blood compartments and for the determination of polar hydrocarbons in urine has accounted not only for the solubility behaviours in the individual biological matrices, but also for physiological processes, because polar substances or polar metabolites are primarily excreted with the urine. Consequently, the assessment values (see Section 5.2) for these parameters were initially only derived for the corresponding matrices.

The half-lives of the respective substances in blood or urine also influence the selection of the matrix for the deter­mi­na­tion of individual biomonitoring parameters. Highly volatile substances present in the blood are primarily exhaled via the lungs, meaning that they are eliminated very rapidly after exposure (see Table 1). The Commission established a sampling time of “immediately after exposure” for these parameters in the List of MAK and BAT Values (DFG 2025). This sampling time currently applies to the occupational-medical biomonitoring of 1,2‑dichlorobenzene, di­chloro­methane, and toluene in blood. In any case, the correct timing for sample collection of hazardous ­substances with short half-lives represents a major challenge in the practice of occupational medicine. For this reason, the Commission has withdrawn the assessment values for benzene, toluene, and xylene isomers in blood and derived new assessment values in urine (DFG 2025).

Substances which are excreted with the urine usually have longer half-lives than volatile hazardous substances in the blood (see Table 1); this observation is especially true for the metabolites of hazardous substances, but also for some unmetabolised hazardous substances in the urine.

### 3.3 Detectors

Various detectors are used in combination with headspace-gas chromatography (Angerer and Schaller 1976). In the early days of headspace-gas chromatography, flame ionisation detectors (FIDs) and electron capture detectors (ECDs) were mainly used. The FID is a very universal detector which sensitively measures carbon-containing compounds and exhibits a broad, linear working range over six orders of magnitude. The ECD is considered a selective detector, as it predominantly indicates compounds with high electron affinities. Especially halogenated and nitrated substances are sensitively measured, whereas other nitrogen- and oxygen-containing compounds are measured with lower sensitivity. Regarding the detection limits for these analytes, ECD outperforms FID by several orders of magnitude.

While FID and ECD have been replaced by mass-spectrometric detectors in modern analysis, their continued use is arguably justifiable, especially in headspace analysis, as the samples in question exhibit a rather low matrix burden. Furthermore, both detectors are ready for operation very quickly and do not require long equilibration times after changing the column.

In recent years, mostly headspace methods with mass-spectrometric detection have been developed, applied, and published. However, the MS detector can only exhibit its strengths to a limited extent, as the rather small molecules measurable with headspace techniques often form unspecific fragments. For the same reason, the use of tandem‑MS techniques to increase sensitivity and/or selectivity is generally not effective or not necessary due to the low background noise. An important advantage of mass-spectrometric detection is that isotope-labelled ISTDs can be used. Another argument for the MS detector is that it can be used in a more versatile way, allowing for example for the detection of both pure hydrocarbons and low-carbon-containing substituted compounds.

## 4 Practical aspects and sources of error

Due to special conditions for sample collection and due to the investigation of metabolically unchanged biomarkers, the quality of headspace analyses depends largely on influencing factors and sources of error in the pre-analytical phase (see below). Influencing factors are defined as changes of analyte levels in vivo, meaning before actual specimen collection (e.g. by sampling time, smoking tobacco, alcohol consumption, medications, drug abuse). On the other hand, sources of error are defined as changes of analyte concentrations which take place during or after specimen collection, e.g. due to contaminations or changes of the sample matrix during transport and storage (Bader et al. 2010). Sources of error, in particular, are relatively easy to identify and can be controlled or minimised by the provision of appropriate specifications in the standard operating procedures.

### 4.1 Pre-analytical phase

The so-called “pre-analytical phase” consists of sample collection as well as the transport and storage of human bio­logical material prior to the actual analysis. These steps must ensure that contamination or loss of analytes is avoided in order to achieve correct and reproducible results. Inadequate procedures in the pre-analytical phase may lead to significant contaminations or analyte losses; as these issues cannot be estimated either analytically or mathematically, they cannot be corrected.

#### 4.1.1 Containers and materials

When performing headspace methods, laboratories must ensure that all equipment and chemicals used are clean and free of contamination. Glassware used for the preparation of standards as well as headspace vials, including septa and caps, should be baked out (several days at about 200 °C, e.g. in a drying cabinet) and used immediately if possible or stored separately for only a short time and safe from contamination. When baking out, it should be noted that the septa are only stable up to a certain temperature (80–210 °C) depending on the material. Piercing the septum with a heated needle can also lead to temperature-dependent leaks in multiple measurements from the same headspace vial (Kolb and Ettre 2006).

#### 4.1.2 Sampling time

In general, specimen collection must take place at a time in which the analyte concentration of the biological material to be analysed is in equilibrium with external exposure. For the determination of volatile organic compounds (e.g. aromatic hydrocarbons in blood), the biological material must be collected at the end of exposure or, for longer lasting work activities, at the end of the shift. The half-lives of unmetabolised solvents in blood vary between 30 min and a few hours (see Table 1). If a hazardous substance is listed in the List of MAK and BAT Values or similar guidelines, sampling should take place at the time specified in the guideline (DFG 2025).

#### 4.1.3 Specimen collection

For headspace analysis, specimen collection requires the use of supplies (sample vials, sampling equipment, dis­in­fectants) which are free of contaminants and, in some cases, pre-treated in a certain way. Sampling recommendations as described in the standard operating procedures for headspace methods published by the Commission (see Tables 2, 3 and 4) can be summarised as follows:

If volatile substances are to be determined in blood or urine, it is important to protect the collected sample from ­analyte loss until analysis. This may be achieved, for example, by transferring the sample material into baked-out (and thereby contaminant-free), gas-tight sealed “perforable ampoules”/headspace vials directly after specimen collection. The headspace vials serve both as storage and transport containers and are generally provided by the laboratory. Empty headspace vials should be stored for as short a period as possible, and if necessary, only under storage conditions which are as constant and contamination-free as possible.

Collection equipment consisting of disposable syringes and cannulae are used for blood extraction, whereby venous blood samples with added anticoagulant (e.g. EDTA, heparin) are required for headspace analysis. A diluted hydrogen peroxide solution (approx. 3%) should be used to disinfect the cubital fossa, because the contents of commonly used disinfectants, as well as impurities taken up by the disinfectant during storage, may be a potential source of contamination. The blood sample taken from the arm vein is thoroughly mixed immediately after venepucture in order to evenly distribute the anticoagulant. A defined aliquot (usually one to two millilitres) is transferred into the headspace vial. The venepuncture instruments should also be stored as briefly as possible under contamination-free conditions.

For urine collection, disposable plastic containers (urine cups) are used. Urine cups are commercially available and usually hold 100 ml of liquid. The urine sample is collected directly in the container at the prescribed sampling time, whereby it is important to avoid contamination, especially by dusts, but also by gases or vapours in the workplace. For the determination of volatile organic substances in urine, a disposable syringe is used to transfer a defined aliquot (usually one to two millilitres) of the fresh, spontaneous urine sample into a baked-out headspace vial.

#### 4.1.4 Sample transport, storage, and stability

As soon as possible after specimen collection, blood and urine samples should be transferred into gas-tight sample vials and sent to the test laboratory. Under certain circumstances, depending on the parameters to be determined, blood and urine samples may also be shipped in fully filled sample vials with minimal headspace. This approach limits preliminary distribution and counteracts analyte loss. It is important to ensure that samples are kept safe from contamination during transport. Human samples that are only minimally likely to contain pathogens may be shipped as ‘exempted medical samples’ without specifying a UN number (“P 650 light”) (Bundesregierung Deutschland 2021). For this purpose, the sample must be in triple packaging, consisting of a watertight primary container, a watertight secondary packaging and a sufficiently strong outer packaging. For liquid substances, a sufficient amount of absorbent material between the primary container and secondary packaging must be ensured. In addition, the parcel must be labelled “exempt human specimen”.

If it is not possible to ship samples directly after specimen collection, samples for headspace analysis may be stored for a few days under the storage conditions given below. The refrigerator and freezer units used for storage may not be located in laboratories in which solvents are handled. Moreover, materials which contain or may release solvents should not be stored in the same place as headspace samples. For many analytes, it is generally possible to store blood and urine samples cooled over a period of several days without analyte loss (Ashley et al. 1996; Gill et al. 1988). Ogawa and Sasahara (2012) investigated the storage stability of toluene in blood samples and found that short-term (up to three days), cooled storage of blood samples did not lead to any significant losses. In another study, which investigated di­chloro­methane in urine, no significant differences could be ascertained between storage at room temperature or in the refrigerator (Hoffer et al. 2005). In any case, it is important to quickly transfer collected samples into gas-tight sample vials (Hoffer et al. 2005; Ogawa and Sasahara 2012).

For certain analytes, it may be additionally important to store the samples in the dark. In-house investigations have shown that the storage stability of halogenated hydrocarbons, especially carbon tetrachloride, was higher when the samples were stored in the dark (see Appendix).

### 4.2 Sample preparation

In headspace analysis, the aim of sample preparation is to make analytes accessible to determination, to increase analyte concentrations in the headspace over the sample, or to improve method precision by adding an ISTD.

#### 4.2.1 Conversion of analytes into volatile compounds

Compared to other extraction and analytical procedures, headspace analysis possesses considerable advantages (simple sample preparation, efficient separation of the analytes from the biological matrix, low chromatographic background noise), such that it is also advantageously applied for substances which are not volatile but can be converted into volatile compounds by suitable measures.

This principle holds, for example, for the determination of the carbon monoxide‑haemoglobin (Hb) content in blood, which is based on the release of carbon monoxide and subsequent catalytic conversion into methane (Angerer and Zorn 1985). Even tri­chloro­acetic acid (the metabolic product of tri­chloro­ethene, tetra­chloro­ethene, 1,1,1‑tri­chloro­ethane, and other aliphatic chlorinated hydrocarbons), which is not volatile, can be determined by headspace-GC analysis following thermal decarboxylation. The chloroform formed in this reaction can be measured very sensitively and specifically (Christensen et al. 1988, Will et al. 2017). Trifluoroacetic acid, the metabolite of halothane, can be quantified using the headspace technique after direct esterification with tri­chloro­ethanol in the headspace vial (Dallmeier and Müller 1982). Finally, analytes may also be released via the addition of acid, such as in the conversion of cyanide into hydrocyanic acid (Eben and Lewalter 1988).

With respect to conversion into volatile compounds, it is important to note that every procedural step and every addi­tion of chemicals may lead to analyte loss or sample contamination.

#### 4.2.2 Increase of the analyte concentration in the headspace

The analyte concentration in the headspace above a sample depends mostly on the concentration of the substance in the sample material, the value of the partition coefficient K, and the phase ratio in the headspace vial (see Section 2.1). The partition coefficient K can generally be influenced by the addition of a salt (“salting out”) or by adjustment of the pH value. Moreover, a change in temperature can increase or accelerate the enrichment of the analyte in the headspace.

The salting-out reduces the solubility of the analyte in the aqueous phase, thereby increasing the analyte concentration in the headspace (Grover and Ryall 2005). Ammonium chloride, ammonium sulfate, sodium chloride, sodium sulfate, and potassium carbonate are most commonly used for this purpose (Kolb and Ettre 2006). Adding these salts most likely reduces the solubility of polar VOCs in aqueous sample matrix, while non‑polar substances with a low K value are barely affected at all (Kolb and Ettre 2006). For a maximum “salting-out” effect, it is important to reach the saturation concentration in order to avoid differences in concentration and, in turn, varying phase equilibria in different samples. However, salt often contains volatile impurities and high salt concentrations lead to an increased viscosity of the aqueous phase, making a longer thermostatisation time necessary (Kolb and Ettre 2006). As salting out is not generally advantageous, this approach must be tested for the individual analytes.

Adjustment of the pH value of a sample may also contribute to the maximisation of the analyte concentration in the gas phase by reducing the solubility of the analyte in the aqueous phase. For example, volatile acids are protonated by a reduced pH value and thereby become less soluble; for amines, deprotonation and therefore decreased solubility can be reached by increasing the pH value. The addition of strong acids and bases is not recommended for the blood matrix as this triggers coagulation.

The addition of acids or bases may considerably alter the release of analytes from biological materials. Smith et al. (2008) could significantly increase the concentration of acetaldehyde, ethanol, furan, hexanal, 2‑methylfuran, 3‑methyl­furan, octanal, phenol, propanal, and toluene, in the vapour phase, particularly by acidifying urine samples. This investigation did not ascertain to which extent decomposition reactions were responsible for the increase in analyte releases (Smith et al. 2008).

Regarding the addition of chemicals (salts, acids, etc.), it must be noted that every procedural step after sample collection and subsequent transfer of a sample aliquot into a gas-tight headspace vial increases the risk of analyte loss or sample contamination.

### 4.3 Sources of error

#### 4.3.1 Blank values, contamination, analyte loss

Blank values involve impurities with the respective analytes which originate from any equipment and chemicals used. Ashley et al (1996) showed that blood sampling using untreated Vacutainers^® ^led to significantly higher blood levels of *n‑*bromoform and *m‑*/*p‑*xylene, whereas this was not observed for 1,4‑dichlorobenzene. Decontamination of the collection tubes by appropriate pretreatment of the Vacutainers^®^ (Ashley et al. 1992) was therefore necessary for the VOCs concerned. Moreover, ethylbenzene and xylene blanks of 11–14 μg/l and 51–65 μg/l, respectively, were ­detected when comparing various sampling tubes for BTEX analyses. Using baked-out septa, these blank values could be reduced significantly (Bader et al. 1994). In in-house studies, benzene blank values of up to 5 μg/l were detected when comparing various Vacutainer^®^ types. Using specially prepared Vacutainer^®^ plugs, this blank value could be reduced to the ­lower level of an alternative venepuncture kit (Monovette^®^) (see Appendix). Moreover, the test material may be externally contaminated with the target analytes which may originate from sample collection or sample preparation (Heinrich-Ramm et al. 2004).

Kolb and Ettre (2006) emphasise that blank values often arise from the septa used, that contaminations emerge from the water used for blank-value measurements, or that contaminations from laboratory air may pollute the sample. Moreover, Kolb and Ettre (2006) note that especially the purge-and-trap technique may lead to memory effects. In this enrichment technique, carryover of sample components may be caused by aerosol formation due to the bubbling of gas through the sample.

Losses may occur from the evaporation of analytes from the sample, via adsorption of the analytes to material surfaces, or by chemical reactions in the sample itself. For some substances, microbial degradation may occur if storage conditions are not selected appropriately. Another significant, easily avoidable cause of analyte loss is evaporation due to untight or insufficiently sealed headspace vials (Kolb and Ettre 2006): it should be either impossible or very difficult to turn the crimp caps of the sample vials.

In-house investigations have shown that it is often possible to easily turn the aluminium crimp caps of headspace vials after one-day storage in cooled (4 °C) and especially in frozen (−20 °C) conditions (see Appendix). Especially after sample collection at room temperature and subsequent storage of the sample vials at low temperatures, the different expansion coefficients of the individual components of headspace vials (glass, aluminium, rubber, silicone) may lead to leakages. This effect may lead to both external contamination as well as analyte loss, and should be checked promptly after the desired storage temperature has been reached and, if necessary, avoided by newly crimping or retightening the loose crimp or screw cap.

Regarding microbial degradation, our own investigations indicate that the addition of sodium chloride (1 g/ml ­sample) may inhibit fungal growth for example in urine samples stored at room temperature. For instance, methanol degradation was determined in urine samples that had not been stabilised with sodium chloride; this loss was not observed in samples to which sodium chloride had been added (see Appendix).

Special applications, such as the use of sample tubes with negative pressure (e.g. Vacutainer^®^) for aliquoting and ­storing urine samples, may reduce the risk of both contamination and analyte loss (Kawai et al. 2011).

#### 4.3.2 Changes in distribution equilibria

According to the Henry-Dalton law, an increase in incubation temperature also leads to changes in phase equi­librium, as the partial pressure of the analyte increases (desired effect) as well as the partial pressure of water from the bio­logical matrix (undesired effect). Even if, in a best-case scenario, the concentration of the analyte in the gas phase increases more than the concentration of water, an increased entry of water vapour/water onto the chromatographic separation column or into the detection system is generally disadvantageous for the stability/reproducibility of the analysis as well as for the service life of the headspace‑GC system.

When using blood as matrix in headspace methods, it is generally important to avoid coagulation of the blood, which takes place particularly at high temperatures. If an anticoagulant (EDTA, citrate, etc.) has been added to the blood sample, it can be heated up to 50 °C for headspace injection. For samples without anticoagulant, coagulation already occurs at temperatures above 40 °C, whereby a reliable establishment of the distribution equilibrium can no longer be guaranteed.

### 4.4 Calibration and control materials

#### 4.4.1 Calibration

The quality of headspace analysis with respect to precision, reproducibility, and robustness is determined largely by the adjustment and maintenance of constant conditions (temperature and pressure control, ratio of liquid to gaseous phase, equilibration time, etc.). The operational parameters of sample equilibration lead to a distribution equi­librium which directly influences the amount of transferable and thereby quantitatively measurable target analyte. Compared to the simple injection of liquid extracts or gas volumes, calibration in a state of phase equilibrium places certain challenges on the stability of the analytical system used as well as on the calibration standards and their preparation: to ensure reproducible and correct results, it is necessary, for each analytical method, to establish a calibration procedure which best reflects the concentrations and distribution ratios of the sample to be analysed and, in turn, can be directly used for evaluation or at least enables the derivation of a correction factor (Kolb and Ettre 2006). In general, the calibration material is prepared using the respective biological matrix (blood, plasma/serum, urine) which corresponds with the sample material and which therefore also accounts for any effects from storage, workup, as well as for the distribution equilibrium between sample matrix and headspace.

While calibration in urine can be carried out using pooled individual urines from non-occupationally exposed individuals, calibration in whole blood is more complex: aside from the process of reaching equilibrium between the aqueous biological matrix and the gas phase, distribution processes also take place between the cellular components of the sample (e.g. lipid membranes), free macromolecules, and agglomerates (e.g. proteins, lipoproteins) and the plasma. For this reason, it is important to consider that the equilibrium concentrations between the matrix components of samples collected in vivo differ from those of a calibration sample freshly prepared in the same matrix. Additional changes and differences may arise when whole-blood samples are stored frozen prior to analysis, as the composition and physico­chemical properties of the matrix are altered by the lysis of the erythrocytes. In this context, differences between species must be noted as well: due to differences in quantitative and qualitative blood composition (e.g. haematocrit, serum/plasma proteins, lipids), the suitability of animal blood for the calibration of hazardous substances in human blood must be verified on a case-by-case basis. Aside from availability and cost, potential background concentrations of the target parameters, which are often higher in human blood than in blood from other species, must be considered when deciding whether to use either animal or human blood as a calibration matrix (Heinrich-Ramm et al. 2004). In addition, some blood‑gas partition coefficients, such as for desflurane, sevoflurane, isoflurane and methoxyflurane in the blood of nine common animal species, differ from those in human blood, which may be due to species‑related differences in triglyceride concentration and binding to haemoglobin, plasma proteins and erythrocyte membranes (Soares et al. 2012).

In a paper, Heinrich-Ramm et al. (2004) compared various established calibration methods for the headspace analysis of aromatic compounds in blood within an interlaboratory comparison. To this end, an ethanolic starting solution of benzene, toluene, ethylbenzene, *m‑*xylene, and *o‑*xylene (20 000 mg/l) was first diluted with ethanol to give stock solutions with concentrations between 100 mg/l and 800 mg/l and subsequently diluted to concentration ranges relevant for occupational medicine (≈ 5–500 μg/l). The dilution steps were carried out with whole blood (defibrinated horse blood, native human blood) or a physiological saline solution. Gas-chromatographic static-headspace analysis was then performed using the analytical instruments available in each laboratory. The influence of the individual instrumentation was additionally investigated by exchanging differently prepared calibration standards. The study showed that the origin of the whole blood used (horse, human) leads to significant differences in the slopes of the calibration functions, and that the more laborious dilution in volumetric flasks, similar to diluting exclusively with physiological saline solutions, leads to flatter calibration curves as well as to an overdetermination when compared to purely volumetric dilution with whole blood in headspace vials (pipetting calculated volumes instead of using graded volumetric flasks). The main outcome of these comprehensive investigations was the recommendation to perform combined dilution, first with a physiological saline solution, then with whole blood and to favour pipetting with previously calculated volumes over the use of volumetric flasks. This procedure yielded quite consistent results with the target values of the 24^th^ interlaboratory trial of the G‑EQUAS (German Quality Assessment Scheme, https://app.g-equas.de) (Heinrich-Ramm et al. 2004).

The work of Heinrich-Ramm et al. (2004) proves the strong dependence of headspace analytical results on matrix effects, especially with regard to the preparation of calibration standards and the matrix used for this purpose. It is expected that these effects are less pronounced in less complex matrices (serum/plasma, urine). Even so, it is important to ensure that calibration standards are prepared in an efficient, timely manner in order to minimise any analyte losses during the process.

In cases of sufficiently high analyte concentrations, one possibility for dealing with matrix problems is to simply ­dilute the measurement solution. There are, for example, guidelines for the determination of the blood ethanol content which recommend a 1 ∶ 10 dilution of blood samples with an aqueous medium (Kolb and Ettre 2006). The possibility of using dilution to minimise matrix effects in whole-blood samples has also been analysed by Alonso et al. (2013), who investigated twelve VOCs by SPME‑HS‑GC‑MS. The authors describe that the influence of the blood matrix on the recovery of the analytes depends on their boiling points. A 1 ∶ 5 dilution with water improved recovery and enabled quantitative extraction of most analytes. However, in the case of 1,2‑dichlorobenzene, which has a boiling point of 180.5 °C, the matrix effect could not be compensated for by a mere 1 ∶ 5 dilution with water (25% recovery).

When using only physiological saline solution, analyte losses also indicate that a procedure which is as simple and quick as possible is advisable with regard to the preparation of calibration standards (Heinrich-Ramm et al. 2004). For headspace analysis, Kolb and Ettre (2006) recommend the consistently fresh preparation of calibration standards from stock solutions. In the case of multi-substance standards, it is advisable to add the analytes to the matrix by order of volatility, starting with the least volatile substance. This approach is most important for highly volatile substances with low partition coefficients. For storage, the stock solutions are filled into well-sealed threaded glass vials, which should be filled as full as possible.

When preparing the stock solutions, depending on the analytes and the matrix, a solvent is first placed in the glass vial into which the volatile analytes are then weighed. As an alternative to weighing out pipetted volumes, microlitre syringes – exhibiting as low of a dead volume as possible – can also be used to prepare and dilute stock, spiking, and measurement solutions. Generally, equipment and solutions must have reached room temperature to avoid deviations in the pipetted volumes; non-linear calibration curves may otherwise result (Kolb and Ettre 2006).

Whether calibration in water, similar to other analytical procedures, is possible and expedient must be tested in each individual case. Due to the high volatility of most target substances in headspace analysis, it is however expected that calibration in matrix is preferable, especially with respect to analyte losses and reproducibility.

#### 4.4.2 Internal standards (ISTDs)

A prerequisite for the use of an ISTD is its optimal chromatographic separation or spectrometric differentiation from the substance to be analysed. The concentration of the ISTD in the gas phase should, if possible, be in the same range as that of the analyte. Furthermore, the analyte and the ISTD should be as similar as possible with regard to physico­chemical behaviour, such as vapour pressure.

For example, alcohols are used as ISTDs for the analysis of alcohols, and aromatic hydrocarbons are used for aromatics. Due to similar polarities, these structurally analogous compounds are subject to the same matrix effects as the analytes and can therefore compensate for matrix differences between samples. ISTDs with a wide range of applications include such substances as *tert‑*butanol, benzene, 2‑butanone (methyl ethyl ketone), and acetone. Structurally identical isotope-labelled compounds, which differ from the analyte by a mass difference of at least 2 daltons, are especially suitable for mass-spectrometric detection. Such standards are, however, not available for all target analytes.

The ISTD is usually added to the sample to be analysed in an aqueous or alcoholic solution. For samples which have already been transferred into headspace vials, the ISTD can also be injected through the septum using an injection syringe to avoid opening the headspace vial again. As opening the vial may lead to analyte loss or sample contamination, the addition of an ISTD may be skipped if it is not necessary for analytical reliability.

#### 4.4.3 Control materials

For headspace analysis, as with other analytical procedures, quality assurance of the analytical results should be carried out as stipulated in the guidelines of the *Bundesärztekammer* (German Medical Association) as well as in the corresponding general chapter published by the Commission (Bader et al. 2010; Bundesärztekammer 2023).

To check precision, each analytical run includes at least one quality-control sample exhibiting a constant concentration of the analytes in question. As no control materials are commercially available for headspace analysis and, as a result, no certified control materials are available, they must be prepared in the in-house laboratory. To this end, pooled urine or whole blood is spiked with corresponding amounts of the analytes; the material is then aliquoted in headspace vials and stored frozen at around −20 °C. The stability of the materials thus prepared and stored is verified by control cards.

Regarding the stability of self-prepared quality-control material for the determination of aromatic compounds and other solvents in blood, Heinrich-Ramm et al. (2004) concluded that these materials are only stable for a few months and are therefore only partially suitable for long-term quality control.

In order to assess the accuracy of an analytical procedure, external quality-assurance programmes should be used in addition to internal quality-assurance procedures. The G‑EQUAS, which was initiated by the German Society of Occupational and Environmental Medicine (*Deutsche Gesellschaft für Arbeitsmedizin und Umweltmedizin*, DGAUM), is the only programme to offer interlaboratory comparisons for a broad range of headspace parameters relevant to occupational medicine worldwide. This interlaboratory comparison is conducted biannually, encompassing a total of four materials: benzene, toluene, xylene (*m*-, *o*-, *p*-), and ethylbenzene in blood and urine; di­chloro­methane, tri­chloro­methane, tetra­chloro­methane, 1,2‑dichloroethane, 1,1,1‑tri­chloro­ethane, tri­chloro­ethene, and tetra­chloro­ethene in blood; as well as methanol, *n‑*butanol, acetone, 2‑butanone (methyl ethyl ketone), methyl *n‑*butyl ketone, methyl isobutyl ketone, tetra­hydro­furan, and methyl *tert‑*butyl ether in urine.

## 5 Published HBM methods and assessment values

### 5.1 Published HBM methods

#### 5.1.1 Methods published by the Commission

Until mid 2025, the “Analyses in Biological Material” working group had published a total of 36 headspace methods with which biomonitoring can be carried out specifically and sensitively for 66 hazardous substances. Tables 2, 3 and 4 provide an overview of headspace methods for human biomonitoring in urine, blood and exhaled air which have been published by the Commission.

Between 1978 and 1983, sixteen HS‑GC methods were summarised as part of a collective method on headspace techniques, covering a wide range of industrially applied solvents (Machata and Angerer 1983). With the exception of acetone, which could be determined in both blood and urine, the determination of these parameters was described exclusively for the matrices blood or serum.

Further headspace methods for individual substances were published between 1980 and 1988. These methods enable the quantification of formic acid in urine (Angerer and Schaller 1980), tri­chloro­acetic acid (Angerer and Eben 1980) and trifluoroacetic acid (Dallmeier and Müller 1982) in blood, cyanide in blood (Eben and Lewalter 1988), as well as the determination of the CO‑Hb level in blood (Angerer and Zorn 1985). These methods were not included in the collective method on headspace techniques because they require thermal decomposition (tri­chloro­acetic acid) (Angerer and Eben 1980), esterification (trifluoroacetic acid) (Dallmeier and Müller 1982), release by acidification (cyanide) (Eben and Lewalter 1988), or catalytic conversion of the analyte (formic acid; CO‑Hb) (Angerer and Schaller 1980; Angerer and Zorn 1985) and were thereby inconsistent with the general approach of the collective method.

At the beginning of the 1990s, methods were published on the determination of halogenated hydrocarbons (Angerer et al. 1991) and on the determination of benzene and alkylbenzenes (Angerer et al. 1994). The detection limits, which are lower than those of the previously published methods by a factor of 2 (halogenated hydrocarbons) or 5 (benzene and alkylbenzenes), exemplify the further development of laboratory technology.

With the method on the determination of alcohols and ketones in blood and urine (Angerer et al. 1997) published in 1997, numerous alcoholic substances were included in the method collection for the first time and, moreover, a wide spectrum of parameters became available for determination in the urinary matrix. In 2012, the method was extended to another parameter with the addendum on “tetra­hydro­furan (THF) in urine” (Blaszkewicz and Angerer 2013); as such, a total of twelve analytes can now be determined simultaneously in a single analytical run.

The prevalence and constant development of headspace‑GC coupled to MS as a sensitive and reliable procedure for analyte determination in biological materials has made it necessary to revise and update the analytical methods published by the “Analyses in Biological Materials” working group. The methylmercury method for blood (Hoppe and Heinrich-Ramm 2006) was the first HS‑GC method with mass-spectrometric detection to be published as part of the method collection. The methods on the determination of tri­chloro­acetic acid in urine (Will et al. 2017), methyl *tert‑*butyl ether in blood and urine (Hoppe et al. 2018), aromatic compounds in blood (Göen et al. 2018) and in urine (Van Pul et al. 2018), alcohols, ketones and ethers in urine (Göen et al. 2020) and halogenated hydrocarbons in blood (Göen et al. 2021) are further methods which use mass spectrometry as a proven, state-of-the-art detection procedure. Furthermore, as with Van Pul et al. (2018) and their use of the ITEX technique or Roßbach et al. (2019) and their use of SPDE enrichment, new dynamic headspace techniques are increasingly coming into use which enable demonstrably more sensitive analyses.

A headspace method for the determination of furan in exhaled air was recently developed and adopted in the working group “Analyses in Biological Materials” (Ziener et al. 2024), as no methods could be developed for the matrices blood or urine that would have allowed the reliable detection and assessment of furan exposure.

#### 5.1.2 Internationally published biomonitoring methods

Literature research was conducted to compile an overview of internationally published headspace methods for the ­determination of biomonitoring parameters in blood and urine. This survey was carried out using PubMed and Scopus with the search terms: (1) “headspace” AND “urine” AND “occupational” or (2) “headspace” AND “blood” AND “occu­pa­tion­al”. Duplicates or publications without description of headspace methods were excluded based on a manual screening of titles and abstracts. The relevant information on the applied analytical procedures was extracted via a full-text search of the remaining studies. Method publications which reported no information on limits of detection or quantitation were excluded.

Headspace methods have also been developed and published for other matrices, such as saliva, exhaled air, breath condensate, or tissue samples. These papers were largely not considered here, as quantitative analyses in occupational-medical human biomonitoring have only been established for determination in urine as well as blood, serum and plasma due to the mostly well-known kinetics of the substances (absorption, distribution, metabolism, excretion) in these matrices. For this reason, most assessment values for biological materials – such as BAT, BLW or BAR – refer to these matrices.

Tables 5and 6 provide an overview of headspace methods for biomonitoring parameters in urine and blood, serum, and plasma which have been published in the international literature. For this purpose, the analytes were separated into the following groups: “aromatic hydrocarbons”, “aliphatic hydrocarbons”, “halogenated hydrocarbons”, “alcohols, aldehydes, ketones, and ethers”, “inhalational anaesthetics”, and “others.” The analytical methods utilised are given as well as the detection and quantitation limits achieved and, for multimethods, the number of analytes that can be determined in parallel. As one might expect, these tables predominantly include methods on the determination of volatile hydrocarbons, such as the determination of BTEX aromatics in blood, chlorinated hydrocarbons (CHCs) in blood, or alcohols and ketones in urine.

A more detailed look at the publications shows that older methods especially focused on volatile substances which, at least in the past, emerged in the workplace and in human biological materials, in rather high concentrations. As such, classic static headspace techniques without additional enrichment were sufficient to measure these exposure levels. At first, rather unspecific detection methods were mainly used, such as flame-ionisation detection for aliphatic and aromatic hydrocarbons (e.g. Kawai et al. 2003) or electron-capture detection for halogenated hydrocarbons (e.g. da Silva et al. 1999). Enrichment techniques prior to sample injection as well as mass-spectrometric detection (e.g. Rutkiewicz et al. 2011) have been increasingly used in recent years to achieve lower limits of detection and quantitation as well as more reliable analytical results.

Due to improvements in analytical sensitivity and specificity, it is now also possible to quantify analytes that are only excreted with the urine to a very small extent. This trend is reflected in the literature review for substances such as benzene, toluene, and *m*-, *o*-, and *p‑*xylene and, due to the longer half-lives of these substances in the urinary matrix compared to blood, enables a more reliable determination of occupational exposure. Furthermore, urine collection is non-invasive and is more accepted by the workers than drawing blood.

A common and widely known application of HS‑GC is blood alcohol determination, which is mostly used in forensics in the context of traffic offenses. For this process, alcohol concentration must be consistently determined using two ­independent procedures (Aderjan et al. 2011). One procedure for blood alcohol determination which has been authorised for forensic purposes is based on the static HS‑GC‑FID method by Machata from the year 1964 (Kolb and Ettre 2006; Machata 1967), which represents the beginnings of quantitative HS‑GC. In addition to flame-ionisation detectors, mass spectrometers are now also employed for detection (Cordell et al. 2013). An international interlaboratory-comparison programme for the determination of ethanol in blood and serum is offered by the German Society of Toxicological and Forensic Chemistry (*Gesellschaft für Toxikologische und Forensische Chemie*, GTFCh) (http://www.arvecon.de/gb/).

The scientific literature includes methods which appear questionable in terms of their practical application regarding physicochemical prerequisites and the limitations of headspace analysis. In these methods, for example, low-volatility compounds with very high K values are quantified using headspace techniques: chlorophenols (2‑MCP; 2,4‑DCP; 2,4,6‑TriCP; 2,3,4,6‑TeCP; and PCP) in human urine (without hydrolysis) by headspace‑SPME‑GC‑MS (Lee et al. 1998), organochlorine pesticides (HCB, *β*‑HCH, heptachlor epoxide, DDE, and DDT) and PCBs in human serum by headspace‑SPME‑GC‑ECD (López et al. 2007), organochlorine pesticides (HCB, heptachlor, DDEs, DDTs, DDDs, chlordane, dieldrin, etc.) in human serum by headspace‑SPME‑GC‑MS (Kim et al. 2013), dinitroaniline herbicides in blood and urine by headspace‑SPME‑GC‑ECD (Guan et al. 1998), or persistent organic pollutants (POP pesticides and PCBs) in human serum by headspace‑SPME‑GC‑MS (Flores-Ramírez et al. 2014). These methods were not included in the tabular overview (Tables 5and 6).

In the following, some headspace applications for the fields of occupational and environmental medicine are indicated as examples of the use of alternative matrices: on one hand, methods for the determination of benzene (Menezes et al. 2009), styrene (Fields and Horstman 1979; Guillemin and Berode 1988), or 1,1,2‑tri­chloro-1,2,2‑tri­fluoro­ethane (Woollen et al. 1990) in exhaled air have been published as well as a method for the determination of toluene in breath condensate (Maniscalco et al. 2006). On the other hand, there are also methods for the determination of 2‑butanone (methyl ethyl ketone), isopropyl alcohol, and *N*,*N*‑dimethylformamide in the saliva of leather-industry workers (Wang and Lu 2009) and of 2‑ring to 4‑ring PAH in the saliva of both smokers and non-smokers (Martín Santos et al. 2020) as well as a method for the determination of toluene, ethylbenzene, xylene, and styrene in saliva (Gherardi et al. 2010). Finally, headspace methods and applications have been published which use tissue samples as a matrix. Examples include the determination of nitromethane as a metabolite of chloropicrin in pig-liver samples by static headspace GC‑MS (Halme et al. 2015), the determination of ethyl glucuronide in placental tissue and placental perfusate by HS‑SPME‑GC‑MS (Matlow et al. 2012), or the determination of 1,1‑difluoroethane in blood, urine, and brain samples by static HS‑GC‑FID (Avella et al. 2008).

### 5.2 Assessment values for HBM

The Commission has established assessment values for numerous parameters which are or can be determined with headspace methods. Additional assessment values have been issued by other scientific organisations, particularly the Committee for Risk Assessment (RAC) of the European Chemicals Agency (ECHA) (RAC 2025) and the American Conference of Governmental Industrial Hygienists (ACGIH) (ACGIH 2025). Table 7 provides an overview of these values. The assessment values were primarily established for parameters for which a suitable headspace technique has long been available, such as for BTEX aromatic compounds and short-chained halogenated hydrocarbons in blood as well as for alcohols, ketones, and ethers in urine. The majority of these assessment values are toxicologically based limit values (BAT, BEI, BLV) which enable the evaluation of a potential health risk. There are further assessment values which apply especially to carcinogenic hazardous substances, which either enable the occupational exposure to be differentiated from the general background exposure (BAR) or which can be linked to a defined, additional lifetime cancer risk via an exposure-risk relationship. For this purpose, exposure equivalents for carcinogenic substances (*Expositionsäquivalente für krebserzeugende Arbeitsstoffe*, EKA) have been established for such biomonitoring parameters as “benzene in urine” and “tetra­chloro­ethene in whole blood” (DFG 2025).

In addition to assessment values published by scientific panels, data from population studies can be consulted to receive information on general background exposure. Table 8 summarises data published in the international ­literature. Table 9 shows the background levels of various parameters that were determined with headspace pro­cedures for the U.S. general population as part of NHANES (National Health and Nutrition Examination Survey) conducted by the Centers for Disease Control and Prevention (CDC). It is important to note that, in principle, assessment values reached by expert consensus (Table 7) are considerably more robust. For reference values, it is imperative to account for regional representativeness, effects of subgroups and lifestyle, as well as limited validity due to changing levels of background exposure (Göen et al. 2012).

Regardless of the type of assessment value, it is essential to adhere to prescribed sampling times for the determination of volatile compounds typically analysed by headspace techniques. As, for example, volatile hydrocarbons are excreted from the blood quite rapidly, sample collection must take place immediately after the end of exposure. The half-lives of the most important hazardous substances which can be determined by headspace analysis are listed in Table 1.

## 6 Summary

Gas-chromatographic headspace analysis uses well-known and reproducible physicochemical distribution processes for the separation of volatile compounds from their biological matrices. The main advantages of what is called “headspace analysis” include the highly efficient separation of the analytes from matrix, sample preparation which usually requires very few steps, and its excellent automatisation capabilities.

The major challenges in the application of the headspace analysis in the practice of occupational and environmental medicine are as follows:

- to define exact sampling conditions (especially sampling time)
- to avoid contamination and analyte loss in the pre-analytical phase
- to calibrate the procedures adequately (especially with regard to matrix selection and preparation of comparative standards)

In general, the parameters necessary for the practice of occupational and environmental medicine are sufficiently covered by the headspace procedures presented in this review: the procedures developed and published by the Commission as well as the other methods described in the scientific literature. Especially the newer procedures exhibit a detection sensitivity which also enables the determination of parameters in the range of the background exposure of the general population. This development can be attributed to the increased application of enrichment techniques as well as the use of mass spectrometry as a standard detection method. Despite its long history and its range of applications, which is limited to volatile compounds, headspace analysis remains an important procedure for human biomonitoring in the fields of occupational and environmental medicine.

**Tab.1:** Half-lives of the most prominent hazardous substances measurable by headspace analysis

Substance (synonym)	Analyte	Material	Excretion maximum	Elimination kinetics	Half-life	References
acetone	acetone	alveolar air	–	–	4.3 ± 1.1 h	Wigaeus et al. 1981
		blood	–	linear	3 h	DiVincenzo et al. 1973
			–	–	5.8 h	Wang et al. 1994
		capillary blood	–	mono­exponen­tial	4.3 ± 1.0 h	Ernstgård et al. 1999
		venous blood	–	–	6.1 ± 0.7 h	Wigaeus et al. 1981
		arterial blood	–	–	3.9 ± 0.7 h	Wigaeus et al. 1981
		urine	3–3.5 h	–	–	Wigaeus et al. 1981
			2–4 h	–	8 h	Pezzagno et al. 1986
			2 h	biphasic	8–9 h	Blaszkewicz et al. 1991
benzene	benzene	exhaled air	–	triphasic	0.7–1.7 h; 3–4 h; 20 h	Sherwood 1972
			–	–	4 h; 4 d	Sato et al. 1975
		blood	–	exponential	≈ 30 min	Angerer 1983
2-butanone (methyl ethyl ketone)	2-butanone	exhaled air	–	–	40–60 min	Ong et al. 1991; Tada et al. 1972
		blood	–	biphasic	30 min; 81 min	Liira et al. 1988
			–	first order	49 min	Brown et al. 1987; Dick et al. 1988
			–	–	270 min (mathematical model)	Angerer 1995
		urine	–	–	1.5 h (1–2.3 h) (after inhalation exposure and dermal uptake from vapour phase); 2.7 h (2.3–4.3 h) (after dermal uptake from vapour phase)	Brooke et al. 1998
carbon monoxide	CO-Hb	blood	–	biphasic	1.6 h; 30.9 h	Cronenberger et al. 2008
			–	–	320 min (128–409 min)	Peterson and Stewart 1975
chlorobenzene	chlorobenzene	blood	–	biphasic	53 min; 150 min	Knecht and Woitowitz 2000
cyclohexane, cyclohexanone, cyclohexanol	cyclohexanol	urine	end of exposure	–	1.5 h	Mráz et al. 1998
di­chloro­methane (methylene chloride)	di­chloro­methane	blood	–	–	5–40 min	Riley et al. 1966 according to ACGIH 2005
			–	–	4.3 h and 8.1 h (n = 2; 36 h after acute poisoning)	Poli et al. 2005
		urine	–	–	40 min	DiVincenzo et al. 1972
			end of exposure	–	210–410 min	Sakai et al. 2002
			–	–	3.8 h and 7.5 h (n = 2; 36 h after acute poisoning)	Poli et al. 2005
ethylbenzene	ethylbenzene	alveolar air	–	multiphasic	t_1_: < 1 h	Tardif et al. 1997
		blood	–	biphasic	0.5 h; 1.81 h	Knecht et al. 2000
			–	multiphasic	t_1_: < 1 h	Tardif et al. 1997
		urine	–	biphasic	0.69 h; 19.2 h	Janasik et al. 2008
halothane (2-bromo-2‑chloro-1,1,1-tri­fluoro­ethane)	halothane	exhaled air	–	linear, triphasic	t_1_: 20–30 min; t_3_: 2 h	Henschler 1995
	trifluoroacetic acid	blood	–	–	40–60 h	Henschler 1995
		urine	–	–	48–66 h	Henschler 1995
*n*-heptan	1-heptanol	urine	3.15 h	multiphasic	t_1_: 1.70 h; t_2_: 9.68 h	Rossbach et al. 2018
	2-heptanol	urine	3.24 h	multiphasic	t_1_: 1.46 h; t_2_: 8.26 h	Rossbach et al. 2018
	3-heptanol	urine	3.24 h	multiphasic	t_1_: 1.46 h; t_2_: 7.99 h	Rossbach et al. 2018
	4-heptanol	urine	3.32 h	multiphasic	t_1_: 1.60 h; t_2_: 7.75 h	Rossbach et al. 2018
	2-heptanone	urine	5.48 h	multiphasic	t_1_: 2.53 h; t_2_: n. a.	Rossbach et al. 2018
	3-heptanone	urine	3.10 h	multiphasic	t_1_: 2.14 h; t_2_: 9.05 h	Rossbach et al. 2018
	heptan-2,5-dione	urine	3.92 h	multiphasic	t_1_: 2.87 h; t_2_: 8.85 h	Rossbach et al. 2018
			–	–	3.4 ± 1.5 h	Filser et al. 1996
isopropylbenzene (cumene)	isopropylbenzene	blood	–	biphasic	0.49 h; 1.61 h	Knecht et al. 2000
methanol	methanol	exhaled air	–	monophasic	1.5 h	Dutkiewicz 1978
			–	–	1.38 ± 0.86 h	Batterman et al. 1998
		blood	–	first-order	2.25 h	Ferry et al. 1980 a, b
			–	–	1.44 ± 0.33 h	Batterman et al. 1998
		urine	–	–	1.5–2.0 h	Šedivec et al. 1981
			–	–	1.55 ± 0.67 h	Batterman et al. 1998
methyl *tert*‑butyl ether (2‑methoxy-2‑methylpropane)	methyl *tert*‑butyl ether	exhaled air	–	–	1.3–2.9 min	Lindstrom and Pleil 1996
		alveolar air	–	triphasic	0.25 ± 0.07 h; 0.64 ± 0.15 h; 1.74 ± 0.23 h after oral administration of 15 mg MTBE	Amberg et al. 2001
		blood	–	–	35 min	Prah et al. 1994
			–	fourphasic	1 min; 10 min; 1.5 h; 19 h	Nihlén et al. 1998
			–	–	1.8 ± 0.3 h after exposure to 4.5 ± 0.4 ppm MTBE for 4 h or 2.6 ± 0.9 h after exposure to 38.7 ± 3.2 ppm MTBE for 4 h	Amberg et al. 1999
			–	triphasic	0.7 ± 0.2 h; 1.2 ± 0.3 h; 3.7 ± 0.9 h after oral administration of 15 mg MTBE or 0.8 ± 0.1 h; 1.8 ± 0.3 h; 8.1 ± 3.0 h after oral administration of 5 mg MTBE	Amberg et al. 2001
		urine	–	linear, biphasic	20 min; 3 h	Nihlén et al. 1998
			–	–	5.2 ± 1.0 h after exposure to 4.5 ± 0.4 ppm MTBE for 4 h or 4.3 ± 1.4 h after exposure to 38.7 ± 3.2 ppm MTBE for 4 h	Amberg et al. 1999
			–	–	5.5 ± 2.0 h after oral administration of 15 mg MTBE or 3.4 ± 0.9 h after oral administration of 5 mg MTBE	Amberg et al. 2001
methyl *tert*‑butyl ether (2‑methoxy-2‑methyl­propane)	*tert*‑butanol	alveolar air	–	linear	6.71 ± 2.17 h after oral administration of 15 mg MTBE	Amberg et al. 2001
		blood	–	–	10 h	Nihlén et al. 1998
			–	–	6.5 ± 2.1 h after exposure to 4.5 ± 0.4 ppm MTBE for 4 h or 5.3 ± 2.1 h after exposure to 38.7 ± 3.2 ppm MTBE for 4 h	Amberg et al. 1999
			–	linear	8.5 ± 2.4 h after oral administration of 15 mg MTBE or 8.1 ± 1.6 h after oral administration of 5 mg MTBE	Amberg et al. 2001
		urine	–	–	8.2 h	Nihlén et al. 1998
			–	–	12.0 ± 3 h after exposure to 4.5 ± 0.4 ppm MTBE for 4 h or 10.4 ± 1.8 h after exposure to 38.7 ± 3.2 ppm MTBE for 4 h	Amberg et al. 1999
			–	–	8.1 ± 1.4 h after oral administration of 15 mg MTBE or 7.7 ± 2.0 h after oral administration of 5 mg MTBE	Amberg et al. 2001
4-methyl-2‑pentanone (methyl isobutyl ketone)	4-methyl-2‑pentanone	blood	–	biphasic	12 min (0–30 min after exposure); 71 min (60–180 min after exposure)	Wigaeus Hjelm et al. 1990
		urine	–	biphasic	≈ 40 min; 6.9 h	Ogata et al. 1995 according to ACGIH 2010 a
2-propanol (isopropanol)	2-propanol	blood/serum	–	first-order	3–6.4 h (acute poisoning)	Lacouture et al. 1983; Natowicz et al. 1985
		blood	–	linear, first-order	2.5–3 h	Bohn et al. 1987; Daniel et al. 1981
	acetone	blood/serum	–	first-order	22.4–24 h (acute poisoning)	Hawley and Falko 1982; Natowicz et al. 1985
styrene	styrene	exhaled air	–	biphasic	13–52 min; 4–20 h	ACGIH 2015
		blood	–	biphasic	0.58 ± 0.08 h; 13.0 ± 0.8 h	Ramsey et al. 1980
		urine	–	–	20 h	Prieto et al. 2002
tetra­chloro­ethene	tetra­chloro­ethene	exhaled air	–	biphasic	< 3 h; 65 h	Stewart et al. 1970 according to ACGIH 2009
			–	–	3 d (mathematical model, terminal phase)	Guberan and Fernandez 1974
			–	triphasic	3–10 min; 25–60 min; 210–220 min	Chien 1997
		blood	–	triphasic	15 min; 4 h; 4 d (mathematical model)	Guberan and Fernandez 1974
			–	triphasic	12–16 h; 30–40 h; 55–65 h	Monster et al. 1979
	tri­chloro­acetic acid	blood	–	–	50–100 h	Müller et al. 1974; Triebig et al. 1976
tetra­chloro­methane (carbon tetrachloride)	tetra­chloro­methane	alveolar air	–	exponential	2.7 h after exposure to 10 ppm tetra­chloro­methane for 3 h	Stewart et al. 1961
tetra­hydro­furan	tetra­hydro­furan	alveolar air	–	exponential	32 ± 12.7 min	Kageyama 1988 according to ACGIH 2008 a
		urine	–	monophasic	2.5 h	Kageyama 1988 according to ACGIH 2008 a
			–	monophasic	118 min	JSOH 2014
			–	biphasic	0.9–1.2 h; 4–5 h	Jones 2023
toluene	toluene	exhaled air	–	triphasic	0.4 h; 3.9 h; 39 h	Pierce et al. 2004 according to ACGIH 2010 b
		alveolar air	–	exponential	17.5–20.8 h (30–120 h after accidental event)	Brugnone et al. 1983
			–	–	3.8 h (2.6–6 h)	Brugnone et al. 1986
		blood	–	exponential	17.1–27.1 h (30–120 h after accidental event)	Brugnone et al. 1983
			–	–	4.5 h (3–6.2 h)	Brugnone et al. 1986
			–	triphasic	3 min, 40 min, 738 min	Löf et al. 1993
			–	biphasic	0.5 h; 1.94 h	Knecht et al. 2000
			end of exposure	triphasic	0.1–0.7 h; 1–12 h; 15–39 h	Pierce et al. 2004 according to ACGIH 2010 b
		urine	3 h	exponential, biphasic	≈ 0.5 h; 5 h	Ducos et al. 2008
			–	exponential, biphasic	0.88 h, 12.9 h	Janasik et al. 2008
1,1,1-tri­chloro­ethane	1,1,1-tri­chloro­ethane	exhaled air	–	triphasic	9 h; 20 h; 26 h (up to 100 h after end of exposure)	Monster et al. 1979
		blood	–	triphasic	9 h; 20 h; 26 h (up to 100 h after end of exposure)	Monster et al. 1979
			–	triphasic	44 min; 5.7 h; 53 h	Nolan et al. 1984
			–	mono­exponen­tial (from 30 h after end of exposure)	40 h (from 30 h after end of exposure)	Bolt 1994
tri­chloro­ethene	tri­chloro­ethene	exhaled air	–	exponential	25 h (30–80 h after end of exposure)	Stewart et al. 1970 b, according to Ikeda and Imanura 1973
			end of exposure	exponential, multiphasic	–	Müller et al. 1974
		blood	end of exposure	exponential, multiphasic	–	Müller et al. 1974
			–	triphasic	20 min; 3 h; 30 h	Fernández et al. 1975 according to ACGIH 2008 b
			–	–	21.7 h (17.3–24.3 h) (acute poisoning)	Kostrzewski et al. 1993
	tri­chloro­acetic acid	blood	–	–	50–100 h	Müller et al. 1974; Triebig et al. 1976
1,3,5-tri­methyl­benzene (mesitylene)	1,3,5-tri­methyl­benzene	urine	–	exponential, biphasic	0.45 h; 6.7 h	Janasik et al. 2008
xylene	xylene	exhaled air	–	biphasic	1 h, 20 h	Åstrand et al. 1978; Šedivec and Flek 1976
		alveolar air	–	triphasic	0.8 h; 7.7 h; 17.3 h	Riihimäki et al. 1979
		blood	–	multiphasic	t_1_: 0.5 h	Åstrand et al. 1978
			–	biphasic	0.48 h; 1.82 h	Knecht et al. 2000
		urine	–	biphasic	0.84 h; 10.9 h	Janasik et al. 2008

For abbreviations, see List of abbreviations.

**Tab.2:** Headspace methods for the matrix urine published by the Commission

Hazardous substance (synonym)	Analyte	Multimethod (number of analytes)	Detection limit [μg/l]	Quantitation limit [μg/l]	Analytical method	References
**aromatic hydrocarbons**						
benzene	benzene	yes (8)	0.007	0.021	dynamic HS-GC-MS	Van Pul et al. 2018
ethylbenzene	ethylbenzene		0.010	0.030		
isopropylbenzene (cumene)	isopropylbenzene		0.012	0.036		
styrene	styrene		0.014	0.042		
toluene	toluene		0.029	0.087		
*m*‑xylene	*m*‑xylene		0.011	0.033		
*o*‑xylene	*o*‑xylene		0.015	0.045		
*p*‑xylene	*p*‑xylene		0.011	0.033		
**halogenated hydrocarbons**						
bromomethane (methylbromide)	formic acid	–	200	n. a.	HS-GC-FID	Angerer and Schaller 1980
halothane (2‑bromo-2‑chloro-1,1,1‑tri­fluoro­ethane)	trifluoroacetic acid	–	< 10	n. a.	HS-GC-ECD	Dallmeier and Müller 1982
1,1,2,2‑tetrachloroethane	tri­chloro­acetic acid	yes (4)	10	30	HS-GC-MS	Will et al. 2017
tetrachloroethene	trichloroacetic acid		10	30		
1,1,1‑tri­chloro­ethane	tri­chloro­acetic acid		10	30		
tri­chloro­ethene	tri­chloro­acetic acid		10	30		
1‑bromopropane	1‑bromopropane	yes (2)	0.01	0.03	dynamic HS-GC-MS	Roßbach et al. 2019
2‑bromopropane	1‑bromopropane		0.01	0.04		
**alcohols, aldehydes, ketones, and ethers**						
acetone	formic acid	–	200	n. a.	HS‑GC‑FID	Angerer and Schaller 1980
methanol	formic acid	–	200	n. a.		
acetone	acetone	–	10 000	n. a.	HS‑GC‑FID	Machata and Eben 1980
acetone	acetone	yes (11)	100	n. a.	HS‑GC‑FID	Angerer et al. 1997
1‑butanol	1‑butanol		300	n. a.		
2‑butanol	2‑butanol		200	n. a.		
2‑butanone (methyl ethyl ketone)	2‑butanone		80	n. a.		
ethanol	ethanol		800	n. a.		
2‑hexanone	2‑hexanone		30	n. a.		
isobutanol (2‑methyl-1‑propanol)	isobutanol		200	n. a.		
methanol	methanol		600	n. a.		
methyl formate	methanol		600	n. a.		
4‑methyl-2‑pentanone (methyl isobutyl ketone)	4‑methyl-2‑pentanone		30	n. a.		
1‑propanol	1‑propanol		400	n. a.		
2‑propanol (isopropanol)	2‑propanol		400	n. a.		
2‑propanol (isopropanol)	acetone		100	n. a.		
tetra­hydro­furan	tetra­hydro­furan	–	100	300	HS‑GC‑FID	Blaszkewicz and Angerer 2013
methyl *tert*‑butyl ether (2-methoxy-2‑methylpropane)	methyl *tert*‑butyl ether	–	1.8	6	HS‑GC‑MS	Hoppe et al. 2018
acetone	acetone	yes (27)	10	30	HS‑GC‑MS	Göen et al. 2020
1‑butanol	1‑butanol		100	300		
2‑butanol	2‑butanol		50	150		
*tert*‑butanol	*tert*‑butanol		50	150		
2‑butanone (methyl ethyl ketone)	2‑butanone		10	30		
cyclohexanone	cyclohexanone		50	150		
cyclopentanone	cyclopentanone		50	150		
3,3‑dimethyl-2‑butanone (methyl *tert*‑butyl ketone)	3,3‑dimethyl-2‑butanone		10	30		
1,4‑dioxane	1,4‑dioxane		100	300		
ethanol	ethanol		100	300		
2‑heptanone	2‑heptanone		10	30		
3‑heptanone	3‑heptanone		10	30		
4‑heptanone	4‑heptanone		10	30		
2‑hexanone	2‑hexanone		10	30		
3‑hexanone	3‑hexanone		10	30		
Isobutanol (2-methyl-1-propanol)	isobutanol		50	150		
methanol	methanol		200	600		
3‑methyl-2‑butanone (methyl isopropyl ketone)	3‑methyl-2‑butanone		10	30		
methyl *tert*‑butyl ether (2-methoxy-2‑methylpropane)	methyl *tert*‑butyl ether		5	15		
methyl *tert*‑butyl ether (2-methoxy-2‑methylpropane)	*tert*‑butanol		50	150		
4‑methyl-2‑pentanone (methyl isobutyl ketone)	4‑methyl-2‑pentanone		10	30		
2‑pentanone	2‑pentanone		20	60		
3‑pentanone	3‑pentanone		20	60		
1‑propanol	1‑propanol		30	90		
2‑propanol (isopropanol)	2‑propanol		20	60		
2‑propanol (isopropanol)	acetone		10	30		
tetra­hydro­furan	tetra­hydro­furan		10	30		

For abbreviations, see List of abbreviations.

**Tab.3:** Headspace methods for the matrix blood published by the Commission

Hazardous substance (synonym)	Analyte	Multimethod (number of analytes)	Detection limit [μg/l] (unless otherwise specified)	Quantitation limit [μg/l]	Analytical method	References
**aromatic hydrocarbons**						
styrene	styrene	–	50	n. a.	HS-GC-FID	Schaller et al. 1980
benzene	benzene	yes (6)	20	n. a.	HS-GC-FID	Knecht and Angerer 1983
ethylbenzene	ethylbenzene		20	n. a.		
toluene	toluene		40	n. a.		
*m*-xylene	*m*-xylene		40	n. a.		
*o*-xylene	*o*-xylene		40	n. a.		
*p*-xylene	*p*-xylene		40	n. a.		
isopropylbenzene (cumene)	isopropylbenzene	–	86	n. a.	HS-GC-FID	Goenechea and Machata 1983
benzene	benzene	yes (5)	3	n. a.	HS-GC-FID	Angerer et al. 1994
ethylbenzene	ethylbenzene		8	n. a.		
toluene	toluene		5	n. a.		
*m*-xylene	*m*-xylene		8	n. a.		
*o*-xylene	*o*-xylene		8	n. a.		
benzene	benzene	yes (14)	0.7	2.1	HS-GC-MS	Göen et al. 2018
chlorobenzene	chlorobenzene		0.9	2.7		
ethylbenzene	ethylbenzene		0.9	2.7		
isopropylbenzene (cumene)	isopropylbenzene		1.0	3.0		
1-propylbenzene	1-propylbenzene		1.0	3.0		
styrene	styrene		1.0	3.0		
1,2,3,5-tetra­methyl­ben­zene (isodurol)	1,2,3,5-tetra­methyl­ben­zene		3.0	9.0		
toluene	toluene		0.7	2.1		
1,2,3-tri­methyl­benzene (hemimellitene)	1,2,3-tri­methyl­benzene		1.5	4.5		
1,2,4-tri­methyl­benzene (pseudocumene)	1,2,4-tri­methyl­benzene		1.5	4.5		
1,3,5-tri­methyl­benzene (mesitylene)	1,3,5-tri­methyl­benzene		1.5	4.5		
*m*-xylene	*m*-xylene		0.9	2.7		
*o*-xylene	*o*-xylene		0.9	2.7		
*p*-xylene	*p*-xylene		0.9	2.7		
**halogenated hydrocarbons**						
halothane (2-bromo-2-chloro-1,1,1-tri­fluoro­ethane)	halothane	–	50	n. a.	HS-GC-ECD	Schaller et al. 1978
1,1,1,2-tetrachloroethane	tri­chloro­acetic acid	–	200	n. a.	HS-GC-ECD	Angerer and Eben 1980
tetra­chloro­ethene	tri­chloro­acetic acid	–	200	n. a.		
tri­chloro­ethene	tri­chloro­acetic acid	–	200	n. a.		
1,1‑dichloroethane	1,1‑dichloroethane	–	100	n. a.	HS‑GC‑FID	Zorn et al. 1982
1,2-dichloroethane	1,2-dichloroethane	–	82	n. a.	HS-GC-FID	Angerer et al. 1981
1,1,2-tri­chloro-1,2,2-tri­fluoro­ethane^a)^	1,1,2-tri­chloro-1,2,2-tri­fluoro­ethane^a)^	–	100	n. a.	HS-GC-ECD	Schaller et al. 1982 a
tri­chloro­ethene	tri­chloro­ethene	–	50	n. a.	HS-GC-ECD	Schaller et al. 1982 b
trifluoroacetic acid	trifluoroacetic acid	–	< 10	n. a.	HS-GC-ECD	Dallmeier and Müller 1982
di­chloro­methane (methylene chloride)	di­chloro­methane	yes (4)	50	n. a.	HS-GC-ECD	Angerer and Zorn 1982
tetra­chloro­ethene	tetra­chloro­ethene		1.2	n. a.		
tetra­chloro­methane (carbon tetrachloride)	tetra­chloro­methane		0.5	n. a.		
tri­chloro­ethene	tri­chloro­ethene		1.5	n. a.		
1,1,2-tri­chloro­ethane	1,1,2-tri­chloro­ethane	–	200	n. a.	HS-GC-ECD	Eben et al. 1983
1,2-dichloroethene	1,2-dichloroethene	yes (8)	55	n. a.	HS-GC-ECD	Angerer et al. 1991
di­chloro­methane (methylene chloride)	di­chloro­methane		25	n. a.		
halothane (2‑bromo-2‑chloro-1,1,1‑tri­fluoro­ethane)	halothane		0.2	n. a.		
tetra­chloro­ethene	tetra­chloro­ethene		0.5	n. a.		
tetra­chloro­methane (carbon tetrachloride)	tetra­chloro­methane		0.3	n. a.		
1,1,1-tri­chloro­ethane	1,1,1-tri­chloro­ethane		1.0	n. a.		
tri­chloro­ethene	tri­chloro­ethene		1.1	n. a.		
tri­chloromethane (chloroform)	tri­chloromethane		0.8	n. a.		
1,2‑dichloroethane	1,2‑dichloroethane	yes (7)	0.1	0.3	HS-GC-MS	Göen et al. 2021
di­chloro­methane (methylene chloride)	di­chloro­methane		1.0	3.0		
tetra­chloro­ethene	tetra­chloro­ethene		0.1	0.3		
tetra­chloro­methane (carbon tetrachloride)	tetra­chloro­methane		0.1	0.3		
1,1,1‑tri­chloro­ethane	1,1,1‑tri­chloro­ethane		0.1	0.3		
tri­chloro­ethene	tri­chloro­ethene		0.1	0.3		
tri­chloromethane (chloroform)	tri­chloromethane		0.8	2.4		
**alcohols, aldehydes, ketones, and ethers**						
2-hexanol	2-hexanol	–	500	n. a.	HS-GC-FID	Eben and Barchet 1981
2-hexanone	2-hexanol	–	500	n. a.		
2-hexanone	2-hexanone	–	500	n. a.	HS-GC-FID	Eben and Pilz 1978
acetone	acetone	–	10 000	n. a.	HS-GC-FID	Machata and Eben 1980
1-butanol	1-butanol	–	250	n. a.	HS-GC-FID	Angerer and Möller 1980
cyclohexanone	cyclohexanone	–	750	n. a.	HS-GC-FID	Angerer and Eben 1981
1,4-dioxane	1,4-dioxane	–	2000	n. a.	HS-GC-FID	Eben and Machata 1981
acetone	acetone	yes (11)	200	n. a.	HS-GC-FID	Angerer et al. 1997
1-butanol	1-butanol		800	n. a.		
2-butanol	2-butanol		400	n. a.		
2-butanone (methyl ethyl ketone)	2-butanone		100	n. a.		
ethanol	ethanol		1300	n. a.		
2-hexanone	2-hexanone		70	n. a.		
isobutanol (2-methyl-1-propanol)	isobutanol		400	n. a.		
methanol	methanol		600	n. a.		
4-methyl-2-pentanone (methyl isobutyl ketone)	4-methyl-2‑pentanone		50	n. a.		
1-propanol	1-propanol		800	n. a.		
2-propanol (isopropanol)	2-propanol		600	n. a.		
2-propanol (isopropanol)	acetone		200	n. a.		
methyl *tert*‑butyl ether (2‑methoxy-2‑methylpropane)	methyl *tert*‑butyl ether	–	1.2	4	HS-GC-MS	Hoppe et al. 2018
**others**						
*n*-hexane	2-hexanol	–	500	n. a.	HS-GC-FID	Eben and Barchet 1981
carbon disulfide	carbon disulfide	–	50	n. a.	HS-GC-ECD	Eben and Barchet 1983
carbon monoxide	carbon monoxide after catalytic conversion to methane	–	0.17% CO‑Hb	n. a.	GC‑FID	Angerer and Zorn 1985
cyanide	hydrogen cyanide	–	70 (packed column); 100 (capillary column)	n. a.	HS-GC with a thermionic nitrogen detector	Eben and Lewalter 1988
cyanide-releasing compounds						
hydrogen cyanide						
sodium/potassium cyanide						
methylmercury	methylmercury	–	0.4	n. a.	HS-GC-MS	Hoppe and Heinrich-Ramm 2006

a) matrix: serum

For abbreviations, see List of abbreviations.

**Tab.4:** Headspace method for the matrix exhaled air published by the Commission

Hazardous substance	Analyte	Multimethod (number of analytes)	Detection limit [μg/l] (unless otherwise specified)	Quantitation limit [μg/l]	Analytical method	References
**alcohols, aldehydes, ketones, and ethers**						
furan	furan	–	0.00002	0.00006	HS-SPME-GC-MS/MS	Ziener et al. 2024

For abbreviation, see List of abbreviations.

**Tab.5:** Further internationally published headspace methods for the matrix urine

Analyte (synonym)	Multimethod (number of analytes)	Detection limit [μg/l]	Quantitation limit [μg/l]	Analytical method	References
**aromatic hydrocarbons**					
acenaphthene	yes (13)	0.002	0.006	HS-SPME-GC-MS	Campo et al. 2009
acenaphthylene	yes (13)	0.001	0.004	HS-SPME-GC-MS	Campo et al. 2009
anthracene	yes (13)	0.001	0.002	HS-SPME-GC-MS	Campo et al. 2009
benzene	yes (6)	0.025	n. a.	HS-SPME-GC-MS	Fustinoni et al. 1999
	yes (6)	0.005	n. a.	HS-SPME-GC-MS	Andreoli et al. 1999
	yes (4)	0.013	n. a.	static HS-GC-MS	Perbellini et al. 2002
	yes (3)	0.010	n. a.	static HS-GC-MS	Perbellini et al. 2003
	yes (6)	0.025	n. a.	PT-HS-GC-PID	Brčić Karačonji and Skender 2007
	yes (6)	0.05	n. a.	HS-SPME-GC-MS	Brčić Karačonji and Skender 2007
	yes (6)	0.015	n. a.	HS-SPME-GC-MS	Fustinoni et al. 2010
	yes (15)	0.3	1	HS-SPME-GC-MS	Song et al. 2017
	yes (5)	0.02	0.07	HS-SPME-GC-FID	Tajik et al. 2017
	yes (5)	0.04	n. a.	HS-SPME-GC-FID	Yousefi et al. 2018
	yes (11)	n. a.	0.010	dynamic HS-GC-MS	Erb et al. 2019
	yes (5)	0.42	1.40	HS-NTD-GC-FID	Saedi et al. 2020
benzo[*a*]anthracene	yes (13)	0.002	0.005	HS-SPME-GC-MS	Campo et al. 2009
benzo[*b*]fluoranthene	yes (13)	0.005	0.016	HS-SPME-GC-MS	Campo et al. 2009
benzo[*k*]fluoranthene	yes (13)	0.006	0.020	HS-SPME-GC-MS	Campo et al. 2009
benzo[*a*]pyrene	yes (13)	0.005	0.015	HS-SPME-GC-MS	Campo et al. 2009
*n*‑butylbenzene	yes (15)	0.6	2	HS-SPME-GC-MS	Song et al. 2017
*sec*‑butylbenzene	yes (15)	0.6	2	HS-SPME-GC-MS	Song et al. 2017
*tert*‑butylbenzene	yes (15)	0.6	2	HS-SPME-GC-MS	Song et al. 2017
chrysene	yes (13)	n. a.	0.005	HS-SPME-GC-MS	Campo et al. 2009
*m*‑cresol	yes (2)	7.0	n. a.	HS-SPME-GC-MS	Fustinoni et al. 2005
(*m* + *p*)‑cresol	yes (15)	0.3	1	HS-SPME-GC-MS	Song et al. 2017
*o*‑cresol	yes (2)	6.0	n. a.	HS-SPME-GC-MS	Fustinoni et al. 2005
	yes (15)	0.3	1	HS-SPME-GC-MS	Song et al. 2017
ethylbenzene	yes (6)	0.012	n. a.	HS-SPME-GC-MS	Fustinoni et al. 1999
	yes (6)	0.01	n. a.	HS-SPME-GC-MS	Andreoli et al. 1999
	yes (4)	0.017	n. a.	static HS-GC-MS	Perbellini et al. 2002
	yes (6)	0.035	n. a.	PT-HS-GC-PID	Brčić Karačonji and Skender 2007
	yes (6)	0.035	n. a.	HS-SPME-GC-MS	Brčić Karačonji and Skender 2007
	yes (6)	0.015	n. a.	HS-SPME-GC-MS	Fustinoni et al. 2010
	yes (15)	0.3	1	HS-SPME-GC-MS	Song et al. 2017
	yes (5)	0.06	0.2	HS-SPME-GC-FID	Tajik et al. 2017
	yes (5)	0.06	n. a.	HS-SPME-GC-FID	Yousefi et al. 2018
	yes (11)	n. a.	0.010	dynamic HS-GC-MS	Erb et al. 2019
	yes (5)	0.22	0.73	HS-NTD-GC-FID	Saedi et al. 2020
fluoranthene	yes (13)	n. a.	0.00426	HS-SPME-GC-MS	Campo et al. 2009
fluorene	yes (13)	n. a.	0.00462	HS-SPME-GC-MS	Campo et al. 2009
isopropylbenzene (cumene)	yes (15)	0.6	2	HS-SPME-GC-MS	Song et al. 2017
naphthalene	yes (13)	n. a.	0.023	HS-SPME-GC-MS	Campo et al. 2009
	yes (6)	0.025	n. a.	HS-SPME-GC-MS	Fustinoni et al. 2010
	yes (15)	0.3	1	HS-SPME-GC-MS	Song et al. 2017
phenanthrene	yes (13)	n. a.	0.005	HS-SPME-GC-MS	Campo et al. 2009
*n*‑propylbenzene	yes (15)	0.6	2	HS-SPME-GC-MS	Song et al. 2017
pyrene	yes (13)	n. a.	0.004	HS-SPME-GC-MS	Campo et al. 2009
styrene	yes (11)	n. a.	0.050	dynamic HS-GC-MS	Erb et al. 2019
toluene	yes (6)	0.034	n. a.	HS-SPME-GC-MS	Fustinoni et al. 1999
	yes (6)	0.005	n. a.	HS-SPME-GC-MS	Andreoli et al. 1999
	yes (4)	0.013	n. a.	static HS-GC-MS	Perbellini et al. 2002
	yes (6)	0.015	n. a.	PT-HS-GC-PID	Brčić Karačonji and Skender 2007
	yes (6)	0.039	n. a.	HS-SPME-GC-MS	Brčić Karačonji and Skender 2007
	yes (6)	0.015	n. a.	HS-SPME-GC-MS	Fustinoni et al. 2010
	yes (18)	1000	n. a.	static HS-GC-FID-MS	Tiscione et al. 2013
	yes (4)	1.63	5.44	HS-GC-FID	Muna and Pereira 2016
	yes (15)	0.3	1	HS-SPME-GC-MS	Song et al. 2017
	yes (2)	0.5	n. a.	HS-Cryotrapping-GC-MS	Jeong et al. 2017
	yes (5)	0.02	0.07	static HS-GC-MS	Paredes et al. 2017
	yes (5)	0.03	0.1	HS-SPME-GC-FID	Tajik et al. 2017
	yes (5)	0.03	n. a.	HS-SPME-GC-FID	Yousefi et al. 2018
	yes (11)	n. a.	0.010	dynamic HS-GC-MS	Erb et al. 2019
	yes (5)	0.35	1.18	HS-NTD-GC-FID	Saedi et al. 2020
*m*‑xylene	yes (4)	0.013	n. a.	static HS-GC-MS	Perbellini et al. 2002
	yes (11)	n. a.	0.010	dynamic HS-GC-MS	Erb et al. 2019
(*m* + *p*)‑xylene	yes (6)	0.023	n. a.	HS-SPME-GC-MS	Fustinoni et al. 1999
	yes (6)	0.01	n. a.	HS-SPME-GC-MS	Andreoli et al. 1999
	yes (6)	0.026	n. a.	PT-HS-GC-PID	Brčić Karačonji and Skender 2007
	yes (6)	0.042	n. a.	HS-SPME-GC-MS	Brčić Karačonji and Skender 2007
	yes (6)	0.015	n. a.	HS-SPME-GC-MS	Fustinoni et al. 2010
	yes (15)	0.3	1	HS-SPME-GC-MS	Song et al. 2017
	yes (5)	0.05	n. a.	HS-SPME-GC-FID	Yousefi et al. 2018
	yes (5)	0.10	0.32	HS-NTD-GC-FID	Saedi et al. 2020
*o*‑xylene	yes (6)	0.015	n. a.	HS-SPME-GC-MS	Fustinoni et al. 1999
	yes (6)	0.01	n. a.	HS-SPME-GC-MS	Andreoli et al. 1999
	yes (6)	0.030	n. a.	PT-HS-GC-PID	Brčić Karačonji and Skender 2007
	yes (6)	0.042	n. a.	HS-SPME-GC-MS	Brčić Karačonji and Skender 2007
	yes (6)	0.015	n. a.	HS-SPME-GC-MS	Fustinoni et al. 2010
	yes (15)	0.3	1	HS-SPME-GC-MS	Song et al. 2017
	yes (5)	0.07	0.2	HS-SPME-GC-FID	Tajik et al. 2017
	yes (5)	0.05	n. a.	HS-SPME-GC-FID	Yousefi et al. 2018
	yes (11)	n. a.	0.010	dynamic HS-GC-MS	Erb et al. 2019
	yes (5)	0.55	1.84	HS-NTD-GC-FID	Saedi et al. 2020
*p*‑xylene	yes (5)	0.01	0.05	static HS-GC-MS	Paredes et al. 2017
	yes (5)	0.05	0.2	HS-SPME-GC-FID	Tajik et al. 2017
	yes (11)	n. a.	0.015	dynamic HS-GC-MS	Erb et al. 2019
**aliphatic hydrocarbons**					
1,3‑butadiene	yes (3)	0.001	n. a.	static HS-GC-MS	Perbellini et al. 2003
**halogenated hydrocarbons**					
1‑bromopropane	yes (2)	2.0	n. a.	static HS-GC-ECD	B’Hymer and Cheever 2005
2‑bromopropane	yes (2)	7.0	n. a.	static HS-GC-ECD	B’Hymer and Cheever 2005
chlorodifluoromethane (Freon‑22)	yes (18)	5000	n. a.	static HS-GC-FID-MS	Tiscione et al. 2013
chloroethane	yes (18)	1900	n. a.	static HS-GC-FID-MS	Tiscione et al. 2013
dibromochloromethane	yes (6)	0.001	n. a.	TLHS-DAI-GC-ECD	Polkowska et al. 1999
dichlorodifluoromethane (Freon‑12)	yes (18)	5000	n. a.	static HS-GC-FID-MS	Tiscione et al. 2013
dichlorofluoromethane (Freon‑21)	yes (18)	5000	n. a.	static HS-GC-FID-MS	Tiscione et al. 2013
di­chloro­methane (methylene chloride)	yes (6)	0.001	n. a.	TLHS-DAI-GC-ECD	Polkowska et al. 1999
	yes (3)	0.005	n. a.	HS-SPME-GC-MS	Poli et al. 2005
	yes (4)	25.75	85.83	HS-GC-FID	Muna and Pereira 2016
	yes (11)	n. a.	0.015	dynamic HS-GC-MS	Erb et al. 2019
1,2‑di­chloro­tetra­fluoro­ethane (Freon‑114)	yes (18)	5000	n. a.	static HS-GC-FID-MS	Tiscione et al. 2013
1,1‑difluoroethane	yes (18)	< 2600	n. a.	static HS-GC-FID-MS	Tiscione et al. 2013
dimethyl disulfide	yes (5)	0.48	1.43	static HS-GC-MS	Paredes et al. 2017
fluorotri­chloromethane (Freon‑11)	yes (18)	5000	n. a.	static HS-GC-FID-MS	Tiscione et al. 2013
tetra­chloro­ethene	yes (6)	0.001	n. a.	TLHS-DAI-GC-ECD	Polkowska et al. 1999
	yes (3)	0.005	n. a.	HS-SPME-GC-MS	Poli et al. 2005
	yes (11)	n. a.	0.010	dynamic HS-GC-MS	Erb et al. 2019
tetra­chloro­methane (carbon tetrachloride)	yes (6)	0.001	n. a.	TLHS-DAI-GC-ECD	Polkowska et al. 1999
1,1,1,2‑tetrafluoroethane	yes (18)	20 000	n. a.	static HS-GC-FID-MS	Tiscione et al. 2013
tribromomethane (bromoform)	yes (6)	0.001	n. a.	TLHS-DAI-GC-ECD	Polkowska et al. 1999
tri­chloro­acetic acid	–	n. a.	9.0	PT-HS-GC-MS	Johns et al. 2005
	–	n. a.	110	HS-GC-TCD	Xie et al. 2018
	–	n. a.	172	HS-GC-FID	Xie et al. 2018
tri­chloro­ethene	yes (3)	0.005	n. a.	HS-SPME-GC-MS	Poli et al. 2005
	yes (11)	n. a.	0.010	dynamic HS-GC-MS	Erb et al. 2019
tri­chloromethane (chloroform)	yes (6)	0.001	n. a.	TLHS-DAI-GC-ECD	Polkowska et al. 1999
	yes (11)	n. a.	0.010	dynamic HS-GC-MS	Erb et al. 2019
1,1,1‑tri­fluoro­ethane (Freon‑143a)	yes (18)	3400	n. a.	static HS-GC-FID-MS	Tiscione et al. 2013
**alcohols, aldehydes, ketones, and ethers**					
acetaldehyde	yes (7)	15 667	47 000	HS-GC-FID	Kovatsi et al. 2011
	yes (18)	18 750	n. a.	static HS-GC-FID-MS	Tiscione et al. 2013
	yes (12)	0.002	n. a.	static HS-GC-MS	Serrano et al. 2016
acetone	yes (7)	24 333	73 000	HS-GC-FID	Kovatsi et al. 2011
	yes (18)	25 000	n. a.	static HS-GC-FID-MS	Tiscione et al. 2013
*tert*‑amyl methyl ether	yes (3)	0.006	n. a.	HS-SPME-GC-MS	Scibetta et al. 2007
benzaldehyde	yes (44)	0.013	0.042	HS-SPME-GC-IT/MS	Calejo et al. 2016
butanal	yes (44)	0.835	2.78	HS-SPME-GC-IT/MS	Calejo et al. 2016
	yes (12)	0.003	n. a.	static HS-GC-MS	Serrano et al. 2016
2,3‑butanedione (diacetyl)	yes (44)	0.263	0.878	HS-SPME-GC-IT/MS	Calejo et al. 2016
1‑butanol	yes (18)	25 000	n. a.	static HS-GC-FID-MS	Tiscione et al. 2013
2‑butanone (methyl ethyl ketone)	yes (44)	0.801	2.67	HS-SPME-GC-IT/MS	Calejo et al. 2016
	–	4.2	21.6	HS-SPME-GC-FID	Chou et al. 1999
	yes (18)	5000	n. a.	static HS-GC-FID-MS	Tiscione et al. 2013
butenal (crotonaldehyde)	yes (44)	0.013	0.043	HS-SPME-GC-IT/MS	Calejo et al. 2016
	yes (12)	0.003	n. a.	static HS-GC-MS	Serrano et al. 2016
cyclohexanone	yes (44)	0.137	0.455	HS-SPME-GC-IT/MS	Calejo et al. 2016
*trans*,*trans*‑2,4‑decadienal	yes (44)	0.046	0.152	HS-SPME-GC-IT/MS	Calejo et al. 2016
decanal	yes (44)	0.011	0.036	HS-SPME-GC-IT/MS	Calejo et al. 2016
2‑decanone	yes (44)	0.245	0.815	HS-SPME-GC-IT/MS	Calejo et al. 2016
*trans*‑2‑decenal	yes (44)	0.014	0.046	HS-SPME-GC-IT/MS	Calejo et al. 2016
2,6‑dimethyl-7‑octen-2‑ol (dihydomyrcenol)	yes (5)	0.03	0.08	static HS-GC-MS	Paredes et al. 2017
ethanol	yes (7)	21 667	65 000	HS-GC-FID	Kovatsi et al. 2011
	yes (2)	210	n. a.	HS-Cryotrapping-GC-MS	Jeong et al. 2017
ethyl *tert*‑butyl ether	yes (3)	0.006	n. a.	HS-SPME-GC-MS	Scibetta et al. 2007
	yes (6)	0.015	n. a.	HS-SPME-GC-MS	Fustinoni et al. 2010
formaldehyde	yes (12)	0.001	n. a.	static HS-GC-MS	Serrano et al. 2016
glyoxal	yes (44)	0.068	0.226	HS-SPME-GC-IT/MS	Calejo et al. 2016
	yes (12)	0.015	n. a.	static HS-GC-MS	Serrano et al. 2016
heptanal	yes (44)	0.010	0.034	HS-SPME-GC-IT/MS	Calejo et al. 2016
	yes (12)	0.008	n. a.	static HS-GC-MS	Serrano et al. 2016
	yes (2)	0.01	n. a.	HS-SPME-GC-FID	Ghaedrahmati et al. 2021
4‑heptanone	yes (44)	0.942	3.14	HS-SPME-GC-IT/MS	Calejo et al. 2016
*trans*‑2‑heptenal	yes (44)	0.012	0.040	HS-SPME-GC-IT/MS	Calejo et al. 2016
*trans*,*trans*‑2,4‑hexadienal	yes (44)	0.012	0.039	HS-SPME-GC-IT/MS	Calejo et al. 2016
hexanal	yes (44)	0.065	0.217	HS-SPME-GC-IT/MS	Calejo et al. 2016
	yes (12)	0.006	n. a.	static HS-GC-MS	Serrano et al. 2016
	yes (2)	0.001	n. a.	HS-SPME-GC-FID	Ghaedrahmati et al. 2021
2,5‑hexanedione	–	25	75	HS-SPME-GC-FID	Oliveira et al. 2009
2‑hexanone	yes (44)	0.017	0.055	HS-SPME-GC-IT/MS	Calejo et al. 2016
*trans*‑2‑hexenal	yes (44)	0.011	0.035	HS-SPME-GC-IT/MS	Calejo et al. 2016
4‑hydroxy‑2‑nonenal	yes (44)	15.0	50.0	HS-SPME-GC-IT/MS	Calejo et al. 2016
isobutanol (2‑methyl‑1‑propanol)	yes (18)	50 000	n. a.	static HS-GC-FID-MS	Tiscione et al. 2013
malondialdehyde	yes (44)	0.025	0.083	HS-SPME-GC-IT/MS	Calejo et al. 2016
	yes (12)	0.010	n. a.	static HS-GC-MS	Serrano et al. 2016
methanol	yes (7)	29 000	87 000	HS-GC-FID	Kovatsi et al. 2011
	yes (18)	250 000	n. a.	static HS-GC-FID-MS	Tiscione et al. 2013
1‑methoxy‑2‑propanol	–	100	n. a.	static HS-GC-FID	Tomicic and Berode 2010
2‑methylbutanal	yes (44)	0.020	0.065	HS-SPME-GC-IT/MS	Calejo et al. 2016
3‑methylbutanal	yes (44)	0.019	0.063	HS-SPME-GC-IT/MS	Calejo et al. 2016
3‑methyl‑1‑butanol (isopentanol)	yes (18)	25 000	n. a.	static HS-GC-FID-MS	Tiscione et al. 2013
methyl *tert*‑butyl ether (2-methoxy-2-methylpropane)	yes (3)	0.006	n. a.	HS-SPME-GC-MS	Scibetta et al. 2007
	yes (6)	0.010	n. a.	HS-SPME-GC-MS	Fustinoni et al. 2010
methylglyoxal	yes (44)	0.025	0.083	HS-SPME-GC-IT/MS	Calejo et al. 2016
	yes (12)	0.010	n. a.	static HS-GC-MS	Serrano et al. 2016
6‑methyl‑5‑heptanone	yes (44)	0.212	0.708	HS-SPME-GC-IT/MS	Calejo et al. 2016
4‑methyl­pentan‑2‑one (methyl isobutyl ketone)	yes (4)	68.86	229.54	HS-GC-FID	Muna and Pereira 2016
2‑methylpropanal (isobutanal)	yes (44)	0.038	0.125	HS-SPME-GC-IT/MS	Calejo et al. 2016
2‑methylpropenal	yes (44)	0.199	0.663	HS-SPME-GC-IT/MS	Calejo et al. 2016
*trans*,*trans*‑2,4‑nonadienal	yes (44)	0.010	0.034	HS-SPME-GC-IT/MS	Calejo et al. 2016
nonanal	yes (44)	0.020	0.065	HS-SPME-GC-IT/MS	Calejo et al. 2016
2‑nonanone	yes (44)	0.039	0.129	HS-SPME-GC-IT/MS	Calejo et al. 2016
*trans*‑2‑nonenal	yes (44)	0.020	0.067	HS-SPME-GC-IT/MS	Calejo et al. 2016
octanal	yes (44)	0.152	0.507	HS-SPME-GC-IT/MS	Calejo et al. 2016
2‑octanone (methyl hexyl ketone)	yes (44)	0.107	0.355	HS-SPME-GC-IT/MS	Calejo et al. 2016
	yes (5)	0.06	0.17	static HS-GC-MS	Paredes et al. 2017
*trans*‑2‑octenal	yes (44)	0.022	0.072	HS-SPME-GC-IT/MS	Calejo et al. 2016
pentanal	yes (44)	0.273	0.909	HS-SPME-GC-IT/MS	Calejo et al. 2016
	yes (12)	0.006	n. a.	static HS-GC-MS	Serrano et al. 2016
2‑pentanone	yes (44)	0.013	0.043	HS-SPME-GC-IT/MS	Calejo et al. 2016
*trans*‑2‑pentenal	yes (44)	0.040	0.133	HS-SPME-GC-IT/MS	Calejo et al. 2016
3‑penten‑2‑one	yes (44)	0.498	1.66	HS-SPME-GC-IT/MS	Calejo et al. 2016
phenylacetaldehyde	yes (44)	0.009	0.029	HS-SPME-GC-IT/MS	Calejo et al. 2016
propanal	yes (44)	0.016	0.052	HS-SPME-GC-IT/MS	Calejo et al. 2016
	yes (12)	0.004	n. a.	static HS-GC-MS	Serrano et al. 2016
1‑propanol	yes (7)	26 000	78 000	HS-GC-FID	Kovatsi et al. 2011
2‑propanol (isopropanol)	yes (18)	100 000	n. a.	static HS-GC-FID-MS	Tiscione et al. 2013
2-propenal (acrolein)	yes (44)	0.030	0.091	HS-SPME-GC-IT/MS	Calejo et al. 2016
	yes (12)	0.003	n. a.	static HS-GC-MS	Serrano et al. 2016
undecanal	yes (44)	0.011	0.035	HS-SPME-GC-IT/MS	Calejo et al. 2016
2‑undecanone	yes (44)	0.074	0.247	HS-SPME-GC-IT/MS	Calejo et al. 2016
**inhalational anaesthetics**					
bromomethane as a metabolite of halothane	yes (2)	2876–8789	n. a.	static HS-GC-FID	Maiorino et al. 1980
desflurane	yes (7)	13 667	41 000	HS-GC-FID	Kovatsi et al. 2011
halothane (2-bromo-2-chloro-1,1,1-tri­fluoro­ethane)	yes (3)	0.02–0.03	n. a.	HS-SPME-GC-MS	Poli et al. 1999
	yes (3)	5	n. a.	static HS- GC-MS	Poli et al. 1999
	yes (4)	0.05	0.15	static HS-GC-MS	Accorsi et al. 2001
	–	≈ 4	≈ 50	HS-SPME-GC-MS	Musshoff et al. 2000
hexafluoroisopropanol as a metabolite of sevoflurane	–	≈ 1	n. a.	HSSE-GC-MS	Accorsi et al. 2005
	–	n. a.	0.5	HS-GC-MS	Herzog-Niescery et al. 2020
isoflurane	yes (3)	0.15–0.02	n. a.	HS-SPME-GC-MS	Poli et al. 1999
	yes (3)	1	n. a.	static HS-GC-MS	Poli et al. 1999
	yes (4)	0.02	0.08	static HS-GC-MS	Accorsi et al. 2001
laughing gas (dinitrogen oxide)	yes (3)	0.075–0.1	n. a.	HS-SPME-GC-MS	Poli et al. 1999
	yes (3)	1	n. a.	static HS-GC-MS	Poli et al. 1999
	yes (4)	0.3	1.0	static HS-GC-MS	Accorsi et al. 2001
sevoflurane	yes (4)	0.03	0.10	static HS-GC-MS	Accorsi et al. 2001
	–	≈ 1	n. a.	HSSE-GC-MS	Accorsi et al. 2005
	yes (7)	13 667	41 000	HS-GC-FID	Kovatsi et al. 2011
trifluoroacetic acid as a metabolite of halothane, isoflurane, and fluroxene	yes (2)	1140	n. a.	static HS-GC-FID	Maiorino et al. 1980
**others**					
2,5‑dimethylfuran	yes (3)	0.005	n. a.	static HS-GC-MS	Perbellini et al. 2003
2‑furfural	yes (44)	0.044	0.147	HS-SPME-GC-IT/MS	Calejo et al. 2016
menthol	–	1.7	n. a.	HS-SPME-GC-MS	Huang et al. 2017
5‑methyl‑2‑furfural	yes (44)	0.025	0.083	HS-SPME-GC-IT/MS	Calejo et al. 2016
tetra­hydro­furan	yes (4)	155.12	517.07	HS-GC-FID	Muna and Pereira 2016

For abbreviations, see List of abbreviations.

**Tab.6:** Further internationally published headspace methods for the matrices blood, serum and plasma

Analyte (synonym)	Multimethod (number of analytes)	Detection limit [μg/l]	Quantitation limit [μg/l]	Analytical method	References
**aromatic hydrocarbons**					
benzene	yes (6)	0.005	n. a.	HS-SPME-GC-MS	Andreoli et al. 1999
	yes (20)	n. a.	≈ 10	HS-SPME-GC-MS	Liu et al. 2000
	yes (4)	0.016	n. a.	static HS-GC-MS	Perbellini et al. 2002
	yes (3)	0.010	n. a.	static HS-GC-MS	Perbellini et al. 2003
	yes (31)	0.024	n. a.	HS-SPME-GC-MS	Blount et al. 2006
	yes (10)	0.4	1.2	HS-NTD-GC-MS	Alonso et al. 2012
	yes (70)	0.001	0.004	HS-SPME-GC-MS	Mochalski et al. 2013
	yes (24)	n. a.	7.21–10.6	HS-SPME-GC-MS	Waters et al. 2017
*n*‑butylbenzene	yes (24)	n. a.	7.21–10.6	HS-SPME-GC-MS	Waters et al. 2017
*tert*‑butylbenzene	yes (24)	n. a.	7.21–10.6	HS-SPME-GC-MS	Waters et al. 2017
chlorobenzene	yes (31)	0.011	n. a.	HS-SPME-GC-MS	Blount et al. 2006
1,2‑dichlorobenzene	yes (31)	0.100	n. a.	HS-SPME-GC-MS	Blount et al. 2006
	yes (10)	0.25	1.4	HS-NTD-GC-MS	Alonso et al. 2012
1,3‑dichlorobenzene	yes (31)	0.050	n. a.	HS-SPME-GC-MS	Blount et al. 2006
1,4‑dichlorobenzene	yes (31)	0.120	n. a.	HS-SPME-GC-MS	Blount et al. 2006
ethylbenzene	yes (6)	0.01	n. a.	HS-SPME-GC-MS	Andreoli et al. 1999
	yes (20)	n. a.	≈ 10	HS-SPME-GC-MS	Liu et al. 2000
	yes (4)	0.022	n. a.	static HS-GC-MS	Perbellini et al. 2002
	yes (31)	0.024	n. a.	HS-SPME-GC-MS	Blount et al. 2006
	yes (10)	0.2	n. a.	HS-NTD-GC-MS	Alonso et al. 2012
	yes (70)	0.042	0.127	HS-SPME-GC-MS	Mochalski et al. 2013
	yes (24)	n. a.	7.21–10.6	HS-SPME-GC-MS	Waters et al. 2017
2‑ethyltoluene	yes (20)	n. a.	≈ 10	HS-SPME-GC-MS	Liu et al. 2000
	yes (24)	n. a.	7.21–10.6	HS-SPME-GC-MS	Waters et al. 2017
3‑ethyltoluene	yes (20)	n. a.	≈ 10	HS-SPME-GC-MS	Liu et al. 2000
	yes (24)	n. a.	7.21–10.6	HS-SPME-GC-MS	Waters et al. 2017
indene	yes (24)	n. a.	7.21–10.6	HS-SPME-GC-MS	Waters et al. 2017
isopropylbenzene (cumene)	yes (20)	n. a.	≈ 10	HS-SPME-GC-MS	Liu et al. 2000
	yes (24)	n. a.	7.21–10.6	HS-SPME-GC-MS	Waters et al. 2017
4-isopropyltoluene (*p*‑cymene)	yes (70)	0.013	0.040	HS-SPME-GC-MS	Mochalski et al. 2013
α‑methylstyrene	yes (70)	0.012	0.036	HS-SPME-GC-MS	Mochalski et al. 2013
naphthalene	yes (24)	n. a.	7.21–10.6	HS-SPME-GC-MS	Waters et al. 2017
*n‑*propylbenzene	yes (20)	n. a.	≈ 10	HS-SPME-GC-MS	Liu et al. 2000
	yes (24)	n. a.	7.21–10.6	HS-SPME-GC-MS	Waters et al. 2017
styrene	yes (31)	0.050	n. a.	HS-SPME-GC-MS	Blount et al. 2006
	yes (10)	0.1	1.4	HS-NTD-GC-MS	Alonso et al. 2012
	yes (70)	0.010	0.031	HS-SPME-GC-MS	Mochalski et al. 2013
	yes (24)	n. a.	7.21–10.6	HS-SPME-GC-MS	Waters et al. 2017
toluene	yes (6)	0.005	n. a.	HS-SPME-GC-MS	Andreoli et al. 1999
	yes (20)	n. a.	≈ 10	HS-SPME-GC-MS	Liu et al. 2000
	yes (4)	0.043	n. a.	static HS-GC-MS	Perbellini et al. 2002
	yes (31)	0.025	n. a.	HS-SPME-GC-MS	Blount et al. 2006
	yes (10)	0.2	1.4	HS-NTD-GC-MS	Alonso et al. 2012
	yes (70)	0.003	0.008	HS-SPME-GC-MS	Mochalski et al. 2013
	yes (18)	1000	n. a.	static HS-GC-FID-MS	Tiscione et al. 2013
	yes (24)	n. a.	7.21–10.6	HS-SPME-GC-MS	Waters et al. 2017
1,2,3‑tri­methyl­benzene (hemimellitene)	yes (20)	n. a.	≈ 10	HS-SPME-GC-MS	Liu et al. 2000
	yes (24)	n. a.	7.21–10.6	HS-SPME-GC-MS	Waters et al. 2017
1,2,4‑tri­methyl­benzene (pseudocumene)	yes (20)	n. a.	≈ 10	HS-SPME-GC-MS	Liu et al. 2000
	yes (24)	n. a.	7.21–10.6	HS-SPME-GC-MS	Waters et al. 2017
1,3,5‑tri­methyl­benzene (mesitylene)	yes (20)	n. a.	≈ 10	HS-SPME-GC-MS	Liu et al. 2000
	yes (24)	n. a.	7.21–10.6	HS-SPME-GC-MS	Waters et al. 2017
*m‑*xylene	yes (4)	0.052	n. a.	static HS-GC-MS	Perbellini et al. 2002
(*m* + *p*)‑xylene	yes (6)	0.01	n. a.	HS-SPME-GC-MS	Andreoli et al. 1999
	yes (20)	n. a.	≈ 10	HS-SPME-GC-MS	Liu et al. 2000
	yes (31)	0.034	n. a.	HS-SPME-GC-MS	Blount et al. 2006
	yes (10)	0.3	1.3	HS-NTD-GC-MS	Alonso et al. 2012
	yes (70)	0.007	0.022	HS-SPME-GC-MS	Mochalski et al. 2013
	yes (24)	n. a.	7.21–10.6	HS-SPME-GC-MS	Waters et al. 2017
*o‑*xylene	yes (6)	0.01	n. a.	HS-SPME-GC-MS	Andreoli et al. 1999
	yes (20)	n. a.	≈ 10	HS-SPME-GC-MS	Liu et al. 2000
	yes (31)	0.024	n. a.	HS-SPME-GC-MS	Blount et al. 2006
	yes (10)	0.2	1.3	HS-NTD-GC-MS	Alonso et al. 2012
	yes (70)	0.009	0.026	HS-SPME-GC-MS	Mochalski et al. 2013
	yes (24)	n. a.	7.21–10.6	HS-SPME-GC-MS	Waters et al. 2017
**aliphatic hydrocarbons**					
1,3‑butadiene	yes (3)	0.0005	n. a.	static HS-GC-MS	Perbellini et al. 2003
	yes (70)	0.004	0.011	HS-SPME-GC-MS	Mochalski et al. 2013
*n*‑butane	yes (70)	0.008	0.023	HS-SPME-GC-MS	Mochalski et al. 2013
*n*‑decane	yes (20)	n. a.	≈ 10	HS-SPME-GC-MS	Liu et al. 2000
	yes (70)	0.043	0.128	HS-SPME-GC-MS	Mochalski et al. 2013
	yes (24)	n. a.	7.21–10.6	HS-SPME-GC-MS	Waters et al. 2017
2,3‑dimethylbutane	yes (70)	0.005	0.016	HS-SPME-GC-MS	Mochalski et al. 2013
*n*‑dodecane	yes (20)	n. a.	≈ 10	HS-SPME-GC-MS	Liu et al. 2000
	yes (24)	n. a.	7.21–10.6	HS-SPME-GC-MS	Waters et al. 2017
*n*‑heptane	yes (20)	n. a.	≈ 10	HS-SPME-GC-MS	Liu et al. 2000
	yes (24)	n. a.	7.21–10.6	HS-SPME-GC-MS	Waters et al. 2017
*cis*,*trans*‑2,4‑hexadiene	yes (70)	0.002	0.005	HS-SPME-GC-MS	Mochalski et al. 2013
*n*‑hexane	yes (20)	n. a.	≈ 10	HS-SPME-GC-MS	Liu et al. 2000
	yes (70)	0.002	0.005	HS-SPME-GC-MS	Mochalski et al. 2013
1‑hexene	yes (70)	0.002	0.005	HS-SPME-GC-MS	Mochalski et al. 2013
isoprene	yes (70)	0.003	0.008	HS-SPME-GC-MS	Mochalski et al. 2013
2‑methylbutane (isopentane)	yes (70)	0.005	0.015	HS-SPME-GC-MS	Mochalski et al. 2013
2‑methyl-1‑butene	yes (70)	0.004	0.011	HS-SPME-GC-MS	Mochalski et al. 2013
2‑methylhexane	yes (70)	0.002	0.006	HS-SPME-GC-MS	Mochalski et al. 2013
4‑methyloctane	yes (70)	0.019	0.058	HS-SPME-GC-MS	Mochalski et al. 2013
2‑methyl­pentane	yes (70)	0.007	0.021	HS-SPME-GC-MS	Mochalski et al. 2013
4‑methyl-1‑pentene	yes (70)	0.003	0.008	HS-SPME-GC-MS	Mochalski et al. 2013
2‑methylpropane (isobutane)	yes (70)	0.013	0.040	HS-SPME-GC-MS	Mochalski et al. 2013
2‑methyl-1‑propene (isobutene)	yes (70)	0.006	0.019	HS-SPME-GC-MS	Mochalski et al. 2013
*n*‑nonane	yes (20)	n. a.	≈ 10	HS-SPME-GC-MS	Liu et al. 2000
	yes (24)	n. a.	7.21–10.6	HS-SPME-GC-MS	Waters et al. 2017
*n*‑octane	yes (20)	n. a.	≈ 10	HS-SPME-GC-MS	Liu et al. 2000
	yes (70)	0.005	0.014	HS-SPME-GC-MS	Mochalski et al. 2013
	yes (24)	n. a.	7.21–10.6	HS-SPME-GC-MS	Waters et al. 2017
*cis*‑2‑pentene	yes (70)	0.003	0.008	HS-SPME-GC-MS	Mochalski et al. 2013
*trans*‑2‑pentene	yes (70)	0.003	0.008	HS-SPME-GC-MS	Mochalski et al. 2013
*cis*‑1,3‑pentadiene	yes (70)	0.001	0.004	HS-SPME-GC-MS	Mochalski et al. 2013
*trans*‑1,3‑pentadiene	yes (70)	0.002	0.006	HS-SPME-GC-MS	Mochalski et al. 2013
*n*-pentane	yes (70)	0.007	0.022	HS-SPME-GC-MS	Mochalski et al. 2013
propene (propylene)	yes (70)	0.156	0.467	HS-SPME-GC-MS	Mochalski et al. 2013
*n*‑tridecane	yes (20)	n. a.	≈ 10	HS-SPME-GC-MS	Liu et al. 2000
	yes (24)	n. a.	7.21–10.6	HS-SPME-GC-MS	Waters et al. 2017
*n*‑undecane	yes (20)	n. a.	≈ 10	HS-SPME-GC-MS	Liu et al. 2000
	yes (70)	0.109	0.328	HS-SPME-GC-MS	Mochalski et al. 2013
	yes (24)	n. a.	7.21–10.6	HS-SPME-GC-MS	Waters et al. 2017
**halogenated hydrocarbons**					
bromochloroiodomethane	yes (2)	0.002	n. a.	HS-SPME-GC-HRMS	Silva et al. 2006
bromodi­chloro­methane	yes (5)	0.0003	n. a.	HS-SPME-GC-HRMS	Bonin et al. 2005
	yes (5)	0.0004	n. a.	PT-HS-GC-HRMS	Bonin et al. 2005
	yes (31)	0.030	n. a.	HS-SPME-GC-MS	Blount et al. 2006
chlorodifluoromethane (Freon-22)	yes (18)	5000	n. a.	static HS-GC-FID-MS	Tiscione et al. 2013
chloroethane	yes (18)	1900	n. a.	static HS-GC-FID-MS	Tiscione et al. 2013
dibromochloromethane	yes (5)	0.0004	n. a.	HS-SPME-GC-HRMS	Bonin et al. 2005
	yes (5)	0.0001	n. a.	PT-HS-GC-HRMS	Bonin et al. 2005
	yes (31)	0.005	n. a.	HS-SPME-GC-MS	Blount et al. 2006
dibromomethane	yes (31)	0.030	n. a.	HS-SPME-GC-MS	Blount et al. 2006
dichlorodifluoromethan (Freon-12)	yes (18)	5000	n. a.	static HS-GC-FID-MS	Tiscione et al. 2013
1,1‑dichloroethane	yes (31)	0.010	n. a.	HS-SPME-GC-MS	Blount et al. 2006
1,2‑dichloroethane	yes (31)	0.009	n. a.	HS-SPME-GC-MS	Blount et al. 2006
1,1‑dichloroethene	yes (31)	0.009	n. a.	HS-SPME-GC-MS	Blount et al. 2006
*cis*‑1,2‑dichloroethene	yes (31)	0.010	n. a.	HS-SPME-GC-MS	Blount et al. 2006
*trans*‑1,2‑dichloroethene	yes (31)	0.009	n. a.	HS-SPME-GC-MS	Blount et al. 2006
dichlorofluoromethane (Freon-21)	yes (18)	5000	n. a.	static HS-GC-FID-MS	Tiscione et al. 2013
dichloroiodomethane	yes (2)	0.002	n. a.	HS-SPME-GC-HRMS	Silva et al. 2006
di­chloro­methane (methylene chloride)	yes (31)	0.070	n. a.	HS-SPME-GC-MS	Blount et al. 2006
1,2‑dichloropropane	yes (31)	0.008	n. a.	HS-SPME-GC-MS	Blount et al. 2006
	yes (10)	0.2	1.8	HS-NTD-GC-MS	Alonso et al. 2012
1,2‑di­chloro­tetra­fluoro­ethane (Freon-114)	yes (18)	5000	n. a.	static HS-GC-FID-MS	Tiscione et al. 2013
1,1‑difluoroethane	yes (18)	< 2600	n. a.	static HS-GC-FID-MS	Tiscione et al. 2013
fluorotri­chloromethane (Freon-11)	yes (18)	5000	n. a.	static HS-GC-FID-MS	Tiscione et al. 2013
hexachloroethane	yes (31)	0.011	n. a.	HS-SPME-GC-MS	Blount et al. 2006
1,1,2,2‑tetrachloroethane	yes (31)	0.010	n. a.	HS-SPME-GC-MS	Blount et al. 2006
tetra­chloro­ethene	yes (31)	0.048	n. a.	HS-SPME-GC-MS	Blount et al. 2006
tetra­chloro­methane (carbon tetrachloride)	yes (31)	0.005	n. a.	HS-SPME-GC-MS	Blount et al. 2006
1,1,1,2‑tetrafluoroethane	yes (18)	20 000	n. a.	static HS-GC-FID-MS	Tiscione et al. 2013
tribromomethane (bromoform)	yes (5)	0.0006	n. a.	HS-SPME-GC-HRMS	Bonin et al. 2005
	yes (5)	0.0002	n. a.	PT-HS-GC-HRMS	Bonin et al. 2005
	yes (31)	0.020	n. a.	HS-SPME-GC-MS	Blount et al. 2006
1,1,1‑tri­chloro­ethane	–	n. a.	0.8	PT-HS-GC-MS	Johns et al. 2005
	yes (31)	0.048	n.a	HS-SPME-GC-MS	Blount et al. 2006
1,1,2‑tri­chloro­ethane	yes (31)	0.010	n. a.	HS-SPME-GC-MS	Blount et al. 2006
tri­chloro­ethene	yes (31)	0.012	n. a.	HS-SPME-GC-MS	Blount et al. 2006
tri­chloromethane (chloroform)	yes (5)	0.0024	n. a.	HS-SPME-GC-HRMS	Bonin et al. 2005
	yes (5)	0.0032	n. a.	PT-HS-GC-HRMS	Bonin et al. 2005
	yes (31)	0.020	n. a.	HS-SPME-GC-MS	Blount et al. 2006
1,1,1‑tri­fluoro­ethane (Freon-143a)	yes (18)	3400	n. a.	static HS-GC-FID-MS	Tiscione et al. 2013
**alcohols, aldehydes, ketones and ethers**					
acetaldehyde	yes (7)	15 333	46 000	HS-GC-FID	Kovatsi et al. 2011
	yes (18)	18 750	n. a.	static HS-GC-FID-MS	Tiscione et al. 2013
	yes (5)	100	500	static HS-GC-MS	Cordell et al. 2013
	yes (20)	50.6 (serum)	n. a.	HS-SPME-GC-HRMS	Silva et al. 2018
acetone	yes (7)	7333	22 000	HS-GC-FID	Kovatsi et al. 2011
	yes (18)	25 000	n. a.	static HS-GC-FID-MS	Tiscione et al. 2013
	yes (5)	100	500	static HS-GC-MS	Cordell et al. 2013
*tert*‑amyl methyl ether	yes (4)	0.0006	n. a.	HS-SPME-GC-HRMS	Silva et al. 2008
benzaldehyde	yes (70)	0.265	0.796	HS-SPME-GC-MS	Mochalski et al. 2013
	yes (20)	0.461 (serum)	n. a.	HS-SPME-GC-HRMS	Silva et al. 2018
butanal	yes (20)	0.313 (serum)	n. a.	HS-SPME-GC-HRMS	Silva et al. 2018
2,3‑butanedione (dimethyl diketone)	yes (70)	0.344	1.03	HS-SPME-GC-MS	Mochalski et al. 2013
1‑butanol	yes (18)	25 000	n. a.	static HS-GC-FID-MS	Tiscione et al. 2013
*tert*‑butanol	yes (2)	0.05 (serum)	0.15 (serum)	HS-SPME-GC-MS	Zhang et al. 2015
2‑butanone (methyl ethyl ketone)	yes (70)	0.029	0.087	HS-SPME-GC-MS	Mochalski et al. 2013
	yes (18)	5000	n. a.	static HS-GC-FID-MS	Tiscione et al. 2013
crotonaldehyde	yes (20)	0.147 (serum)	n. a.	HS-SPME-GC-HRMS	Silva et al. 2018
decanal	yes (20)	3.90 (serum)	n. a.	HS-SPME-GC-HRMS	Silva et al. 2018
diisopropyl ether	yes (4)	0.0006	n. a.	HS-SPME-GC-HRMS	Silva et al. 2008
ethanol	yes (7)	15 667	47 000	HS-GC-FID	Kovatsi et al. 2011
	yes (5)	100	500	static HS-GC-MS	Cordell et al. 2013
ethyl acetate	yes (70)	0.009	0.026	HS-SPME-GC-MS	Mochalski et al. 2013
ethyl *tert‑*butyl ether	yes (4)	0.0006	n. a.	HS-SPME-GC-HRMS	Silva et al. 2008
ethyl vinyl ether	yes (70)	0.003	0.009	HS-SPME-GC-MS	Mochalski et al. 2013
furfural (2-furaldehyde)	yes (20)	1.24 (serum)	n. a.	HS-SPME-GC-HRMS	Silva et al. 2018
heptanal	yes (20)	0.312 (serum)	n. a.	HS-SPME-GC-HRMS	Silva et al. 2018
	yes (2)	0.01 (plasma)	n. a.	HS-SPME-GC-FID	Ghaedrahmati et al. 2021
2‑heptanone	yes (70)	0.023	0.069	HS-SPME-GC-MS	Mochalski et al. 2013
4‑heptanone	yes (70)	0.006	0.017	HS-SPME-GC-MS	Mochalski et al. 2013
hexanal	yes (20)	0.693 (serum)	n. a.	HS-SPME-GC-HRMS	Silva et al. 2018
	yes (2)	0.001 (plasma)	n. a.	HS-SPME-GC-FID	Ghaedrahmati et al. 2021
2‑hexanone	yes (70)	0.015	0.045	HS-SPME-GC-MS	Mochalski et al. 2013
3‑hexanone	yes (70)	0.015	0.045	HS-SPME-GC-MS	Mochalski et al. 2013
*trans*‑2‑hexenal	yes (20)	0.290 (serum)	n. a.	HS-SPME-GC-HRMS	Silva et al. 2018
isobutanol (2‑methyl-1-propanol)	yes (18)	50 000	n. a.	static HS-GC-FID-MS	Tiscione et al. 2013
Isopentanal (isovaleraldehyde)	yes (20)	0.119 (serum)	n. a.	HS-SPME-GC-HRMS	Silva et al. 2018
methanol	yes (7)	15 000	45 000	HS-GC-FID	Kovatsi et al. 2011
	yes (5)	200	1000	static HS-GC-MS	Cordell et al. 2013
	yes (18)	250 000	n. a.	static HS-GC-FID-MS	Tiscione et al. 2013
methyl acetate	yes (70)	0.074	0.222	HS-SPME-GC-MS	Mochalski et al. 2013
2-methylbenzaldehyde (*o*‑tolualdehyde)	yes (20)	0.142 (serum)	n. a.	HS-SPME-GC-HRMS	Silva et al. 2018
3‑methyl-1‑butanol (isopentanol)	yes (18)	25 000	n. a.	static HS-GC-FID-MS	Tiscione et al. 2013
methyl *tert*‑butyl ether (2-methoxy-2-methylpropane)	yes (5)	0.0015	n. a.	HS-SPME-GC-HRMS	Bonin et al. 2005
	yes (5)	0.0045	n. a.	PT-HS-GC-HRMS	Bonin et al. 2005
	yes (31)	0.100	n. a.	HS-SPME-GC-MS	Blount et al. 2006
	yes (4)	0.0006	n. a.	HS-SPME-GC-HRMS	Silva et al. 2008
	yes (2)	0.03 (serum)	0.09 (serum)	HS-SPME-GC-MS	Zhang et al. 2015
2-methyl-1-propanal (isobutanal)	yes (20)	0.109 (serum)	n. a.	HS-SPME-GC-HRMS	Silva et al. 2018
2‑methyl-2-propenal	yes (70)	0.063	0.189	HS-SPME-GC-MS	Mochalski et al. 2013
methyl propionate	yes (70)	0.012	0.034	HS-SPME-GC-MS	Mochalski et al. 2013
methyl vinyl ketone (3-buten-2-one)	yes (70)	2.80	8.41	HS-SPME-GC-MS	Mochalski et al. 2013
nonanal	yes (20)	2.63 (serum)	n. a.	HS-SPME-GC-HRMS	Silva et al. 2018
*trans*‑2‑nonenal	yes (20)	2.68 (serum)	n. a.	HS-SPME-GC-HRMS	Silva et al. 2018
octanal	yes (20)	0.660 (serum)	n. a.	HS-SPME-GC-HRMS	Silva et al. 2018
*trans*‑2‑octenal	yes (20)	1.12 (serum)	n. a.	HS-SPME-GC-HRMS	Silva et al. 2018
pentanal	yes (20)	0.316 (serum)	n. a.	HS-SPME-GC-HRMS	Silva et al. 2018
2‑pentanone	yes (70)	0.022	0.065	HS-SPME-GC-MS	Mochalski et al. 2013
*trans*‑3‑penten‑2‑one	yes (70)	0.210	0.631	HS-SPME-GC-MS	Mochalski et al. 2013
propanal	yes (70)	0.076	0.227	HS-SPME-GC-MS	Mochalski et al. 2013
	yes (20)	1.16 (serum)	n. a.	HS-SPME-GC-HRMS	Silva et al. 2018
2‑propenal (acrolein)	yes (70)	15.1	45.4	HS-SPME-GC-MS	Mochalski et al. 2013
	yes (20)	2.16 (serum)	n. a.	HS-SPME-GC-HRMS	Silva et al. 2018
1‑propanol	yes (7)	8333	25 000	HS-GC-FID	Kovatsi et al. 2011
2‑propanol (isopropanol)	yes (18)	100 000	n. a.	static HS-GC-FID-MS	Tiscione et al. 2013
**inhalational anaesthetics**					
bromomethane as a metabolite of halothane	yes (2)	3995–6392 (plasma)	n. a.	static HS-GC-FID	Maiorino et al. 1980
desflurane	yes (7)	11 333	34 000	HS-GC-FID	Kovatsi et al. 2011
	–	n. a.	n. a.	HS-GC-MS/MS	Tamura et al. 2020
halothane (2‑bromo-2‑chloro-1,1,1‑tri­fluoro­ethane) with enflurane as ISTD	–	≈ 4	≈ 50	HS-SPME-GC-MS	Musshoff et al. 2000
sevoflurane	yes (7)	17 333	52 000	HS-GC-FID	Kovatsi et al. 2011
	–	n. a.	n. a.	HS-GC-FID	Lin et al. 2015
	–	n. a.	n. a.	HS-GC-MS/MS	Tamura et al. 2020
trifluoroacetic acid as a metabolite of halothane, isoflurane, and fluroxene	yes (2)	285 (plasma)	n. a.	static HS-GC-FID	Maiorino et al. 1980
**others**					
acetic acid	yes (5)	100	500	static HS-GC-MS	Cordell et al. 2013
acetonitrile	yes (70)	0.608	1.82	HS-SPME-GC-MS	Mochalski et al. 2013
allyl methyl sulfide	yes (70)	0.003	0.008	HS-SPME-GC-MS	Mochalski et al. 2013
3‑carene	yes (70)	0.123	0.368	HS-SPME-GC-MS	Mochalski et al. 2013
1,8‑cineole (eucalyptol)	yes (70)	0.123	0.370	HS-SPME-GC-MS	Mochalski et al. 2013
2,5‑dimethylfuran	yes (3)	0.005	n. a.	static HS-GC-MS	Perbellini et al. 2003
	yes (31)	0.012	n. a.	HS-SPME-GC-MS	Blount et al. 2006
	yes (10)	0.1	1.4	HS-NTD-GC-MS	Alonso et al. 2012
	yes (70)	0.002	0.007	HS-SPME-GC-MS	Mochalski et al. 2013
	yes (20)	0.038 (serum)	n. a.	HS-SPME-GC-HRMS	Silva et al. 2018
dimethyl selenide	yes (70)	0.003	0.010	HS-SPME-GC-MS	Mochalski et al. 2013
dimethyl sulfide	yes (70)	0.006	0.019	HS-SPME-GC-MS	Mochalski et al. 2013
ethyl methyl sulfide	yes (70)	0.005	0.014	HS-SPME-GC-MS	Mochalski et al. 2013
furan	yes (10)	0.2	1.0	HS-NTD-GC-MS	Alonso et al. 2012
	yes (70)	0.001	0.003	HS-SPME-GC-MS	Mochalski et al. 2013
hydrogen cyanide	–	13.8	n. a.	static HS-GC-NPD	Calafat and Stanfill 2002
limonene (1-methyl‑4‑(1‑methylvinyl)cyclohexene)	yes (70)	0.011	0.033	HS-SPME-GC-MS	Mochalski et al. 2013
menthone	yes (70)	0.093	0.278	HS-SPME-GC-MS	Mochalski et al. 2013
2‑methylfuran	yes (70)	0.001	0.003	HS-SPME-GC-MS	Mochalski et al. 2013
3‑methylfuran	yes (70)	0.001	0.004	HS-SPME-GC-MS	Mochalski et al. 2013
methyl propyl sulfide	yes (70)	0.004	0.011	HS-SPME-GC-MS	Mochalski et al. 2013
1‑methylpyrrole	yes (70)	0.008	0.024	HS-SPME-GC-MS	Mochalski et al. 2013
3‑methylthiophene	yes (70)	0.002	0.006	HS-SPME-GC-MS	Mochalski et al. 2013
*α*‑pinene	yes (70)	0.008	0.025	HS-SPME-GC-MS	Mochalski et al. 2013
*β*‑pinene	yes (70)	0.005	0.016	HS-SPME-GC-MS	Mochalski et al. 2013
pyrazine	yes (70)	0.360	1.08	HS-SPME-GC-MS	Mochalski et al. 2013
pyrrole	yes (70)	0.001	0.003	HS-SPME-GC-MS	Mochalski et al. 2013
*γ*-terpinene	yes (70)	0.136	0.409	HS-SPME-GC-MS	Mochalski et al. 2013
thiophene (thiofuran)	yes (70)	0.001	0.003	HS-SPME-GC-MS	Mochalski et al. 2013

For abbreviations, see List of abbreviations.

**Tab.7:** Assessment values for parameters which can be measured by headspace methods

Substance (synonym)	Analyte	Matrix	Sampling time	Limit-value category	Value	Committee, Country	References
acetone	acetone	urine	end of exposure or end of shift	BAR	2.5 mg/l	MAK Commission, Germany	DFG 2025
				BAT	50 mg/l		
				BGW	50 mg/l	AGS, Germany	AGS 2013
				BAT-Suva	50 mg/l	Suva, Switzerland	Koller et al. 2018; SUVA 2025 a, b
			within 2 hours before the end of shift	OEL-B	40 mg/l	JSOH, Japan	JSOH 2023
			end of shift	BEI^®^	25 mg/l	BEI Committee, USA	ACGIH 2025
benzene	benzene	blood	before the shift at the end of the workweek	BAL	1.6 μg/l	FIOH, Finnland	Kiilunen 1999
			end of exposure	BLV	28 μg/l	SCOEL, EU Commission	SCOEL 2006
		urine	end of exposure or end of shift	BAR	0.3 μg/l^a)^	MAK Commission, Germany	DFG 2025
				EKA	0.5–12.5 μg/l		
				Equi­valence value to the tol­er­ance con­centra­tion for carcinogenic substances	5 μg/l	AGS, Germany	AGS 2014
				Equivalence value to the acceptance concentration for carcinogenic substances	0.8 μg/l^a)^		
				BGV	0.3 μg/l	RAC, EU Commission	RAC 2018
				BLV	0.7 μg/l		
1-butanol	1-butanol	urine	end of exposure or end of shift	BAT	10 mg/g creatinine	MAK Commission, Germany	DFG 2025
				BAT-Suva	10 mg/g creatinine	Suva, Switzerland	Koller et al. 2018; SUVA 2025 a, b
				BGV	10 mg/g creatinine^b)^	AGS, Germany	AGS 2013
			before the next shift	BAT	2 mg/g creatinine	MAK Commission, Germany	DFG 2025
				BGV	2 mg/g creatinine^b)^	AGS, Germany	AGS 2013
			before the next shift or 16 h after end of exposure	BAT-Suva	2 mg/g creatinine	Suva, Switzerland	Koller et al. 2018; SUVA 2025 a, b
2-butanone (methyl ethyl ketone)	2-butanone	urine	end of exposure or end of shift	BAT	2 mg/l	MAK Commission, Germany	DFG 2025
				BGV	2 mg/l	AGS, Germany	AGS 2013
			end of exposure or end of shift, before the next shift or 16 h after end of exposure	BAT-Suva	2 mg/l	Suva, Switzerland	Koller et al. 2018; SUVA 2025 a, b
			end of shift	BEI^®^	2 mg/l	BEI Committee, USA	ACGIH 2025
				BLV	5.0 mg/l	SCOEL, EU Commission	SCOEL 1999
				BMGV	70 μmol/l (5 mg/l)	HSE, United Kingdom	HSE 2020, 2025
			end of shift or after several hours in cases of high exposure levels	OEL-B	5 mg/l	JSOH, Japan	JSOH 2023
			end of shift at the end of the working week	BAL	4.3 mg/l	FIOH, Finnland	Kiilunen 1999
carbon monoxide	CO-Hb	blood	end of exposure or end of shift	BAT	5%	MAK Commission, Germany	DFG 2025
				BAL	4%	FIOH, Finnland	Kiilunen 1999
			end of shift	BEI^®^	3.5%	BEI Committee, USA	ACGIH 2025
	CO	exhaled air	end of shift	BEI^®^	20 ppm	BEI Committee, USA	ACGIH 2025
cyclohexanone	cyclohexanol	urine	end of shift	BMGV	2 mmol/mol creatinine	HSE, United Kingdom	HSE 2020, 2025
				BEI^®^	8 mg/l	BEI Committee, USA	ACGIH 2025
di­chloro­methane (methylene chloride)	di­chloro­methane	blood	immediately after exposure	EKA	100–1000 μg/l	MAK Commission, Germany	DFG 2025
				BAT	500 μg/l		
				BGW	500 μg/l	AGS, Germany	AGS 2013
			end of exposure or end of shift	BAT-Suva	500 μg/l	Suva, Switzerland	Koller et al. 2018; SUVA 2025 a, b
			end of shift	BLV	1000 μg/l	SCOEL, EU Commission	SCOEL 2009 a
		urine	end of exposure or end of shift	VLB	200 μg/l	ANSES, France	ANSES 2017
				VBR	1.6 μg/l		
			end of shift	BLV	300 μg/l	SCOEL, EU Commission	SCOEL 2009 a
				BEI^®^	300 μg/l	BEI Committee, USA	ACGIH 2025
				OEL-B	200 μg/l	JSOH, Japan	JSOH 2023
	CO-Hb	blood	end of exposure or end of shift	BAT-Suva	5%	Suva, Switzerland	Koller et al. 2018; SUVA 2025 a, b
			end of shift	BLV	4%	SCOEL, EU Commission	SCOEL 2009 a
			immediately after exposure or at the end of a shift	VLB	3.5%^a)^	ANSES, France	ANSES 2017
				VBR	1.5%^a)^		
	CO	exhaled air	end of shift	BMGV	30 ppm	HSE, United Kingdom	HSE 2020, 2025
ethylbenzene	ethylbenzene	urine	end of shift	OEL-B	15 μg/l	JSOH, Japan	JSOH 2023
halothane (2‑bromo-2‑chloro-1,1,1‑tri­fluoro­ethane)	trifluoroacetic acid	blood	end of exposure or end of shift, for long-term exposure, at the end of a shift after several previous shifts	BGW	2.5 mg/l	AGS, Germany	AGS 2013
				BAT	2.5 mg/l	MAK Commission, Germany	DFG 2025
				BAT-Suva	2.5 mg/l	Suva, Switzerland	Koller et al. 2018; SUVA 2025 a, b
*n*-heptane	heptane-2,5‑dione	urine	end of exposure or end of shift	BAT	250 μg/l	MAK Commission, Germany	DFG 2025
*n*-hexane	hexane-2,5‑dione	urine	end of shift	BEI^®^	0.5 mg/g crea^c)^	BEI Committee, USA	ACGIH 2025
			end of shift at the end of the working week	OEL-B	3 mg/g crea^b)^	JSOH, Japan	JSOH 2023
					0.3 mg/g crea^d)^		
	hexane-1,2‑dione	urine	end of shift	BAL	0.57 mg/l	FIOH, Finnland	Kiilunen 1999
2-hexanone	hexane-2,5‑dione, without hydrolysis	urine	end of shift	BEI^®^	0.5 mg/l	BEI Committee, USA	ACGIH 2025
methanol	methanol	urine	end of exposure or end of shift	BGW	15 mg/l	AGS, Germany	AGS 2013
				BAT	15 mg/l	MAK Commission, Germany	DFG 2025
			end of exposure or end of shift; for long-term exposure, at the end of a shift after several previous shifts	BAT-Suva	30 mg/l	Suva, Switzerland	Koller et al. 2018; SUVA 2025 a, b
			end of shift	OEL-B	20 mg/l	JSOH, Japan	JSOH 2023
				BEI^®^	15 mg/l	BEI Committee, USA	ACGIH 2025
methyl *tert*‑butyl ether (2‑meth­oxy-2‑methylpropane)	methyl *tert*‑butyl ether	blood	end of exposure or end of shift	BAT	not established	MAK Commission, Germany	DFG 2025
			–	VLB	not established	ANSES, France	ANSES 2022
				VBR	not established		
		urine	end of exposure or end of shift	BAT	not established	MAK Commission, Germany	DFG 2025
	*tert‑*butanol	blood	–	BAT	not established	MAK Commission, Germany	DFG 2025
		urine	–	BAT	not established	MAK Commission, Germany	DFG 2025
methyl formate	methanol	urine	end of exposure or end of shift	BAT	not established	MAK Commission, Germany	DFG 2025
4-methyl­pentan-2-one (methyl isobutyl ketone)	4-methyl­pentan-2-one	urine	end of exposure or end of shift	BAT	0.7 mg/l	MAK Commission, Germany	DFG 2025
				BGW	0.7 mg/l	AGS, Germany	AGS 2013
				BAT-Suva	0.7 mg/l	Suva, Switzerland	Koller et al. 2018; SUVA 2025 a, b
			end of shift	BEI^®^	1 mg/l	BEI Committee, USA	ACGIH 2025
				OEL-B	1.7 mg/l	JSOH, Japan	JSOH 2023
				BMGV	20 μmol/l (2 mg/l)	HSE, United Kingdom	HSE 2020, 2025
2-propanol (isopropanol)	acetone	blood	end of exposure or end of shift	BAT	25 mg/l	MAK Commission, Germany	DFG 2025
				BGW	25 mg/l	AGS, Germany	AGS 2013
				BAT-Suva	25 mg/l	Suva, Switzerland	Koller et al. 2018; SUVA 2025 a, b
		urine	end of exposure or end of shift	BAT	25 mg/l	MAK Commission, Germany	DFG 2025
				BGW	25 mg/l	AGS, Germany	AGS 2013
				BAT-Suva	25 mg/l	Suva, Switzerland	Koller et al. 2018; SUVA 2025 a, b
			end of shift at the end of the working week	BEI^®^	40 mg/l	BEI Committee, USA	ACGIH 2025
styrene	styrene	urine	end of shift	BEI^®^	40 μg/l	BEI Committee, USA	ACGIH 2025
				VLB	40 μg/l	ANSES, France	ANSES 2014
			end of shift at the end of the working week	OEL-B	20 μg/l	JSOH, Japan	JSOH 2023
tetra­chloro­ethene	tetra­chloro­ethene	exhaled air	before the last shift	BEI^®^	3 ppm	BEI Committee, USA	ACGIH 2025
			before the last shift of the working week	BLV	3 ppm	SCOEL, EU Commission	SCOEL 2009 b
		blood	16 h after end of exposure	BAT	200 μg/l	MAK Commission, Germany	DFG 2025
				BGW	200 μg/l	AGS, Germany	AGS 2013
				EKA	60–1000 μg/l	MAK Commission, Germany	DFG 2025
			before the last shift of the working week	BLV	400 μg/l	SCOEL, EU Kommission	SCOEL 2009 b
			before the next shift	BAT-Suva	400 μg/l	Suva, Switzerland	Koller et al. 2018; SUVA 2025 a, b
			before the shift	BEI^®^	500 μg/l	BEI Committee, USA	ACGIH 2025
			before the shift at the end of the working week	BAL	1.0 mg/l	FIOH, Finnland	Kiilunen 1999
			in the morning after the working day	HTP	1.2 μmol/l (199 μg/l)	Ministry of Social Affairs and Health, Finland	STM 2025
			before the last shift of the working week	VLB	500 μg/l	ANSES, France	ANSES 2018
				VBR	0.12 μg/l		
		urine	end of shift at the end of the working week	VLB	50 μg/l	ANSES, France	ANSES 2018
				VBR	0.40 μg/l		
tetra­chloro­ethene	tri­chloro­acetic acid	urine	end of shift at the end of the working week	VGÜ limit value	40 mg/l	Federal Ministry of Labour and Economy, Austria	BAW 2024
tetra­chloro­methane (carbon tetrachloride)	tetra­chloro­methane	blood	end of shift; for long-term exposure after several previous shifts	BGW	3.5 μg/l	AGS, Germany	AGS 2013
				BAT	3.5 μg/l	MAK Commission, Germany	DFG 2025
tetra­hydro­furan	tetra­hydro­furan	urine	end of exposure or end of shift	BAT	2 mg/l	MAK Commission, Germany	DFG 2025
				BGW	2 mg/l	AGS, Germany	AGS 2013
				BAT-Suva	2 mg/l	Suva, Switzerland	Koller et al. 2018; SUVA 2025 a, b
			end of shift	BEI^®^	2 mg/l	BEI Committee, USA	ACGIH 2025
				OEL-B	2 mg/l	JSOH, Japan	JSOH 2023
toluene	toluene	blood	immediately after exposure	BAT	600 μg/l	MAK Commission, Germany	DFG 2025
				BGW	600 μg/l	AGS, Germany	AGS 2013
			end of exposure or end of shift	BAT-Suva	600 μg/l	Suva, Switzerland	Koller et al. 2018; SUVA 2025 a, b
			end of workday	VGÜ limit value	250 μg/l	Federal Ministry of Labour and Economy, Austria	BAW 2024
			before the last shift of the working week	BEI^®^	20 μg/l	BEI Committee, USA	ACGIH 2025
				VLB	20 μg/l	ANSES, France	ANSES 2011
				VBR	1 μg/l		
			before the shift at the end of the working week	BAL	92 μg/l	FIOH, Finnland	Kiilunen 1999
			in the morning after the working day	HTP	500 nmol/l (46 μg/l)	Ministry of Social Affairs and Health, Finland	STM 2025
			within 2 hours before the end of shift at the end of the working week	OEL-B	600 μg/l	JSOH, Japan	JSOH 2023
toluene	toluene	urine	end of exposure or end of shift	BAT	75 μg/l	MAK Commission, Germany	DFG 2025
				BGW	75 μg/l	AGS, Germany	AGS 2013
				BAT-Suva	75 μg/l	Suva, Switzerland	Koller et al. 2018; SUVA 2025 a, b
			end of shift	BEI^®^	30 μg/l	BEI Committee, USA	ACGIH 2025
				VLB	30 μg/l	ANSES, France	ANSES 2011
				VBR	0.4 μg/l		
			within 2 hours before the end of shift at the end of the working week	OEL-B	60 μg/l	JSOH, Japan	JSOH 2023
1,1,1-tri­chloro­ethane	1,1,1-tri­chloro­ethane	exhaled air	before the last shift of the working week	BEI^®^	20 ppm	BEI Committee, USA	ACGIH 2025
		blood	before the next shift after several previous shifts	BAT	275 μg/l	MAK Commission, Germany	DFG 2025
			after several previous shifts before the next shift	BGW	275 μg/l	AGS, Germany	AGS 2013
			for long-term exposure, at the end of a shift after several previous shifts	BAT-Suva	275 μg/l	Suva, Switzerland	Koller et al. 2018; SUVA 2025 a, b
			before the last shift of the working week	BAL	266 μg/l	FIOH, Finnland	Kiilunen 1999
		urine	end of shift	BEI^®^	700 μg/l	BEI Committee, USA	ACGIH 2025
tri­chloro­ethene	tri­chloro­ethene	exhaled air	–	BEI^®^^e)^	–	BEI Committee, USA	ACGIH 2025
		blood	–	BEI^®^^e)^	–	BEI Committee, USA	ACGIH 2025
		urine	end of shift	VLB	10 μg/l	ANSES, France	ANSES 2020
				VBR	1.5 μg/l		
tri­chloro­ethene	tri­chloro­acetic acid	urine	end of shift; for long-term exposure after several previous shifts	BAR	0.07 mg/l	MAK Commission, Germany	DFG 2025
				EKA	1.2–50 mg/l		
			end of exposure or end of shift; for long-term exposure, at the end of a shift after several previous shifts	BAT-Suva	40 mg/l	Suva, Switzerland	Koller et al. 2018; SUVA 2025 a, b
				Equi­valence value to the tol­er­ance con­centra­tion for carcinogenic substances	22 mg/l	AGS, Germany	AGS 2014
				Equivalence value to the acceptance concentration for carcinogenic substances	12 mg/l		
			at the end of the last shift of the working week	BLV	20 mg/l	SCOEL, EU Commission	SCOEL 2009 c
				BEI^®^	15 mg/l	BEI Committee, USA	ACGIH 2025
				VLB	15 mg/g crea (21 mg/l)	ANSES, France	ANSES 2020
				VBR	9 μg/g crea (8 μg/l)		
			at the end of the last shift of the working week	OEL-B	10 mg/l	JSOH, Japan	JSOH 2023
				BAL	59 mg/l	FIOH, Finnland	Kiilunen 1999
			end of exposure or end of shift	HTP	120 μmol/l (16 mg/l)	Ministry of Social Affairs and Health, Finland	STM 2025
	tri­chloro­ethanol	urine	at the end of the last shift of the working week	BEI^®^	0.5 mg/l	BEI Committee, USA	ACGIH 2025
1,1,2-tri­chloro-1,2,2‑tri­fluoro­ethane (Freon-113)	1,1,2-tri­chloro-1,2,2‑tri­fluoro­ethane	blood	at the end of the last shift of the working week	BAL	9.3 μg/l	FIOH, Finnland	Kiilunen 1999
xylene, all isomers	xylene, all isomers	blood	end of exposure or end of shift	BAT^f)^	1.5 mg/l	MAK Commission, Germany	DFG 2025
			at the end of the workday	VGÜ limit value	1.0 mg/l	Federal Ministry of Labour and Economy, Austria	BAW 2024

a) derived for non-smokers

b) with hydrolysis

c) without hydrolysis, not determined with headspace

d) without hydrolysis

e) semi-quantitative

f) until 2014

For abbreviations, see List of abbreviations.

**Tab.8:** Background exposure levels in the non-occupationally exposed general population

Analyte (synonym)	Matrix	Study collective	Number of persons	Reference value [μg l]			References
				Median	95^th^ percentile	Range	
acetonitrile	blood	healthy adults	28	30.6^a)^	n. a.	< 0.61–95.8	Mochalski et al. 2013
allyl methyl sulfide	blood	healthy adults	28	0.24^a)^	n. a.	< 0.003–1.91	Mochalski et al. 2013
*tert*‑amyl methyl ether	blood	healthy adults	3	< 0.0006	n. a.	< 0.0006	Silva et al. 2008
benzaldehyde	blood	healthy adults	28	< 0.27^a)^	n. a.	< 0.27	Mochalski et al. 2013
benzene	blood	non-smokers	15	0.087	n. a.	0.046–0.472	Perbellini et al. 2002
		smokers	10	0.246	n. a.	0.051–1.187	
		healthy adults	28	0.020^a)^	n. a.	< 0.001–0.077	Mochalski et al. 2013
		non-smokers	46	0.051	n. a.	0.034–0.113	Perbellini et al. 2003
		smokers	15	0.154	n. a.	0.046–0.487	
		adults	26	< 0.4	n. a.	< 0.4–2.61	Alonso et al. 2012
		non-smokers	24	0.180	n. a.	0.105–0.430	Andreoli et al. 1999
	urine	non-smokers	16	0.123	n. a.	n. a.	Fustinoni et al. 1999
		smokers	16	0.441	n. a.	n. a.	
		non-smokers	24	0.089	n. a.	0.045–0.353	Andreoli et al. 1999
		non-smokers	10	0.175	n. a.	< 0.050–0.291	Brčić Karačonji and Skender 2007
		smokers	10	0.502	n. a.	0.245–0.635	
		non-smokers	15	0.066	n. a.	0.024–0.248	Perbellini et al. 2002
		smokers	10	0.125	n. a.	0.042–0.409	
		non-smokers	10	21.4	n. a.	2.8–70.1	Song et al. 2017
		non-smokers	65	0.094	0.180	0.056–0.180^b)^	Fustinoni et al. 2010
		smokers	43	0.436	2.70	0.085–2.70^b)^	
		healthy men	90	0.146	2.23	0.043–2.23^b)^	Campo et al. 2016
		non-smokers	46	0.067	n. a.	0.026–0.531	Perbellini et al. 2003
		smokers	15	0.238	n. a.	0.045–1.099	
1,3-butadiene	urine	non-smokers	46	0.0011	n. a.	< 0.001–0.0024	Perbellini et al. 2003
		smokers	15	0.0031	n. a.	0.0012–0.0089	
	blood	healthy adults	28	0.009^a)^	n. a.	< 0.003–0.015	Mochalski et al. 2013
		non-smokers	46	0.0019	n. a.	< 0.0005–0.0035	Perbellini et al. 2003
		smokers	15	0.0060	n. a.	0.0012–0.0502	
*n*‑butane	blood	healthy adults	28	0.020^a)^	n. a.	< 0.008–0.027	Mochalski et al. 2013
2,3-butanedione (dimethyl glyoxal)	blood	healthy adults	28	< 0.34^a)^	n. a.	< 0.34	Mochalski et al. 2013
2-butanone (methyl ethyl ketone)	blood	healthy adults	28	2.52^a)^	n. a.	0.61–5.19	Mochalski et al. 2013
*n*-butylbenzene	urine	non-smokers	10	4.8	n. a.	3.1–9.1	Song et al. 2017
*c*-butylbenzene	urine	non-smokers	10	5.1	n. a.	4.4–5.7	Song et al. 2017
3-carene	blood	healthy adults	28	0.46^a)^	n. a.	< 0.12–0.60	Mochalski et al. 2013
(*m* + *p*)‑cresol	urine	non-smokers	10	23.0	n. a.	3.8–92.2	Song et al. 2017
*o*-cresol	urine	non-smokers	10	2.6	n. a.	2.1–4.8	Song et al. 2017
*n*-decane	blood	healthy adults	28	0.44^a)^	n. a.	< 0.043–1.88	Mochalski et al. 2013
di­chloro­methane (methylene chloride)	urine	healthy adults	120	0.64	n. a.	0.27–2.22	Poli et al. 2005
diisopropyl ether	blood	healthy adults	3	0.0057	n. a.	< 0.0006–0.044	Silva et al. 2008
2,3-dimethylbutane	blood	healthy adults	28	< 0.005^a)^	n. a.	< 0.005	Mochalski et al. 2013
2,5-dimethylfuran	urine	non-smokers	46	0.039	n. a.	< 0.005–0.290	Perbellini et al. 2003
		smokers	15	0.161	n. a.	0.019–0.525	
	blood	healthy adults	28	0.039^a)^	n. a.	< 0.002–0.063	Mochalski et al. 2013
		non-smokers	46	< 0.005	n. a.	< 0.005–0.040	Perbellini et al. 2003
		smokers	15	0.076	n. a.	< 0.005–0.373	
		adults	28	< 0.1	n. a.	< 0.1	Alonso et al. 2012
dimethyl selenide	blood	healthy adults	28	0.028^a)^	n. a.	< 0.003–0.055	Mochalski et al. 2013
dimethyl sulfide	blood	healthy adults	28	0.52^a)^	n. a.	0.12–2.04	Mochalski et al. 2013
ethyl acetate	blood	healthy adults	28	0.24^a)^	n. a.	< 0.009–0.44	Mochalski et al. 2013
ethylbenzene	blood	non-smokers	15	0.145	n. a.	< 0.022–0.496	Perbellini et al. 2002
		smokers	10	0.148	n. a.	0.063–0.596	
		healthy adults	28	0.208^a)^	n. a.	n. a.	Mochalski et al. 2013
		adults	28	< 0.2	n. a.	< 0.2–0.69	Alonso et al. 2012
		non-smokers	24	0.213	n. a.	0.145–0.880	Andreoli et al. 1999
	urine	non-smokers	16	0.030	n. a.	n. a.	Fustinoni et al. 1999
		smokers	16	0.057	n. a.	n. a.	
		healthy men	90	0.072	0.165	0.033–0.165^b)^	Campo et al. 2016
		non-smokers	24	0.073	n. a.	0.037–0.141	Andreoli et al. 1999
		non-smokers	10	0.121	n. a.	< 0.035–0.175	Brčić Karačonji and Skender 2007
		smokers	10	0.165	n. a.	0.070–0.353	
		non-smokers	15	0.0085	n. a.	< 0.017–0.047	Perbellini et al. 2002
		smokers	10	0.0085	n. a.	< 0.017–0.037	
		non-smokers	65	0.073	0.130	0.016–0.130^b)^	Fustinoni et al. 2010
		smokers	43	0.074	0.123	0.025–0.123^b)^	
ethyl *t*‑butyl ether	blood	healthy adults	3	< 0.0006	n. a.	< 0.0006–0.00066	Silva et al. 2008
	urine	non-smokers	65	< 0.015	0.024	< 0.015–0.024^b)^	Fustinoni et al. 2010
		smokers	43	< 0.015	0.025	< 0.015–0.025^b)^	
		healthy men	90	< 0.015	0.030	< 0.015–0.030^b)^	Campo et al. 2016
ethyl methyl sulfide	blood	healthy adults	28	0.030^a)^	n. a.	< 0.005–0.062	Mochalski et al. 2013
ethyl vinyl ether	blood	healthy adults	28	0.009^a)^	n. a.	< 0.003–0.017	Mochalski et al. 2013
eucalyptol	blood	healthy adults	28	1.00^a)^	n. a.	< 0.12–1.54	Mochalski et al. 2013
furan	blood	healthy adults	28	0.007^a)^	n. a.	< 0.0008–0.025	Mochalski et al. 2013
2-heptanone	blood	healthy adults	28	0.31^a)^	n. a.	0.069–0.65	Mochalski et al. 2013
4-heptanone	blood	healthy adults	28	0.095^a)^	n. a.	0.023–0.25	Mochalski et al. 2013
*cis*,*trans*‑2,4‑hexadiene	blood	healthy adults	28	< 0.002^a)^	n. a.	< 0.002	Mochalski et al. 2013
*n*‑hexane	blood	healthy adults	28	0.015^a)^	n. a.	< 0.002–0.049	Mochalski et al. 2013
2-hexanone	blood	healthy adults	28	0.036^a)^	n. a.	< 0.015–0.050	Mochalski et al. 2013
3-hexanone	blood	healthy adults	28	< 0.015^a)^	n. a.	< 0.015–0.048	Mochalski et al. 2013
1-hexene	blood	healthy adults	28	0.007^a)^	n. a.	< 0.002–0.018	Mochalski et al. 2013
isoprene	blood	healthy adults	28	1.00^a)^	n. a.	0.24–2.32	Mochalski et al. 2013
4-isopropyltoluene (*p*-cymene)	blood	healthy adults	28	0.15^a)^	n. a.	0.04–0.73	Mochalski et al. 2013
limonene	blood	healthy adults	28	1.27^a)^	n. a.	0.13–5.80	Mochalski et al. 2013
menthone	blood	healthy adults	28	0.76^a)^	n. a.	< 0.093–1.20	Mochalski et al. 2013
methyl acetate	blood	healthy adults	28	2.26^a)^	n. a.	0.25–11.6	Mochalski et al. 2013
2-methylbutane (isopentane)	blood	healthy adults	28	0.053^a)^	n. a.	< 0.005–0.152	Mochalski et al. 2013
2-methyl-1-butene	blood	healthy adults	28	< 0.004^a)^	n. a.	< 0.004	Mochalski et al. 2013
methyl *t*‑butyl ether (2-methoxy-2‑methylpropane)	blood	healthy adults	3	0.0029	n. a.	0.0022–0.0035	Silva et al. 2008
	urine	non-smokers	65	0.046	0.152	0.020–0.152^b)^	Fustinoni et al. 2010
		smokers	43	0.051	0.097	0.023–0.097^b)^	
		healthy men	90	0.070	0.219	< 0.010–0.219^b)^	Campo et al. 2016
2-methylfuran	blood	healthy adults	28	0.012^a)^	n. a.	< 0.0008–0.021	Mochalski et al. 2013
3-methylfuran	blood	healthy adults	28	0.005^a)^	n. a.	< 0.001–0.008	Mochalski et al. 2013
2-methylhexane	blood	healthy adults	28	0.013^a)^	n. a.	< 0.002–0.057	Mochalski et al. 2013
4-methyloctane	blood	healthy adults	28	0.12^a)^	n. a.	< 0.019–0.31	Mochalski et al. 2013
2-methyl­pentane	blood	healthy adults	28	0.030^a)^	n. a.	< 0.007–0.046	Mochalski et al. 2013
4-methyl-1-pentene	blood	healthy adults	28	< 0.003^a)^	n. a.	< 0.003	Mochalski et al. 2013
2-methylpropane (isobutane)	blood	healthy adults	28	0.07^a)^	n. a.	< 0.013–0.09	Mochalski et al. 2013
2-methyl-1-propene (isobutene)	blood	healthy adults	28	0.19^a)^	n. a.	n. a.	Mochalski et al. 2013
2-methyl-2-propenal	blood	healthy adults	28	< 0.063^a)^	n. a.	< 0.063	Mochalski et al. 2013
methyl propionate	blood	healthy adults	28	0.25^a)^	n. a.	< 0.012–1.32	Mochalski et al. 2013
methyl propyl sulfide	blood	healthy adults	28	0.40^a)^	n. a.	< 0.004–6.89	Mochalski et al. 2013
1‑methylpyrrole	blood	healthy adults	28	0.039^a)^	n. a.	< 0.008–0.049	Mochalski et al. 2013
*α*-methylstyrene	blood	healthy adults	28	0.024^a)^	n. a.	< 0.012–0.024	Mochalski et al. 2013
3-methylthiophene	blood	healthy adults	28	< 0.002^a)^	n. a.	< 0.002–0.004	Mochalski et al. 2013
methyl vinyl ketone (butenone)	blood	healthy adults	28	10.9^a)^	n. a.	< 2.8–12.7	Mochalski et al. 2013
naphthalene	urine	non-smokers	10	9.5	n. a.	2.3–22.9	Song et al. 2017
		non-smokers	7	0.048	0.057	0.038–0.057^b)^	Fustinoni et al. 2010
		smokers	11	0.044	0.266	0.038–0.266^b)^	
*n*-octane	blood	healthy adults	28	0.15^a)^	n. a.	< 0.005–0.39	Mochalski et al. 2013
pentane	blood	healthy adults	28	0.027^a)^	n. a.	< 0.007–0.058	Mochalski et al. 2013
*cis*‑1,3‑pentadiene	blood	healthy adults	28	< 0.001^a)^	n. a.	< 0.001	Mochalski et al. 2013
*trans*‑1,3‑pentadiene	blood	healthy adults	28	0.006^a)^	n. a.	< 0.002–0.007	Mochalski et al. 2013
*cis*‑pent‑2‑ene	blood	healthy adults	28	< 0.003^a)^	n. a.	< 0.003	Mochalski et al. 2013
*trans*‑pent‑2‑ene	blood	healthy adults	28	0.009^a)^	n. a.	< 0.003–0.009	Mochalski et al. 2013
2-pentanone	blood	healthy adults	28	2.99^a)^	n. a.	0.81–9.08	Mochalski et al. 2013
*trans*‑3‑penten‑2‑one	blood	healthy adults	28	0.84^a)^	n. a.	< 0.21–1.71	Mochalski et al. 2013
*α*-pinene	blood	healthy adults	28	< 0.008^a)^	n. a.	< 0.008	Mochalski et al. 2013
*β*-pinene	blood	healthy adults	28	0.15^a)^	n. a.	< 0.005–0.20	Mochalski et al. 2013
propanal	blood	healthy adults	28	0.93^a)^	n. a.	< 0.076–1.68	Mochalski et al. 2013
propene (propylene)	blood	healthy adults	28	0.59^a)^	n. a.	0.16–2.59	Mochalski et al. 2013
2-propenal (acrolein)	blood	healthy adults	28	137^a)^	n. a.	< 15.1–376	Mochalski et al. 2013
propylbenzene	urine	non-smokers	10	4.0	n. a.	2.0–5.8	Song et al. 2017
pyrazine	blood	healthy adults	28	1.60^a)^	n. a.	< 0.36–2.56	Mochalski et al. 2013
pyrrole	blood	healthy adults	28	0.070^a)^	n. a.	< 0.001–0.127	Mochalski et al. 2013
styrene	blood	healthy adults	28	0.037^a)^	n. a.	< 0.010–0.076	Mochalski et al. 2013
		adults	28	< 0.100	n. a.	< 0.100–0.600	Alonso et al. 2012
tetra­chloro­ethene	urine	healthy adults	120	0.05	n. a.	0.01–0.70	Poli et al. 2005
thiophene	blood	healthy adults	28	0.004^a)^	n. a.	< 0.001–0.012	Mochalski et al. 2013
toluene	blood	non-smokers	15	0.428	n. a.	0.120–6.040	Perbellini et al. 2002
		smokers	10	0.780	n. a.	0.348–5.148	
		healthy adults	28	0.055^a)^	n. a.	< 0.003–0.29	Mochalski et al. 2013
		adults	28	1.15	n. a.	< 0.2–3.10	Alonso et al. 2012
		non-smokers	24	0.285	n. a.	0.105–0.925	Andreoli et al. 1999
	urine	non-smokers	16	0.215	n. a.	n. a.	Fustinoni et al. 1999
		smokers	16	0.336	n. a.	n. a.	
		non-smokers	24	0.280	n. a.	0.155–0.480	Andreoli et al. 1999
		non-smokers	10	0.166	n. a.	0.141–0.216	Brčić Karačonji and Skender 2007
		smokers	10	0.633	n. a.	0.184–0.886	
		non-smokers	15	0.416	n. a.	0.143–1.227	Perbellini et al. 2002
		smokers	10	0.259	n. a.	0.131–0.856	
		non-smokers	10	3.6	n. a.	2.3–4.9	Song et al. 2017
		non-smokers	65	0.375	0.506	0.092–0.506^b)^	Fustinoni et al. 2010
		smokers	43	0.437	0.698	0.126–0.698^b)^	
		healthy men	90	0.251	0.738	0.172–0.738^b)^	Campo et al. 2016
tri­chloro­ethene	urine	healthy adults	120	0.22	n. a.	0.02–3.64	Poli et al. 2005
*n*-undecane	blood	healthy adults	28	0.34^a)^	n. a.	< 0.11–0.41	Mochalski et al. 2013
*m*-xylene	blood	non-smokers	15	0.535	n. a.	0.092–1.451	Perbellini et al. 2002
		smokers	10	0.411	n. a.	0.203–1.713	
	urine	non-smokers	15	0.099	n. a.	0.072–0.184	Perbellini et al. 2002
		smokers	10	0.079	n. a.	0.063–0.171	
(*m* + *p*)‑xylene	blood	healthy adults	28	0.10^a)^	n. a.	< 0.007–1.19	Mochalski et al. 2013
		adults	28	< 0.300	n. a.	< 0.300–1.750	Alonso et al. 2012
	urine	non-smokers	16	0.108	n. a.	n. a.	Fustinoni et al. 1999
		smokers	16	0.163	n. a.	n. a.	
		non-smokers	10	0.329	n. a.	0.104–0.465	Brčić Karačonji and Skender 2007
		smokers	10	0.436	n. a.	0.198–0.901	
		non-smokers	65	0.124	0.165	0.050–0.165^b)^	Fustinoni et al. 2010
		smokers	43	0.128	0.215	0.055–0.215^b)^	
		healthy men	90	0.110	0.237	0.063–0.237^b)^	Campo et al. 2016
(*m* + *o* + *p*)-xylene	blood	non-smokers	24	0.722	n. a.	0.280–1.342	Andreoli et al. 1999
	urine	non-smokers	24	0.220	n. a.	0.120–0.459	Andreoli et al. 1999
*o*-xylene	blood	healthy adults	28	0.23^a)^	n. a.	< 0.009–0.55	Mochalski et al. 2013
		adults	28	< 0.2	n. a.	< 0.2	Alonso et al. 2012
	urine	non-smokers	16	0.043	n. a.	n. a.	Fustinoni et al. 1999
		smokers	16	0.061	n. a.	n. a.	
		non-smokers	10	0.042	n. a.	< 0.042–0.104	Brčić Karačonji and Skender 2007
		smokers	10	0.096	n. a.	0.060–0.213	
		non-smokers	65	0.044	0.060	0.017–0.060^b)^	Fustinoni et al. 2010
		smokers	43	0.042	0.079	0.019–0.079^b)^	
		healthy men	90	0.037	0.082	0.020–0.082^b)^	Campo et al. 2016

a) mean

b) 5^th^–95^th^ percentile

**Tab.9:** U.S. reference values from the non-occupationally exposed general population for parameters which can be measured by headspace methods

Substance (synonym)	Analyte	Matrix	Study collective	Reference value^a)^ [μg/l]	Survey period	References
benzene	benzene	blood	general population > 18 a, smoker	0.642	2015/2016	NCEH 2021 a
			general population > 18 a, non-smoker	0.067	2015/2016	NCEH 2021 a
chlorobenzene	chlorobenzene	blood	general population > 18 a, smoker	< LOD (0.011)	2015/2016	NCEH 2021 a
			general population > 18 a, non-smoker	< LOD (0.011)	2015/2016	NCEH 2021 a
1,1-dichloroethane	1,1-dichloroethane	blood	general population > 20 a	< LOD (0.010)	2011/2012	NCEH 2021 b
1,2-dichloroethane	1,2-dichloroethane	blood	general population > 18 a, smoker	< LOD (0.010)	2015/2016	NCEH 2021 a
			general population > 18 a, non-smoker	< LOD (0.010)	2015/2016	NCEH 2021 a
di­chloro­methane (methylene chloride)	di­chloro­methane	blood	general population > 18 a, smoker	< LOD (0.250)	2015/2016	NCEH 2021 a
			general population > 18 a, non-smoker	< LOD (0.250)	2015/2016	NCEH 2021 a
1,4-dioxane	1,4-dioxane	blood	general population > 18 a, smoker	< LOD (0.500)	2015/2016	NCEH 2021 a
			general population > 18 a, non-smoker	< LOD (0.500)	2015/2016	NCEH 2021 a
ethylbenzene	ethylbenzene	blood	general population > 18 a, smoker	0.202	2015/2016	NCEH 2021 a
			general population > 18 a, non-smoker	0.056	2015/2016	NCEH 2021 a
*n*-hexane	*n*-hexane	blood	general population > 18 a, smoker	< LOD (0.122)	2015/2016	NCEH 2021 a
			general population > 18 a, non-smoker	< LOD (0.122)	2015/2016	NCEH 2021 a
isopropylbenzene (cumene)	isopropylbenzene	blood	general population > 18 a, smoker	< LOD (0.040)	2015/2016	NCEH 2021 a
			general population > 18 a, non-smoker	< LOD (0.040)	2015/2016	NCEH 2021 a
methyl *tert*‑butyl ether (2-methoxy-2‑methyl­propane)	methyl *tert*‑butyl ether	blood	general population > 18 a, smoker	10.0	2015/2016	NCEH 2021 a
			general population > 18 a, non-smoker	< LOD (0.010)	2015/2016	NCEH 2021 a
methylmercury	methylmercury	blood	general population > 20 a	4.42	2015/2016	NCEH 2025 a
styrene	styrene	blood	general population > 20 a	0.146	2009/2010	NCEH 2025 b
1,1,1,2-tetrachloroethane	1,1,1,2-tetrachloroethane	blood	general population > 18 a, smoker	< LOD (0.040)	2015/2016	NCEH 2021 a
			general population > 18 a, non-smoker	< LOD (0.040)	2015/2016	NCEH 2021 a
1,1,2,2-tetrachloroethane	1,1,2,2-tetrachloroethane	blood	general population > 20 a	< LOD (0.010)	2011/2012	NCEH 2021 b
tetra­chloro­ethene	tetra­chloro­ethene	blood	general population > 18 a, smoker	0.056	2015/2016	NCEH 2021 a
			general population > 18 a, non-smoker	0.084	2015/2016	NCEH 2021 a
tetra­chloro­methane (carbon tetrachloride)	tetra­chloro­methane	blood	general population > 18 a, smoker	< LOD (0.005)	2015/2016	NCEH 2021 a
			general population > 18 a, non-smoker	< LOD (0.005)	2015/2016	NCEH 2021 a
tetra­hydro­furan	tetra­hydro­furan	blood	general population > 18 a, smoker	< LOD (0.125)	2015/2016	NCEH 2021 a
			general population > 18 a, non-smoker	< LOD (0.125)	2015/2016	NCEH 2021 a
toluene	toluene	blood	general population > 18 a, smoker	1.50	2015/2016	NCEH 2021 a
			general population > 18 a, non-smoker	0.312	2015/2016	NCEH 2021 a
1,1,1-tri­chloro­ethane	1,1,1-tri­chloro­ethane	blood	general population > 18 a, smoker	< LOD (0.010)	2015/2016	NCEH 2021 a
			general population > 18 a, non-smoker	< LOD (0.010)	2015/2016	NCEH 2021 a
1,1,2-tri­chloro­ethane	1,1,2-tri­chloro­ethane	blood	general population > 20 a	< LOD (0.010)	2011/2012	NCEH 2021 b
tri­chloro­ethene	tri­chloro­ethene	blood	general population > 18 a, smoker	< LOD (0.012)	2015/2016	NCEH 2021 a
			general population > 18 a, non-smoker	< LOD (0.012)	2015/2016	NCEH 2021 a
tri­chloromethane	tri­chloromethane	blood	general population > 18 a, smoker	0.053	2015/2016	NCEH 2021 a
			general population > 18 a, non-smoker	0.047	2015/2016	NCEH 2021 a
(*m* + *p*)‑xylene	(*m* + *p*)‑xylene	blood	general population > 18 a, smoker	0.582	2015/2016	NCEH 2021 a
			general population > 18 a, non-smoker	0.213	2015/2016	NCEH 2021 a
*o*-xylene	*o*-xylene	blood	general population > 18 a, smoker	0.106	2015/2016	NCEH 2021 a
			general population > 18 a, non-smoker	0.059	2015/2016	NCEH 2021 a

a) 95^th^ percentile

For abbreviations, see List of abbreviations.

## Figures and Tables

**Tab.10:** Test conditions for investigating the storage stability of various solvents

Material	Pooled EDTA blood				Pooled urine			
**Sample volume**	1 ml	1 ml	1 ml	1 ml	1 ml	1 ml	1 ml	1 ml
**NaCl**	1 g	1 g	–	–	1 g	1 g	–	–
**Specimen vessel/ vial**	crimp cap headspace vial		blood tube (glass)	screw-cap vial	crimp cap headspace vial		urine cup	screw-cap vial
**HgCl**_**2**_ ** solution**	100 μl	–	–	–	100 μl	–	–	–
**Temperature**	22 °C	22 °C	4 °C	–20 °C	22 °C	22 °C	4 °C	–20 °C
**Duration**	14 d	14 d	14 d	14 d	14 d	14 d	14 d	14 d
**Measuring in**	storage vessel		crimp cap headspace vial		storage vessel		crimp cap headspace vial	

**Table:** 

Equilibration:	20 min at 70 °C
Temperature of the transfer line to the GC:	140 °C
Loop temperature:	130 °C

**Table:** 

Capillary column:	Fused silica (Restek Rxi‑624Sil MS, 60 m × 0.25 mm × 1.4 μm)
Temperature programme:	Starting temperature 35 °C for 2 min, increase at 10 °C/min to 190 °C, then increase at 25 °C/min to 300 °C, 4 min at final temperature
Injector temperature:	250 °C
Carrier gas:	Helium 5.0; Flow rate: 1.7 ml/min
Injection:	Split: 6 ∶ 1

**Table:** 

Ionisation type:	EI+
Interface temperature:	280 °C
Source temperature:	230 °C
Detection mode:	Single Ion Monitoring (SIM)

**Tab.11:** Retention times and mass fragments of the measured analytes

Analyte	Retention time [min]	Q1 (*m/z*)	Q2 (*m/z*)
acetone	6.34	58	43
2-propanol	6.49	45	43
acetonitrile	6.67	41	39
1-propanol	7.86	59	42
2‑butanone (methyl ethyl ketone)	8.56	72	57
tetra­hydro­furan	9.01	72	71
isobutanol	9.41	74	43
1-butanol	10.2	56	41
cyclohexanone	13.0	98	55
cyclohexanol	15.2	82	57

**Fig.6:**
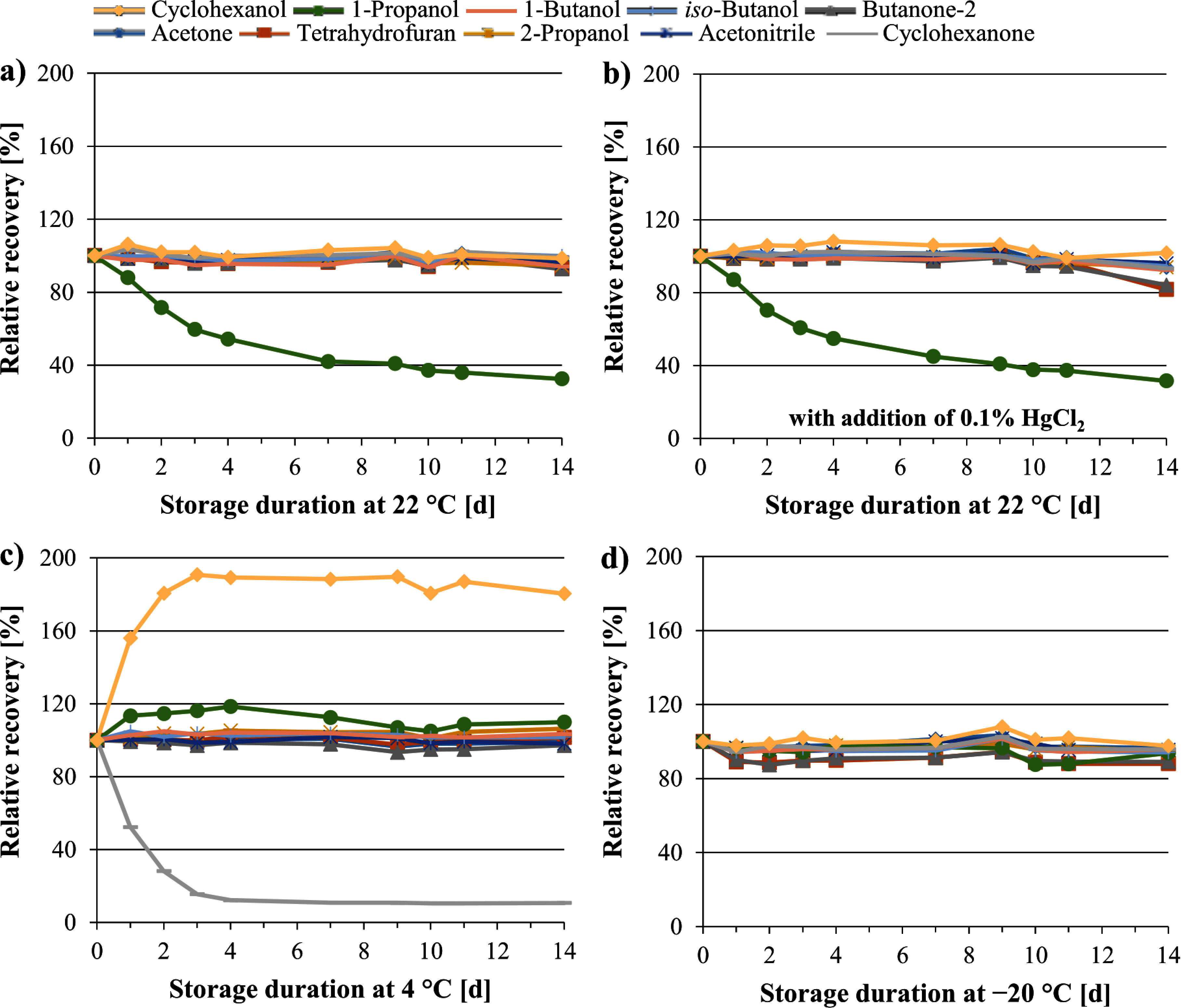
Relative recoveries of the analytes after storage of the spiked blood samples: a) storage in crimp cap headspace vials at 22 °C with addition of 1 g NaCl, b) storage in crimp cap headspace vials at 22 °C with addition of 1 g NaCl and 100 μl 0.1% HgCl_2_ solution, c) storage in EDTA blood collection tubes made of glass at 4 °C, and d) storage in screw cap vials at −20 °C

**Fig.7:**
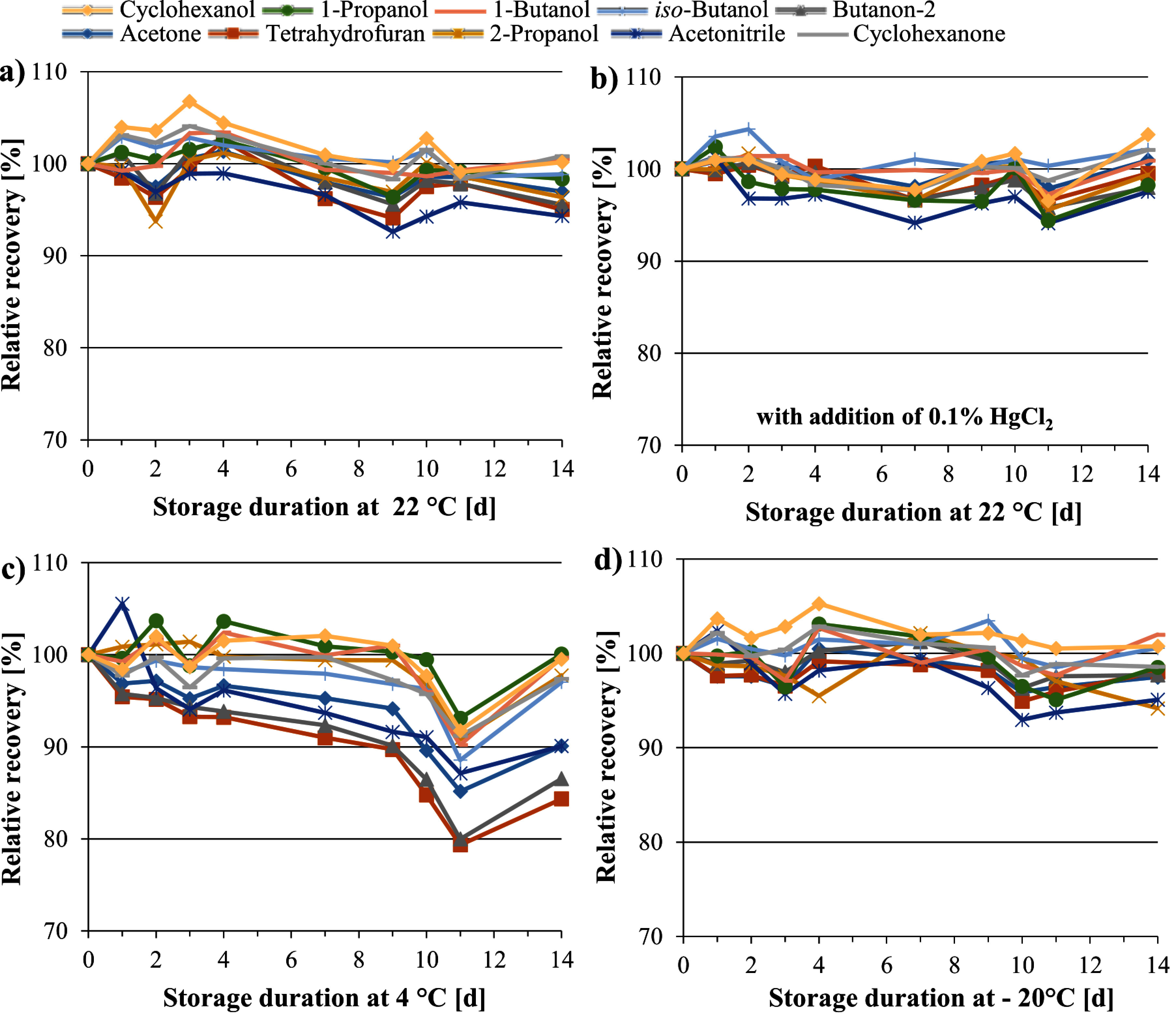
Relative recoveries of the analytes after storage of the spiked urine samples: a) storage in crimp cap headspace vials at 22 °C with the addition of 1 g NaCl, b) storage in crimp cap headspace vials at 22 °C with the addition of 1 g NaCl and 100 μl 0.1% HgCl_2_ solution, c) storage in a urine cup at 4 °C, and d) storage in screw cap vials at −20 °C

**Table:** 

ACGIH	American Conference of Governmental Industrial Hygienists
AGS	Committee on Hazardous Substances (“Ausschuss für Gefahrstoffe”)
ANSES	French Agency for Food Safety, Environment and Labour Protection (“Agence nationale de sécurité sanitaire de l’alimentation, de l’environnement et du travail”)
BAL	biomonitoring action level
BAR	biological reference value (“Biologischer Arbeitsstoff-Referenzwert”)
BAT	biological tolerance value (“Biologischer Arbeitsstoff-Toleranzwert”)
BAT-Suva	biological tolerance value (“Biologischer Arbeitsstoff-Toleranzwert”) by Suva
BEI^®^	biological exposure index
BGV	biological guidance value
BGW	biological limit value (“Biologischer Grenzwert”)
BLV	biological limit value (“Biologischer Grenzwert”)
BLW	biological guidance value (“Biologischer Leitwert”)
BMGV	biological monitoring guidance value
BTEX	benzene, toluene, ethylbenzene and isomeric xylenes
2-CP	2-chlorophenol
CDC	Centers for Disease Control and Prevention
2,4‑DCP	2,4-dichlorophenol
DDD	dichlorodiphenyldichloroethane
DDE	dichlorodiphenyldichloroethylene
DDT	dichlorodiphenyltri­chloro­ethane
ECD	electrochemical detection
EKA	exposure equivalents for carcinogenic substances (“Expositionsäquivalente für krebserzeugende Arbeitsstoffe”)
FID	flame ionisation detector
FIOH	Finnish Institute of Occupational Health
GC-MS	gas chromatography with mass spectrometric detection
G-EQUAS	German Quality Assessment Scheme
Hb	haemoglobin
HBM	human biomonitoring
HCB	hexachlorobenzene
*β*-HCH	*β*-hexachlorocyclohexane
HNMF	*N*-hydroxymethyl-*N*-methylformamide
HS-Cryotrapping-GC-MS	headspace cryotrapping gas chromatography with mass spectrometric detection
HSE	Health and Safety Executive
HS-GC	headspace gas chromatography
HS-GC-ECD	headspace gas chromatography with electrochemical detection
HS-GC-FID	headspace gas chromatography with flame ionisation detection
HS-GC-FID-MS	headspace gas chromatography with flame ionisation detection and mass spectrometric detection
HS-GC-MS	headspace gas chromatography with mass spectrometric detection
HS-GC-MS/MS	headspace gas chromatography with tandem mass spectrometry
HS-GC-NPD	headspace gas chromatography with nitrogen-phosphorus detection
HS-GC-TCD	headspace gas chromatography with thermal conductivity detector
HS-NTD-GC-FID	headspace-needle-trap-device gas chromatography with flame ionisation detection
HS-NTD-GC-MS	headspace-needle-trap-device gas chromatography with mass spectrometric detection
HSSE	headspace sorptive extraction
HSSE-GC-MS	headspace-sorptive-extraction gas chromatography with mass spectrometric detection
HS-SPME	headspace-solid phase microextraction
HS-SPME-GC-FID	headspace-solid phase microextraction gas chromatography with flame ionisation detection
HS-SPME-GC-HRMS	headspace-solid phase microextraction with high resolution mass spectrometric detection
HS-SPME-GC-IT/MS	headspace-solid phase microextraction gas chromatography with ion trap/mass spectrometric detection
HS-SPME-GC-MS	headspace-solid phase microextraction gas chromatography with mass spectrometric detection
HTP	concentrations known to be harmful (“Haitallisiksi tunnetut pitoisuudet”)
ISTD	internal standard
ITEX	in-tube extraction technique
JSOH	Japan Society for Occupational Health
LOD	limit of detection
MS	mass spectrometric
MTBE	methyl *tert*‑butyl ether
n. a.	not available
NHANES	National Health and Nutrition Examination Survey
OEL-B	occupational exposure limit based on biological monitoring
PCB	polychlorinated biphenyls
PCP	pentachlorophenol
PDMS	polydimethylsiloxane
POP	persistent organic pollutant
PT-HS-GC-HRMS	purge-and-trap headspace gas chromatography with high resolution mass spectrometric detection
PT-HS-GC-MS	purge-and-trap headspace gas chromatography with mass spectrometric detection
PT-HS-GC-PID	purge-and-trap headspace gas chromatography with photoionisation detection
RAC	ECHA Committee for Risk Assessment
SBSE	stir-bar sorptive extraction
SCOEL	Scientific Committee on Occupational Exposure Limits
SDME	single-drop microextraction
SPDE	solid phase dynamic extraction
SPME	solid phase microextraction
2,3,4,6‑TeCP	2,3,4,6-tetrachlorophenol
2,4,6‑TCP	2,4,6-tri­chlorophenol
THF	tetra­hydro­furan
TLHS-DAI-GC-ECD	thin-layer headspace direct aqueous injection gas chromatography with electrochemical detection
VBR	biological reference values (“valeurs biologiques de reference”)
VGÜ-Grenzwert	ordinance on health surveillance at the workplace
VLB	biological limit values (“valeurs limites biologiques”)
VOC	volatile organic compound

## Appendix

### Determination of the storage stability of various solvents in blood and urine

Samples for headspace analysis should always be analysed as soon as possible after sampling. In daily practice, however, delays due to sample logistics (e.g. sampling before the weekend, delayed sample transport) or analysis (large number of samples, equipment failure, staff shortages, etc.) cannot be ruled out.

Storage-stability tests were conducted to investigate the influence of transport and storage conditions on analyte recovery. For this purpose, blood and urine samples were spiked with ten different solvents (acetone, tetra­hydro­furan, 2‑butanone (methyl ethyl ketone), 2‑propanol, acetonitrile, 1‑propanol, isobutanol, 1‑butanol, cyclohexanone, and ­cyclohexanol) and stored under different conditions. The solvents tested were based on the routine spectrum of solvents that the analysing laboratory usually investigates.

Pooled EDTA blood and pooled urine from persons not occupationally exposed to these solvents were used as the test matrix. The material was spiked with the ten analytes at a concentration of 10 mg/l each and aliquoted into headspace-crimp cap vials, screw-cap vials or the respective specimen container. Mercury(II) chloride was added to some samples to inhibit bacterial growth. The samples were stored at different temperatures for 14 days and examined ten times during this period using headspace GC‑MS. The samples stored in EDTA blood collection tubes, urine cups or screw-cap vials were pipetted into headspace-crimp cap vials before measurement. The different test conditions are summarised in Table 10.

### Measurement conditions

Analytical determination was carried out using an HS‑GC‑MS device from Agilent consisting of a headspace sampler (Agilent G1888), a gas chromatograph (Agilent GC 7890A) and a mass-selective detector (Agilent 5975C).

#### Mass spectrometry

The retention times and mass fragments (Q1, Q2) of the analytes are listed in Table 11:

### Measurement results

The background levels in the blood and urine samples used were measured and subtracted from the analyte levels in the spiked samples, so the results shown are blank-corrected. The samples were quantified using external calibration. The relative recoveries of the analytes in the blood samples are shown in Figure 6, and those in the urine samples are shown in Figure 7.

### Discussion of results

#### Solvents in blood

No significant solvent depletion was observed in the samples stored at room temperature in sealed headspace vials over the selected period of time. The only exception was 1‑propanol, whose concentration continuously decreased to about 30% of the initial concentration. A potential cause could be residual enzymatic activity in the blood, which could have caused oxidation to 1‑propanal and, in turn, to propionic acid. This could be due to haemolysis caused by the addition of NaCl, resulting in the release of erythrocyte components (e.g. haem). The HgCl_2_ solution added to the samples stored at 22 °C to prevent enzymatic and/or bacterial influences showed no effect.

Completely different effects were observed when the spiked blood samples were stored in the blood collection tubes. While most solvents showed no significantly reduced concentration in the samples stored at 4 °C, a clear decrease was observed for cyclohexanone, which was reduced to cyclohexanol. Residual enzymatic activity is suspected to be the cause of this reduction, which was not observed in samples stored at −20 °C. In samples stored at 22 °C, the reduction of cyclohexanone did not occur, probably due to the added sodium chloride.

#### Solvents in urine

No significant loss of analytes was detected in the spiked pooled urine samples stored under the selected conditions. Both in the samples filled directly into headspace vials and in the samples stored at −20 °C, the relative recovery was between 93 and 107%. There was no difference in analyte recoveries between samples stored with and without HgCl_2 _addition.

In comparison, urine samples stored at 4 °C in urine cups showed analyte losses of up to 20%. In these measurements, it could not be ruled out that losses occurred due to solvent outgassing during repeated sampling of aliquots. However, even under these storage conditions, the analytical recoveries over a period of seven days can be regarded as good.

### Summary

Under the storage conditions tested here, the solvents examined were stable in the spiked blood samples at −20 °C for 14 days. At room temperature, 1‑propanol degraded even with the addition of HgCl_2_, and cyclohexanone degraded without the addition of NaCl.

In the urine samples, the analyte recoveries can be described as good both when the samples were stored in headspace vials at room temperature and when the samples were stored in screw cap vials at −20 °C in the freezer. For samples stored in urine cups in the refrigerator, slightly fluctuating recovery rates were observed for a large number of analytes; it is thus recommended that the analytes be determined within seven days.

### General information

To evaluate the different blood collection systems, the use of Vacutainers^®^ as an alternative to Monovettes^®^ was intensively investigated. When several types of Vacutainers^®^ with different types of stoppers were tested in the context of BTX analysis, concentrations of up to 5 μg benzene per litre of blood were detected (Bader et al. 1994). It was possible to prove that the blank values could be reduced to the low level of Monovettes^®^ by using specially prepared Vacutainer^®^ stoppers (purple stoppers on Vacutainer^®^ Art. BD 367655).

When storing blood samples for the analysis of volatile halogenated hydrocarbons (VHH), it was observed that the stability of carbon tetrachloride in particular could be increased if the samples were stored in the dark.

When evaluating abnormal 2‑propanol findings in blood, possible contamination of the puncture site with 2‑propanol‑containing disinfectants must be considered.

When storing crimp cap headspace vials at 4 °C, but especially at −20 °C, it is essential to take into account the different coefficients of expansion of the materials used. It has been observed that the aluminium caps of the headspace vials can be turned freely as early as the next day. This can lead to possible losses as well as contamination.

Ogawa and Sasahara (2012) found that short-term refrigerated storage of blood samples (up to three days) did not result in signifi­cant analyte losses. In another study investigating di­chloro­methane in urine, no significant differences were found between storage at room temperature and in the refrigerator (Hoffer et al. 2005). However, it is important in all cases to transfer the samples quickly to gas-tight sample vials (Hoffer et al. 2005; Ogawa and Sasahara 2012).

The addition of sodium chloride can be applied to prevent fungal growth in urine samples stored at room temperature. Internal investigations have shown a loss of methanol in urine samples not stabilised with sodium chloride, in contrast to samples mixed with sodium chloride.
